# Combined directed *ortho*-zincation and palladium-catalyzed strategies: Synthesis of 4,n-dimethoxy-substituted benzo[*b*]furans

**DOI:** 10.3762/bjoc.7.146

**Published:** 2011-09-12

**Authors:** Verónica Guilarte, M Pilar Castroviejo, Estela Álvarez, Roberto Sanz

**Affiliations:** 1 Área de Química Orgánica, Departamento de Química, Facultad de Ciencias, Universidad de Burgos, Pza. Misael Bañuelos s/n, 09001 Burgos, Spain

**Keywords:** benzo[*b*]furans, *o*-zincation, palladium, selectivity

## Abstract

A new route to regioselectively dialkoxy-functionalized benzo[*b*]furan derivatives has been developed from 3-halo-2-iodoanisoles bearing an additional methoxy group, which have been accessed through an *ortho*-zincation/iodination reaction. Two palladium-catalyzed processes, namely a Sonogashira coupling followed by a tandem hydroxylation/cyclization sequence, give rise to new and interesting dimethoxy-substituted benzo[*b*]furans.

## Introduction

The directed *ortho*-metallation (D*o*M) reaction has been widely used as a powerful and efficient method for regioselective functionalization of aromatic compounds and different directing groups have been used to facilitate the deprotonation reaction [1–5]. Various strong bases such as alkyl lithiums and their derivatives (for instance, TMEDA-activated complexes [6] and heavier alkali metal *tert*-butoxide-complexed alkyl lithium reagents, known as superbases and introduced by Schlosser [7]), as well as lithium dialkylamides, have usually been employed to perform deprotonative metallations. Whereas the use of these strong bases has several limitations regarding the presence of certain functional groups (mainly carboxylic acid derivatives and halogens), the introduction in the last years of new organometallic “ate” complexes [8–9], which combine an alkali metal with either magnesium, zinc, aluminium, or copper, has allowed more selective metallation reactions. The milder reaction conditions required make these deprotonation reactions tolerant to the coexistence of a wider range of functional groups. In this field, the work of Kondo and Uchiyama is remarkable as they described highly chemo- and regioselective deprotonative zincation [10–12], alumination [13], and cupration [14] reactions of some functionalized aromatic and heteroaromatic compounds, as well as of *meta*-functionalized haloaromatics. In particular, the alkali metal mediated zincation reactions have turned out to be very useful processes and the structures and reaction pathways of TMP-zincates (TMP = 2,2,6,6-tetramethylpiperidine) have been studied in detail [15–19].

**Figure 1:**
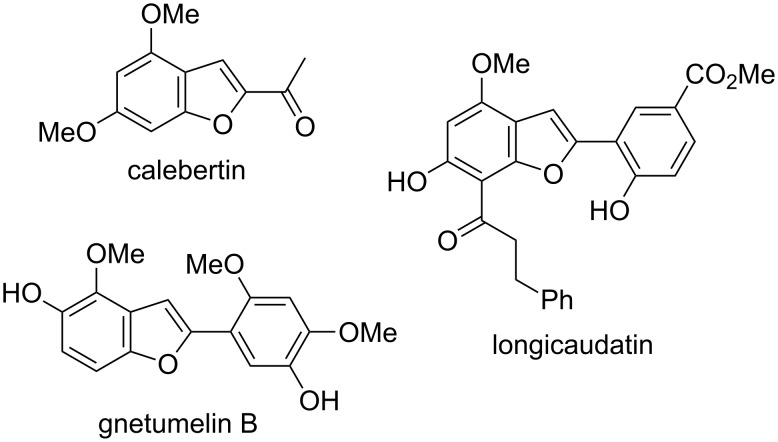
Some representative dihydroxybenzofuran derived natural products.

On the other hand, benzo[*b*]furan is a basic skeleton found in a variety of significant natural products [20], and some derivatives are also used as organic materials, due to their optical and electronic properties [21]. Thus, many synthetic efforts have been devoted to the synthesis of this type of compound [22–23]. In particular, several benzo[*b*]furan derivatives with oxygen-bearing substituents, such as hydroxy, or alkoxy, at the benzene moiety are known to be biologically active compounds [24–28] (Figure 1).

Among the various approaches developed for the synthesis of the benzofuran ring system, the cyclodehydration of α-aryloxy ketones [29], the Claisen rearrangement of an allyl aryl ether followed by Pd-catalyzed intramolecular oxidative cyclization [30], and the tandem Sonogashira coupling/heterocyclization of 2-halophenols with terminal alkynes [31], are some of the most used. However, their application to the synthesis of 4-substituted benzo[*b*]furans is especially challenging, because the *meta*-substituted starting materials tend to cyclize at the less hindered *ortho*-position, leading to the formation of 6-substituted heterocycles or a mixture of 6- and 4-substituted ones [32].

**Scheme 1:**
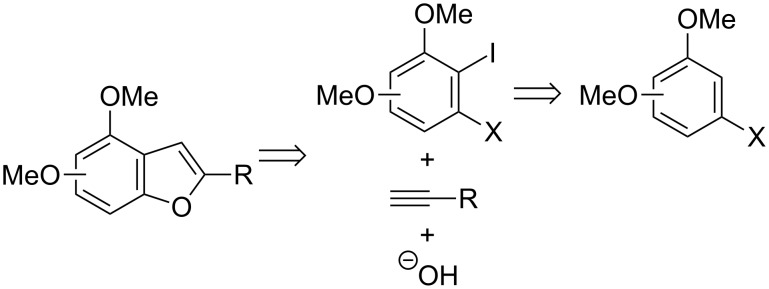
Retrosynthetic analysis of 4,n-dimethoxy-substituted benzo[*b*]furans.

The D*o*M strategy, when linked with different cross-coupling Pd-catalyzed reactions, could provide a superior approach for the construction of polysubstituted aromatic and heteroaromatic compounds [33–38]. In this context, we studied the *o*-lithiation of 3-halophenols and the resulting 2,3-difunctionalized phenol derivatives were applied to the synthesis of 4-functionalized benzo[*b*]furans [39], 4- or 7-alkoxyindoles [40], and 7-oxy-substituted benzo[*b*]thiophenes [41] by employing Pd-catalyzed cross-coupling reactions or halocyclization processes. Following our interest in the development of strategies for the synthesis of functionalized benzo[*b*]furan derivatives [39], we envisaged that 4,n-dimethoxybenzo[*b*]furans could be regioselectively synthesized from 3-halo-2-iodoanisoles bearing an additional methoxy group, by combining two palladium-catalyzed processes, that is a selective Sonogashira coupling and a tandem hydroxylation/heterocyclization reaction. The required *o*-dihaloanisole derivatives could be prepared by a selective *ortho*-metallation reaction and subsequent electrophilic quenching with iodine (Scheme 1).

## Results and Discussion

**Scheme 2:**
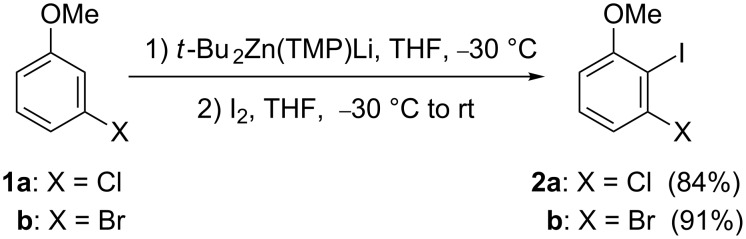
Deprotonative zincation of 3-haloanisoles **1**.

As established in our proposed retrosynthetic analysis (Scheme 1), we needed to develop a convenient synthesis of 3-halo-2-iodoanisoles bearing an additional methoxy group. Taking advantage of the deprotonative *ortho*-zincation of *meta*-functionalized haloaromatics by using TMP-zincates, described by Uchiyama and co-workers [11–12], we previously developed efficient syntheses of 3-chloro-2-iodoanisole (**2a**) and 3-bromo-2-iodoanisole (**2b**) from the corresponding 3-haloanisoles **1**, through their treatment with lithium di-*tert*-butyltetramethylpiperidinozincate, followed by electrophilic trapping of the intermediate arylzincate with iodine (Scheme 2) [40].

Thus, we decided to test this deprotonative metallation on the commercially available dimethoxyhalobenzene derivatives **3** with the same *t*-Bu_2_Zn(TMP)Li, easily prepared by reaction of preformed di-*tert*-butylzinc with lithium tetramethylpiperidide. Under these reaction conditions the zincation reactions took place regioselectively and, after treatment with iodine, afforded the corresponding 3-halo-2-iodoanisole derivatives **4** in good yields (Table 1). It is interesting to note that substrates **3c** and **3d** bearing the two methoxy groups in a *meta* relationship selectively undergo metalation at the position between the methoxy and the halide groups, irrespective of the nature of the halogen atom (chloro or bromo) (Table 1, entries 3 and 4).

**Table 1:** Synthesis of methoxy-substituted 3-halo-2-iodoanisoles **4**.

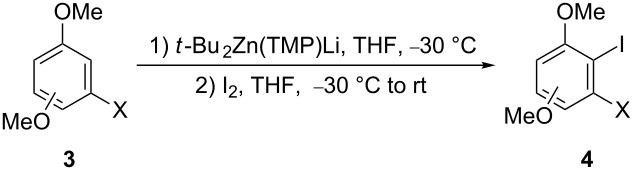					

Entry	Starting material	X	OMe^a^	Product	Yield (%)^b^

1	**3a**	Cl	*ortho*	**4a**	70
2	**3b**	Br	*ortho*	**4b**	73
3	**3c**	Cl	*meta*	**4c**	88
4	**3d**	Br	*meta*	**4d**	75
5	**3e**	Cl	*para*	**4e**	78
6	**3f**	Br	*para*	**4f**	80

^a^Position of the additional methoxy group relative to the existing one. ^b^Isolated yield based on the starting material **3**.

Having an efficient protocol for the synthesis of dimethoxyhaloiodobenzene derivatives **4**, and with the retrosynthetic analysis outlined in Scheme 1 in mind, we tackled the selective introduction of an alkynyl moiety at the iodinated position. The presence of two different halogen atoms in compounds **4** implies that a selective Sonogashira coupling reaction should occur (Table 2). This has been achieved in two different ways. Considering the steric hindrance of the required position (*o*,*o*-disubstituted) we employed a copper- and solvent-free methodology for the Sonogashira coupling that uses tetrabutylammonium fluoride as base [42] (Table 2, method A). Subsequently, we checked that the selective coupling could be carried out under standard Pd–Cu catalysis by controlling the reaction temperature [43–44] (Table 2, method B).

**Table 2:** Synthesis of dimethoxy-substituted *o*-alkynylhaloarenes **5**–**7**.

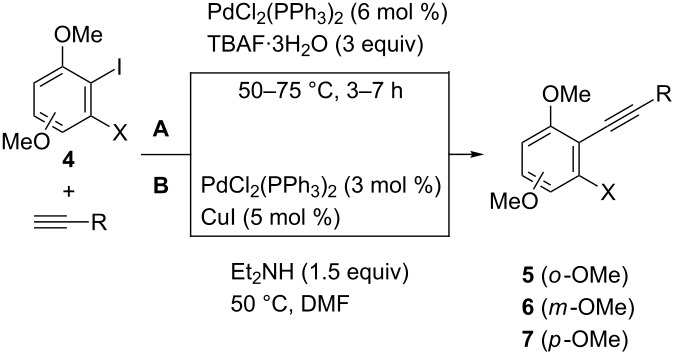							

Entry	Starting material	X	OMe^a^	R	Product	Method^b^	Yield (%)^c^

1	**4a**	Cl	*ortho*	*n*-Bu	**5a**	A	65
2	**4a**	Cl	*ortho*	*c*-C_6_H_9_^d^	**5b**	A	79
3	**4b**	Br	*ortho*	*n*-C_6_H_13_	**5c**	A	91
4	**4b**	Br	*ortho*	*n*-C_6_H_13_	**5c**	B	90
5	**4b**	Br	*ortho*	Ph	**5d**	A	83
6	**4b**	Br	*ortho*	Ph	**5d**	B	87
7	**4c**	Cl	*meta*	*n*-Bu	**6a**	A	57
8	**4c**	Cl	*meta*	*c*-C_6_H_9_^d^	**6b**	A	69
9	**4d**	Br	*meta*	*n*-C_5_H_11_	**6c**	A	63
10	**4d**	Br	*meta*	*n*-C_5_H_11_	**6c**	B	79
11	**4d**	Br	*meta*	Ph	**6d**	A	52
12	**4d**	Br	*meta*	Ph	**6d**	B	80
13	**4d**	Br	*meta*	4-MeC_6_H_4_	**6e**	A	75
14	**4e**	Cl	*para*	*n*-Bu	**7a**	A	69
15	**4f**	Br	*para*	*n*-C_5_H_11_	**7b**	A	55
16	**4f**	Br	*para*	*n*-C_5_H_11_	**7b**	B	95
17	**4f**	Br	*para*	Ph	**7c**	A	79
18	**4f**	Br	*para*	Ph	**7c**	B	92
19	**4f**	Br	*para*	3-FC_6_H_4_	**7d**	A	93

^a^Position of the additional methoxy group referred to the existing one. ^b^Method A: alkyne (1.5–2 equiv), PdCl_2_(PPh_3_)_2_ (6 mol %), TBAF·3H_2_O (3 equiv), 50–60 °C. Method B: alkyne (1.2 equiv), PdCl_2_(PPh_3_)_2_ (3 mol %), CuI (5 mol %), Et_2_NH (1.5 equiv), DMF, 50 °C. ^c^Isolated yield based on the starting material **4**. ^d^1-Cyclohexenyl.

By either of these procedures, A or B, *o*-alkynylhaloarenes **5**–**7** were prepared usually in high yields from the corresponding dimethoxyhaloiodobenzenes **4** (Table 2). Starting from chloroiodo derivatives **4a,c** and **e** good yields were obtained for the corresponding coupled products **5**–**7** by using the Pd/TBAF method A, with no significant side-products (Table 2, entries 1,2,7,8 and 14). However, under these conditions bromine-containing compounds **4b,d** and **f** afforded variable amounts of dialkynylation products in some cases, and so the Pd/Cu catalyzed procedure B gave rise to better selectivities and yields of the desired alkynes **5**–**7** (Table 2, entries 4,6,10,12,16 and 18).

According to our retrosynthetic analysis (Scheme 1), the final step to achieve the benzofuran derivatives should be the incorporation of the hydroxy group followed by in situ heterocyclization. In recent years, the direct hydroxylation of aryl halides has been developed by several groups by using palladium- or copper-catalyzed protocols. Whereas the reactions under copper catalysis work well for aryl iodides [45–48], the palladium-catalyzed hydroxylation also takes place with aryl bromides and chlorides [49–51]. Thus, in our case we employed the Pd-catalyzed Buchwald protocol [49] in an attempt to synthesise the desired dimethoxybenzo[*b*]furan derivatives. Thus, reaction of the previously prepared *o*-haloaryl alkynes **5**–**7** with KOH, in the presence of catalytic amounts of Pd_2_(dba)_3_ and *t*-BuXPhos (2-di-*tert*-butylphosphino-2',4',6'-triisopropylbiphenyl) at 100 °C in a 1:1 mixture of H_2_O:1,4-dioxane, gave rise to the corresponding benzo[*b*]furan derivatives **8**–**10** in moderate to good yields (Table 3, method C). In general, slightly better results were obtained starting from aryl bromides instead of aryl chlorides. In addition, we also observed better yields for the corresponding benzofuran derivatives **9** (Table 3, entries 5–9) derived from the starting *o*-alkynylhalobenzene derivatives **6**, bearing the two methoxy groups in a 3,5-relationship relative to the halide. Moreover, because of the extended reaction times needed for complete consumption of the starting materials, we developed an alternative protocol under microwave irradiation that shortens the time required for the coupling to a few minutes (method D), and the final products were obtained in similar yields to those obtained by the conventional coupling method C.

**Table 3:** Synthesis of dimethoxy-substituted benzo[*b*]furans **8**–**10**.

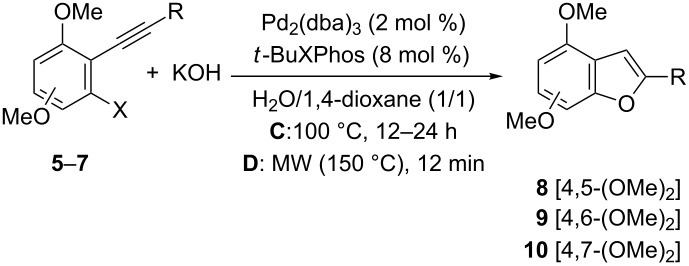							

Entry	Starting material	X	R	Product	OMe^a^	Method^b^	Yield (%)

1	**5a**	Cl	*n*-Bu	**8a**	4,5-(MeO)_2_	D	55
2	**5b**	Cl	*c*-C_6_H_9_^c^	**8b**	4,5-(MeO)_2_	C	57
3	**5c**	Br	*n*-C_6_H_13_	**8c**	4,5-(MeO)_2_	D	55
4	**5d**	Br	Ph	**8d**	4,5-(MeO)_2_	C	50
5	**6a**	Cl	*n*-Bu	**9a**	4,6-(MeO)_2_	C	62
6	**6b**	Cl	*c*-C_6_H_9_^c^	**9b**	4,6-(MeO)_2_	C	73
7	**6c**	Br	*n*-C_5_H_11_	**9c**	4,6-(MeO)_2_	C	70
8	**6d**	Br	Ph	**9d**	4,6-(MeO)_2_	C	81
9	**6e**	Br	4-MeC_6_H_4_	**9e**	4,6-(MeO)_2_	C	75
10	**7a**	Cl	*n*-Bu	**10a**	4,7-(MeO)_2_	C	60
11	**7b**	Br	*n*-C_5_H_11_	**10b**	4,7-(MeO)_2_	C	64
12	**7c**	Br	Ph	**10c**	4,7-(MeO)_2_	D	71
13	**7d**	Br	3-FC_6_H_4_	**10d**	4,7-(MeO)_2_	D	65

^a^Position of the methoxy groups referred to benzo[*b*]furan moiety. ^b^Method C: conventional heating (100 °C, overnight). Method D: MW (50 W, 150 °C, 12 min). ^c^1-Cyclohexenyl.

## Conclusion

We have developed a synthetic route to access regioselectively functionalized 4,n-dimethoxybenzo[*b*]furans through combined directed *ortho*-metallation (DoM)/palladium-catalyzed reactions. The deprotonative *ortho*-zincation of *meta*-functionalized haloanisoles bearing an additional methoxy group, followed by electrophilic quenching with iodine allows the regioselective and straightforward synthesis of highly functionalized dihalodimethoxybenzene derivatives. A subsequent selective Sonogashira coupling with terminal alkynes, followed by direct hydroxylation with KOH of the resulting *o*-haloaryl alkyne and in situ heterocyclization, afforded the benzo[*b*]furan derivatives. In addition, we have developed a new procedure for the Pd-catalyzed hydroxylation reaction that allows the coupling to take place within in a few minutes.

## Supporting Information

Experimental procedures and spectroscopic data for all new compounds. Copies of ^1^H NMR and ^13^C NMR spectra for new compounds.

File 1 Experimental and analytical data.

File 2 NMR spectra.
