# Supplementary material for: Combined directed ortho-zincation and palladium-catalyzed strategies: Synthesis of 4,n-dimethoxy-substituted benzo[b]furans
Source: Beilstein J Org Chem. 2011 Sep 12;7:1255–60. doi: 10.3762/bjoc.7.146 (PMC3182435; doi:10.3762/bjoc.7.146)
Supplement: File 1 — Experimental and analytical data. [file Beilstein_J_Org_Chem-07-1255-s001.pdf]

Supporting Information File 1

for

Combined directed *ortho*-zincation and palladium-catalyzed strategies: Synthesis of 4,n-dimethoxy-substituted benzo[*b*]furans

Verónica Guilarte, M. Pilar Castroviejo, Estela Álvarez and Roberto Sanz*

Address: Área de Química Orgánica, Departamento de Química, Facultad de Ciencias, Universidad de Burgos, Pza. Misael Bañuelos s/n, 09001 Burgos, Spain

Email: Roberto Sanz* - rsd@ubu.es

* Corresponding author

Experimental and analytical data

Materials and Methods

All reactions involving air-sensitive compounds were carried out under a N_2 atmosphere, in oven-dried glassware, with magnetic stirring. The microwave heating was performed in a Microwave, CEM Discover S-Class single-mode microwave cavity producing continuous irradiation (Temperature measurements were conducted using an IR sensor located below the microwave cavity floor, and reaction times refer to the total hold time at the indicated temperature. The maximum wattage supplied was 300 W). Temperatures are reported as bath temperatures. Solvents used in extraction and purification were distilled prior to use. TLC was performed on alumina-backed plates coated with silica gel 60 with F_{254} indicator; the chromatograms were visualized by UV light (254 nm) and/or by staining with a Ce/Mo reagent, anisaldehyde or phosphomolybdic acid solution and subsequent heating. R_f values refer to silica gel. Flash column chromatography was carried out on silica gel 60, 230-400 mesh. Melting points were obtained with open capillary tubes and are uncorrected. ^1H NMR spectra were recorded at 400 or 300 MHz. Chemical shifts are reported in ppm with the residual solvent resonance as the internal standard ($CHCl_3$: δ 7.26). Data are reported as follows: chemical shift, multiplicity (s: singlet, br s: broad singlet, d: doublet, dd: doublet of doublets, dt: doublet of triplets, ddd: doublet of doublet of doublets, t: triplet, appt: apparent triplet, td: triplet of doublets, tdd: triplet of doublet of doublets, q: quartet, m: multiplet), coupling constants (*J* in Hz) and integration. ^{13}C NMR spectra were recorded at 100.6 or 75.4 MHz using broadband proton decoupling. Chemical shifts are reported in ppm with the solvent resonance as internal standard ($CDCl_3$: δ 77.16). Low-resolution electron impact mass spectra (EI-LRMS) were obtained at 70 eV on a mass spectrometer and only the molecular ions and/or base peaks as well as significant peaks in MS are given. High-resolution mass spectrometry (HRMS) was carried out on a mass spectrometer. Infrared spectra were

recorded with a FT-IR spectrophotometer. Elemental analyses were performed on a microanalyzer. All commercially available reagents were used without purification unless otherwise indicated and were purchased from standard chemical suppliers.

General procedure 1 (GP1) for the synthesis of 3-halo-2-iodoanisole derivatives 2 and 4 according to [1]: To a solution of lithium 2,2,6,6-tetramethylpiperidide (20 mmol, generated from *n*-BuLi and 2,2,6,6-tetramethylpiperidine) in anhydrous THF (30 mL), a solution of *t*-Bu_2Zn (22 mmol, generated from *t*-BuLi and dry $ZnCl_2$) in anhydrous THF (30 mL) was added at –78 °C, and the reaction mixture was stirred at 0 °C for 30 min. Then the corresponding 3-haloanisole derivative **1** or **3** (10 mmol) was added at –78 °C, and the reaction mixture was allowed to reach –30 °C and was stirred at this temperature overnight. Iodine (17.78 g, 70 mmol) in THF (30 mL) was added to the reaction mixture at –30 °C, and after 30 min at this temperature the resulting mixture was allowed to reach room temperature and stirred for 2 h. The reaction was quenched with saturated $Na_2S_2O_3$, and the aqueous solution was extracted with Et_2O (3 × 30 mL). The combined organic layers were dried over anhydrous Na_2SO_4 and evaporated under reduced pressure. The crude product was subjected to column chromatography on silica gel (eluent: hexane/EtOAc, 20/1).
For data of compounds **2a** and **2b** see [1].

1-Chloro-2-iodo-3,4-dimethoxybenzene (4a)

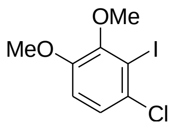

Reaction of 4-chloro-1,2-dimethoxybenzene (**3a**) (1.73 g, 10 mmol) following **GP1** afforded **4a** as a colourless oil (2.09 g, 70%). R_f 0.15 (hexane/EtOAc, 20/1). ^{1}H NMR (300 MHz, $CDCl_3$): δ 3.81 (s, 3H), 3.84 (s, 3H), 6.82 (d, J = 8.8 Hz, 1H), 7.15 (d, J =

8.8 Hz, 1H) ppm. ^{13}C NMR (75.4 MHz, $CDCl_3$) δ 56.6 (CH_3), 60.4 (CH_3), 97.9 (C), 113.1 (CH), 124.5 (CH), 130.0 (C), 150.3 (C), 151.0 (C) ppm. LRMS (70 eV, EI): *m/z* (%) 300 (M^++2, 33), 298 (M^+, 100), 283 (39), 128 (19). HRMS (EI) calcd for $C_8H_8ClIO_2$, 297.9258; found, 297.9258.

1-Bromo-2-iodo-3,4-dimethoxybenzene (4b)

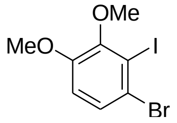

Reaction of 4-bromo-1,2-dimethoxybenzene (**3b**) (2.17 g, 10 mmol) following **GP1** afforded **4b** as a white solid (2.50 g, 73%). Mp 44–46 °C. ^{1}H NMR (300 MHz, $CDCl_3$): δ 3.80 (s, 3H), 3.83 (s, 3H), 6.78 (d, *J* = 8.8 Hz, 1H), 7.32 (d, *J* = 8.8 Hz, 1H) ppm. ^{13}C NMR (75.4 MHz, $CDCl_3$) δ 56.2 (CH_3), 60.4 (CH_3), 100.9 (C), 113.7 (CH), 120.2 (C), 127.8 (CH), 150.6 (C), 151.4 (C) ppm. LRMS (70 eV, EI): *m/z* (%) 344 (M^++2, 98%), 342 (M^+, 100), 329 (29), 327 (31), 172 (22). HRMS (EI) calcd for $C_8H_8BrIO_2$, 341.8752; found, 341.8754.

1-Chloro-2-iodo-3,5-dimethoxybenzene (4c)

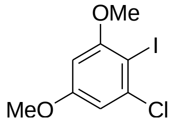

Reaction of 5-chloro-1,3-dimethoxybenzene (**3c**) (1.73 g, 10 mmol) following **GP1** afforded **4c** as a white solid (2.63 g, 88%). Mp 80–82 °C. ^{1}H NMR (300 MHz, $CDCl_3$): δ 3.78 (s, 3H), 3.84 (s, 3H), 6.29 (d, *J* = 2.7 Hz, 1H), 6.67 (d, *J* = 2.7 Hz, 1H) ppm. ^{13}C NMR (75.4 MHz, $CDCl_3$) δ 55.8 (CH_3), 56.8 (CH_3), 80.3 (C), 97.3 (CH), 106.5 (CH), 139.8 (C), 160.2 (C), 161.2 (C) ppm. LRMS (70 eV, EI): *m/z* (%) 300 (M^++2, 33), 298 (M^+, 100), 156 (14), 141 (14). HRMS (EI) calcd for $C_8H_8ClIO_2$, 297.9258; found, 297.9256.

1-Bromo-2-iodo-3,5-dimethoxybenzene (4d)

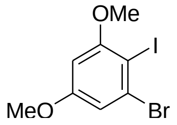

Reaction of 1-bromo-3,5-dimethoxybenzene (**3d**) (2.17 g, 10 mmol) following **GP1** afforded **4d** as a white solid (2.57 g, 75%). Mp 95–97 °C. ^{1}H NMR (300 MHz, $CDCl_3$): δ 3.78 (s, 3H), 3.83 (s, 3H), 6.32 (d, *J* = 2.6 Hz, 1H), 6.85 (d, *J* = 2.6 Hz, 1H) ppm. ^{13}C NMR (75.4 MHz, $CDCl_3$) δ 55.8 (CH_3), 56.8 (CH_3), 83.5 (C), 97.8 (CH), 109.7 (CH), 131.0 (C), 160.4 (C), 161.3 (C) ppm. LRMS (70 eV, EI): *m/z* (%) 344 (M^++2, 100), 342 (M^+, 100), 301 (10), 299 (11), 187 (16), 185 (17). HRMS (EI) calcd for $C_8H_8BrIO_2$, 341.8752; found, 341.8758.

2-Chloro-3-iodo-1,4-dimethoxybenzene (4e)

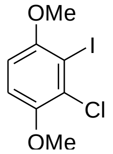

Reaction of 3-chloro-1,4-dimethoxybenzene (**3e**) (1.73 g, 10 mmol) following **GP1** afforded **4e** as a white solid (2.33 g, 78%). Mp 129–131 °C. ^{1}H NMR (400 MHz, $CDCl_3$): δ 3.81 (s, 3H), 3.82 (s, 3H), 6.67 (1H, d, *J* = 9.2 Hz), 6.86 (1H, d, *J* = 9.2 Hz) ppm. ^{13}C NMR (100.6 MHz, $CDCl_3$) δ 57.0 (CH_3), 57.1 (CH_3), 93.3 (C), 109.0 (CH), 112.1 (CH), 128.3 (C), 149.9 (C), 153.7 (C) ppm. LRMS (70 eV, EI): *m/z* (%) 300 (M^++2, 33), 298 (M^+, 100), 285 (25), 283 (79). HRMS (EI) calcd for $C_8H_8ClIO_2$, 297.9258; found, 297.9254.

2-Bromo-3-iodo-1,4-dimethoxybenzene (4f)

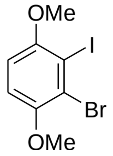

Reaction of 1-bromo-2,5-dimethoxybenzene (**3f**) (2.17 g, 10 mmol) following **GP1** afforded **4f** as a white solid (2.74 g, 80%). Mp 153–155 °C. ^{1}H NMR (300 MHz, $CDCl_3$): δ 3.83 (3H, s), 3.84 (3H, s), 6.77 (1H, d, *J* = 8.8 Hz), 6.89 (1H, d, *J* = 8.8 Hz)

ppm. ^{13}C NMR (75.4 MHz, $CDCl_3$) δ 57.2 (CH_3), 57.3 (CH_3), 96.6 (C), 109.9 (CH), 112.1 (CH), 121.0 (C), 151.3 (C), 154.1 (C) ppm. LRMS (70 eV, EI): *m/z* (%) 344 (M^++2, 98), 342 (M^+, 100), 329 (64), 327 (65). HRMS (EI) calcd for $C_8H_8BrIO_2$, 341.8752; found, 341.8750.

General procedure 2 (GP2) for the copper-free Sonogashira coupling of 3-halo-2-iodoanisoles 4 with terminal alkynes (Method A). Synthesis of *o*-alkynylhaloarenes 5–7 [2]: A mixture of the corresponding iodobenzene derivative **4** (2.5 mmol), the terminal alkyne (1.5–2 equiv), $PdCl_2(PPh_3)_2$ (105 mg, 0.15 mmol) and TBAF·$3H_2O$ (2.37 g, 7.5 mmol) was stirred under nitrogen at 50–60 °C until complete consumption of starting material (as judged by GC-MS, 2–4 h). The mixture is allowed to reach room temperature, quenched with water, and extracted with Et_2O (3 × 15 mL). The combined organic layers were dried over anhydrous Na_2SO_4 and evaporated under reduced pressure. The crude product was purified by column chromatography on silica gel (eluent: hexane/EtOAc).

General procedure 3 (GP3) for the Pd/Cu Sonogashira coupling of 3-halo-2-iodoanisoles 4 with terminal alkynes (Method B). Synthesis of *o*-alkynylhaloarenes 5–7: A mixture of the corresponding iodobenzene derivative **4** (1 equiv), the terminal alkyne (1.2 equiv), $PdCl_2(PPh_3)_2$ (3 mol %), CuI (5 mol %) and Et_2NH (1.5 equiv) in anhydrous DMF (4 mL/mmol) was stirred under nitrogen at 50 °C until complete consumption of starting material (as judged by GC-MS, 2–4 h). CH_2Cl_2 (20 mL/mmol) and 0.5 M HCl (20 mL/mmol) were added to the cooled reaction mixture. The separated aqueous phase was extracted with CH_2Cl_2 (2 ×

20 mL). The combined organic layers were washed with water (2 × 40 mL). The organic layer was dried over anhydrous Na_2SO_4 and concentrated at reduced pressure. The remaining residue was purified by column chromatography on silica gel (eluent: hexane/EtOAc).

1-Chloro-2-hex-1-ynyl-3,4-dimethoxybenzene (5a)

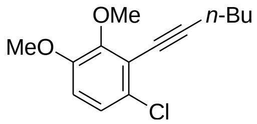

Reaction of **4a** (746 mg, 2.5 mmol) with 1-hexyne (411 mg, 5 mmol) for 4 h following **GP2**, and purification by column chromatography (hexane/EtOAc, 20/1), afforded **5a** (411 mg, 65%) as a brown oil. R_f 0.18 (hexane/EtOAc, 20/1). ^{1}H NMR (300 MHz, $CDCl_3$): δ 0.95 (t, *J* = 7.2 Hz, 3H), 1.46–1.70 (m, 4H), 2.53 (t, *J* = 6.9 Hz, 2H), 3.83 (s, 3H), 3.90 (s, 3H), 6.75 (d, *J* = 8.9 Hz, 1H), 7.06 (d, *J* = 8.9 Hz, 1H) ppm. ^{13}C NMR (75.4 MHz, $CDCl_3$) δ 13.8 (CH_3), 19.7 (CH_2), 22.0 (CH_2), 30.8 (CH_2), 56.3 (CH_3), 61.0 (CH_3), 73.7 (C), 100.6 (C), 112.3 (CH), 119.3 (C), 124.3 (CH), 127.8 (C), 151.3 (C), 151.5 (C) ppm. LRMS (70 eV, EI): *m/z* (%) 254 (M^++2, 28), 252 (M^+, 88), 195 (100), 131 (51), 115 (98), 103 (63). HRMS (EI) calcd for $C_{14}H_{17}ClO_2$, 252.0917; found, 252.0923.

1-Chloro-2-cyclohex-1-enylethynyl-3,4-dimethoxybenzene (5b)

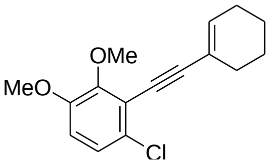

Reaction of **4a** (746 mg, 2.5 mmol) with 1-ethynylcyclohexene (531 mg, 5 mmol) for 4 h following **GP2**, and purification by column chromatography (hexane/EtOAc, 20/1), afforded **5b** (546 mg, 79%) as a brown oil. R_f 0.22 (hexane/EtOAc, 20/1). ^{1}H NMR (300 MHz, $CDCl_3$): δ 1.54–1.71 (m, 4H), 2.09–2.17 (m, 2H), 2.21–2.29 (m, 2H), 3.79 (s, 3H), 3.89 (s, 3H), 6.26 (tt, *J* = 4.0, 1.8 Hz, 1H), 6.73 (d, *J* = 8.9 Hz, 1H), 7.02 (d, *J*

= 8.9 Hz, 1H) ppm. ^{13}C NMR (75.4 MHz, $CDCl_3$) δ 21.5 (CH_2), 22.3 (CH_2), 25.8 (CH_2), 29.0 (CH_2), 56.0 (CH_3), 60.9 (CH_3), 79.9 (C), 100.8 (C), 112.4 (CH), 118.8 (C), 120.7 (C), 124.1 (CH), 127.4 (C), 136.0 (CH), 150.8 (C), 151.4 (C) ppm. LRMS (70 eV, EI): *m/z* (%) 278 (M^++2, 34), 276 (M^+, 100), 261 (31), 195 (54), 165 (61), 152 (65), 115 (85), 87 (77), 77 (88). HRMS (EI) calcd for $C_{16}H_{17}ClO_2$, 276.0917; found, 276.0911.

1-Bromo-3,4-dimethoxy-2-(oct-1-ynyl)benzene (5c)

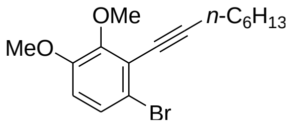

Reaction of **4b** (857 mg, 2.5 mmol) with 1-octyne (413 mg, 3.75 mmol) for 2 h following **GP2**, and purification by column chromatography (hexane/EtOAc, 40/1), afforded **5c** (739 mg, 91%) as a pale brown oil. R_f 0.57 (hexane/EtOAc, 7/1). ^{1}H NMR (300 MHz, $CDCl_3$): δ 0.87 (t, J = 6.9 Hz, 3H), 1.25–1.33 (m, 4H), 1.44–1.54 (m, 2H), 1.57–1.68 (m, 2H), 2.49 (t, J = 6.9 Hz, 2H), 3.78 (s, 3H), 3.86 (s, 3H), 6.66 (d, J = 8.9 Hz, 1H), 7.19 (d, J = 8.9 Hz, 1H) ppm. ^{13}C NMR (75.4 MHz, $CDCl_3$) δ 14.1 (CH_3), 19.8 (CH_2), 22.6 (CH_2), 28.5 (2 $\times$ CH_2), 31.3 (CH_2), 55.9 (CH_3), 60.7 (CH_3), 75.4 (C), 99.8 (C), 112.6 (CH), 116.3 (C), 121.1 (C), 127.2 (CH), 151.3 (C), 151.9 (C) ppm. LRMS (70 eV, EI): *m/z* (%) 326 (M^++2, 58), 324 (M^+, 60), 241 (32), 239 (34), 216 (24), 176 (100). HRMS (EI) calcd for $C_{16}H_{21}BrO_2$, 324.0725; found, 324.0721.

1-Bromo-3,4-dimethoxy-2-(phenylethynyl)benzene (5d)

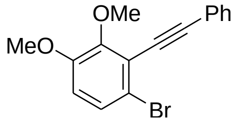

Reaction of **4b** (686 mg, 2 mmol) with phenylacetylene (245 mg, 2.4 mmol) for 3 h following **GP3**, and purification by column chromatography (hexane/EtOAc, 30/1), afforded **5d** (552 mg, 87%) as a brown oil. R_f 0.40 (hexane/EtOAc, 9/1). ^{1}H NMR (300 MHz, $CDCl_3$): δ 3.84 (s, 3H), 3.99 (s, 3H), 6.76 (d, J = 8.8 Hz, 1H), 7.29 (d, J =

8.8 Hz, 1H), 7.34–7.39 (m, 3H), 7.60 (d, J = 2.3 Hz, 1H), 7.58–7.64 (m, 1H) ppm. ^{13}C NMR (75.4 MHz, $CDCl_3$) δ 56.2 (CH_3), 61.2 (CH_3), 84.4 (C), 98.0 (C), 113.5 (CH), 116.4 (C), 120.5 (C), 123.1 (C), 127.5 (CH), 128.4 (2 × CH), 128.7 (CH), 131.7 (2 × CH), 151.3 (C), 152.1 (C) ppm. LRMS (70 eV, EI): *m/z* (%) 318 (M^++2, 98), 316 (M^+, 100), 179 (45), 165 (43), 150 (30). HRMS (EI) calcd for $C_{16}H_{13}BrO_2$, 316.0099; found, 316.0093.

1-Chloro-2-hex-1-ynyl-3,5-dimethoxybenzene (6a)

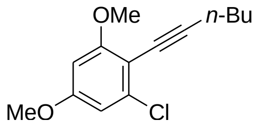

Reaction of **4c** (746 mg, 2.5 mmol) with 1-hexyne (411 mg, 5 mmol) for 4 h following **GP2**, and purification by column chromatography (hexane/EtOAc, 20/1), afforded **5a** (360 mg, 57%) as a brown oil. R_f 0.23 (hexane/EtOAc, 9/1). ^{1}H NMR (300 MHz, $CDCl_3$): δ 0.92 (t, J = 7.2 Hz, 3H), 1.44–1.64 (m, 4H), 2.49 (t, J = 6.9 Hz, 2H), 3.80 (s, 3H), 3.84 (s, 3H), 6.28 (d, J = 2.3 Hz, 1H), 6.50 (d, J = 2.3 Hz, 1H) ppm. ^{13}C NMR (75.4 MHz, $CDCl_3$) δ 13.7 (CH_3), 19.6 (CH_2), 22.0 (CH_2), 30.9 (CH_2), 55.5 (CH_3), 56.1 (CH_3), 73.4 (C), 97.0 (CH), 98.5 (C), 105.8 (CH), 106.3 (C), 137.7 (C), 159.7 (C), 161.6 (C) ppm. LRMS (70 eV, EI): *m/z* (%) 254 (M^++2, 28), 252 (M^+, 100), 237 (35), 195 (41), 159 (33). HRMS (EI) calcd for $C_{14}H_{17}ClO_2$, 252.0917; found, 252.0918.

1-Chloro-2-cyclohex-1-enylethynyl-3,5-dimethoxybenzene (6b)

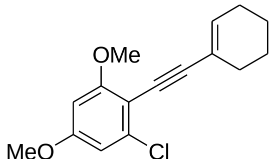

Reaction of **4c** (746 mg, 2.5 mmol) with 1-ethynylcyclohexene (531 mg, 5 mmol) for 3 h following **GP2**, and purification by column chromatography (hexane/EtOAc, 20/1), afforded **6b** (477 mg, 69%) as a yellow solid. Mp 92–94 °C. ^{1}H NMR (300 MHz, $CDCl_3$): δ 1.54–1.72 (m, 4H), 2.08–2.16 (m, 2H), 2.22–2.30 (m, 2H), 3.76 (s, 3H),

3.82 (s, 3H), 6.18–6.25 (m, 1H), 6.30 (d, *J* = 2.3 Hz, 1H), 6.52 (d, *J* = 2.3 Hz, 1H) ppm. ^{13}C NMR (75.4 MHz, $CDCl_3$) δ 21.6 (CH_2), 22.4 (CH_2), 25.8 (CH_2), 29.3 (CH_2), 55.6 (CH_3), 56.1 (CH_3), 79.8 (C), 97.0 (CH), 99.0 (C), 105.9 (CH), 106.1 (C), 120.9 (C), 135.0 (CH), 137.6 (C), 160.0 (C), 161.4 (C) ppm. LRMS (70 eV, EI): *m/z* (%) 278 (M^++2, 33), 276 (M^+, 100), 248 (17), 195 (15). HRMS (EI) calcd for $C_{16}H_{17}ClO_2$, 276.0917; found, 276.0920.

1-Bromo-2-hept-1-ynyl-3,5-dimethoxybenzene (6c)

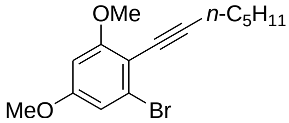

Reaction of **4d** (514 mg, 1.5 mmol) with 1-heptyne (173 mg, 1.8 mmol) for 3 h following **GP3**, and purification by column chromatography (hexane/EtOAc, 30/1), afforded **6c** (369 mg, 79%) as a white solid. Mp 42–44 °C. ^{1}H NMR (300 MHz, $CDCl_3$): δ 0.91 (t, *J* = 7.2 Hz, 3H), 1.30–1.42 (m, 2H), 1.44–1.54 (m, 2H), 1.60–1.70 (m, 2H), 2.50 (t, *J* = 7.0 Hz, 2H), 3.78 (s, 3H), 3.83 (s, 3H), 6.36 (d, *J* = 2.3 Hz, 1H), 6.72 (d, *J* = 2.3 Hz, 1H) ppm. ^{13}C NMR (75.4 MHz, $CDCl_3$) δ 14.2 (CH_3), 20.0 (CH_2), 22.4 (CH_2), 28.6 (CH_2), 31.2 (CH_2), 55.7 (CH_3), 56.3 (CH_3), 75.4 (C), 97.8 (CH), 98.2 (C), 108.6 (C), 109.0 (CH), 127.5 (C), 159.9 (C), 161.8 (C) ppm. LRMS (70 eV, EI): *m/z* (%) 312 (M^++2, 84), 310 (M^+, 84), 283 (34), 281 (36), 257 (52), 255 (75), 231 (52), 229 (41), 176 (100), 159 (99). HRMS (EI) calcd for $C_{15}H_{19}BrO_2$, 310.0568; found, 310.0571.

1-Bromo-3,5-dimethoxy-2-(phenylethynyl)benzene (6d)

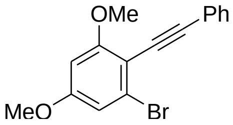

Reaction of **4d** (686 mg, 2 mmol) with phenylacetylene (245 mg, 2.4 mmol) for 3 h following **GP3**, and purification by column chromatography (hexane/EtOAc, 30/1), afforded **6d** (507 mg, 80%) as a brown solid. Mp 93–95 °C. ^{1}H NMR (300 MHz,

$CDCl_3$): δ 3.81 (s, 3H), 3.88 (s, 3H), 6.40 (d, *J* = 2.3 Hz, 1H), 6.78 (d, *J* = 2.3 Hz, 1H), 7.30–7.39 (m, 3H), 7.57–7.63 (m, 2H) ppm. ^{13}C NMR (75.4 MHz, $CDCl_3$) δ 55.8 (CH_3), 56.3 (CH_3), 84.7 (C), 96.6 (C), 97.8 (CH), 107.9 (C), 109.2 (CH), 123.7 (C), 127.7 (C), 128.2 (CH), 128.3 (2 × CH), 131.6 (2 × CH), 160.7 (C), 161.8 (C) ppm. LRMS (70 eV, EI): *m/z* (%) 318 (M^++2, 98), 316 (M^+, 100), 179 (92), 165 (99), 150 (79). HRMS (EI) calcd for $C_{16}H_{13}BrO_2$, 316.0099; found, 316.0099.

1-Bromo-3,5-dimethoxy-2-*p*-tolylethynylbenzene (6e)

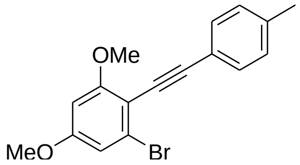

Reaction of **4d** (857 mg, 2.5 mmol) with 4-ethynyltoluene (436 mg, 3.75 mmol) for 3.5 h following **GP2**, and purification by column chromatography (hexane/EtOAc, 30/1), afforded **6e** (621 mg, 75%) as a brown solid. Mp 104–106 °C. ^1H NMR (300 MHz, $CDCl_3$): δ 2.37 (s, 3H), 3.79 (s, 3H), 3.86 (s, 3H), 6.39 (d, *J* = 2.2 Hz, 1H), 6.78 (d, *J* = 2.2 Hz, 1H), 7.16 (d, *J* = 8.0 Hz, 2H), 7.51 (d, *J* = 8.0 Hz, 2H) ppm. ^{13}C NMR (75.4 MHz, $CDCl_3$) δ 21.5 (CH_3), 55.6 (CH_3), 56.1 (CH_3), 84.0 (C), 96.7 (C), 97.6 (CH), 108.0 (C), 109.1 (CH), 120.5 (C), 127.4 (C), 129.0 (2 × CH), 131.4 (2 × CH), 138.2 (C), 160.5 (C), 161.6 (C) ppm. LRMS (70 eV, EI): *m/z* (%) 332 (M^++2, 100), 330 (M^+, 99), 317 (12), 315 (15), 207 (65), 193 (38). HRMS (EI) calcd for $C_{17}H_{15}BrO_2$, 330.0255; found, 330.0266.

2-Chloro-3-hex-1-ynyl-1,4-dimethoxybenzene (7a)

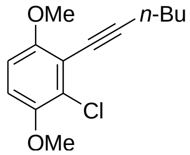

Reaction of **4e** (746 mg, 2.5 mmol) with 1-hexyne (411 mg, 5 mmol) for 5 h following **GP2**, and purification by column chromatography (hexane/EtOAc, 20/1), afforded **7a** (436 mg, 69%) as a brown oil. R_f 0.12 (hexane/EtOAc, 20/1). ^1H NMR (300 MHz,

CDCl$_3$): δ 0.93 (t, *J* = 7.2 Hz, 3H), 1.44–1.69 (m, 4H), 2.52 (t, *J* = 7.2 Hz, 2H), 3.80 (s, 3H), 3.81 (s, 3H), 6.67 (d, *J* = 9.1 Hz, 1H), 6.77 (d, *J* = 9.1 Hz, 1H) ppm. ^{13}C NMR (75.4 MHz, CDCl$_3$) δ 13.7 (CH$_3$), 19.7 (CH$_2$), 22.0 (CH$_2$), 30.8 (CH$_2$), 56.5 (CH$_3$), 56.7 (CH$_3$), 73.7 (C), 101.0 (C), 109.0 (CH), 111.4 (CH), 114.9 (C), 125.8 (C), 149.5 (C), 154.9 (C) ppm. LRMS (70 eV, EI): *m/z* (%) 254 (M$^+$+2, 35), 252 (M$^+$, 100), 195 (41). HRMS (EI) calcd for $C_{14}H_{17}ClO_2$, 252.0917; found, 252.0925.

2-Bromo-3-hept-1-ynyl-1,4-dimethoxybenzene (7b)

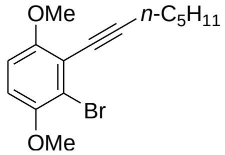

Reaction of **4f** (514 mg, 1.5 mmol) with 1-heptyne (173 mg, 1.8 mmol) for 4 h following **GP3**, and purification by column chromatography (hexane/EtOAc, 25/1), afforded **7b** (443 mg, 95%) as a brown oil. R_f 0.38 (hexane/EtOAc, 9/1). ^{1}H NMR (300 MHz, CDCl$_3$): δ 0.91 (t, *J* = 7.2 Hz, 3H), 1.30–1.42 (m, 2H), 1.44–1.55 (m, 2H), 1.61–1.72 (m, 2H), 2.53 (t, *J* = 7.0 Hz, 2H), 3.82 (s, 3H), 3.83 (s, 3H), 6.78–6.73 (m, 2H) ppm. ^{13}C NMR (75.4 MHz, CDCl$_3$) δ 14.2 (CH$_3$), 20.1 (CH$_2$), 22.3 (CH$_2$), 28.4 (CH$_2$), 31.2 (CH$_2$), 56.7 (CH$_3$), 56.9 (CH$_3$), 75.8 (C), 100.6 (C), 109.9 (CH), 111.4 (CH), 116.8 (C), 117.2 (C), 150.6 (C), 155.2 (C) ppm. LRMS (70 eV, EI): *m/z* (%) 312 (M$^+$+2, 87), 310 (M$^+$, 86), 241 (33), 239 (36), 202 (33), 176 (100). HRMS (EI) calcd for $C_{15}H_{19}BrO_2$, 310.0568; found, 310.0566.

2-Bromo-1,4-dimethoxy-3-(phenylethynyl)benzene (7c)

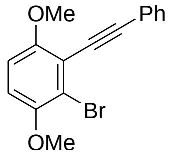

Reaction of **4f** (686 mg, 2 mmol) with phenylacetylene (245 mg, 2.4 mmol) for 3 h following **GP3**, and purification by column chromatography (hexane/EtOAc, 25/1), afforded **7c** (584 mg, 92%) as a brown solid. Mp 66–68 °C. ^{1}H NMR (300 MHz,

$CDCl_3$): δ 3.83 (s, 3H), 3.85 (s, 3H), 6.74–6.84 (m, 2H), 7.32–7.40 (m, 3H), 7.59–7.69 (m, 2H) ppm. ^{13}C NMR (75.4 MHz, $CDCl_3$) δ 56.7 (CH_3), 57.0 (CH_3), 84.8 (C), 98.5 (C), 110.1 (CH), 112.3 (CH), 116.5 (C), 116.7 (C), 123.3 (C), 128.4 (2 × CH), 128.6 (CH), 131.9 (2 × CH), 150.7 (C), 155.2 (C) ppm. LRMS (70 eV, EI): *m/z* (%) 318 (M^++2, 98), 316 (M^+, 100), 303 (30), 301 (32), 179 (28), 165 (29), 151 (22). HRMS (EI) calcd for $C_{16}H_{13}BrO_2$, 316.0099; found, 316.0097.

2-Bromo-3-(3-fluorophenylethynyl)-1,4-dimethoxybenzene (7d)

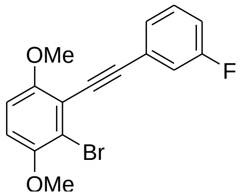

Reaction of **4f** (857 mg, 2.5 mmol) with 1-ethynyl-3-fluorobenzene (450 mg, 3.75 mmol) for 2.5 h at 75 °C following **GP2**, and purification by column chromatography (hexane/EtOAc, 10/1), afforded **7d** (779 mg, 93%) as a reddish brown solid. Mp 84–86 °C. ^{1}H NMR (300 MHz, $CDCl_3$): δ 3.83 (s, 3H), 3.85 (s, 3H), 6.75–6.85 (m, 2H), 7.04 (ddt, *J* = 8.3, 2.3, 1.1 Hz, 1H), 7.26–7.35 (m, 2H), 7.39 (d, *J* = 7.5 Hz, 1H) ppm. ^{13}C NMR (75.4 MHz, $CDCl_3$) δ 56.5 (CH_3), 56.8 (CH_3), 85.8 (C), 96.9 (d, *J* = 3.5 Hz, C), 110.0 (CH), 112.6 (CH), 115.83 (C),115.8 (d, *J* = 21.2 Hz, CH), 116.0 (C), 118.4 (d, *J* = 22.8 Hz, CH), 125.1 (d, *J* = 9.5 Hz, C), 127.6 (CH), 129.9 (d, *J* = 8.6 Hz, CH), 150.5 (C), 155.2 (C), 162.3 (d, *J* = 246.4 Hz, C) ppm. LRMS (70 eV, EI): *m/z* (%) 336 (M^++2, 97), 334 (M^+, 100), 321 (35), 319 (36), 278 (12), 276 (12), 225 (18), 197 (29). HRMS (EI) calcd for $C_{16}H_{12}BrFO_2$, 334.0005; found, 334.0005.

General procedure 4 (GP4) for the synthesis of dimethoxy-substituted benzo[*b*]furans 8–10 [3]. **Method C**: A mixture of the corresponding *o*-alkynylhaloarene derivative **5-7** (0.5 mmol), $Pd_2(dba)_3$ (9.2 mg, 0.01 mmol, 2 mol %),

t-BuXPhos (17 mg, 0.04 mmol, 8 mol %) and finely powdered KOH (0.84 g, 1.5 mmol) was evacuated and backfilled with nitrogen. H_2O (0.5 mL) and 1,4-dioxane (1 mL) were added and the mixture was stirred at 100 °C overnight. **Method D**: Alternatively, the reaction mixture was charged in a 10 mL thick-walled sealed glass tube and irradiated, under stirring, at 150 °C in the microwave cavity for 12 min (CEM Focused Microwave System, Discover S-Class), with a maximum wattage supplied of 50 W.

The reaction mixture was cooled to room temperature and extracted with Et_2O (3 × 15 mL). The combined organic layers were washed with water (2 × 20 mL). The organic layer was dried over anhydrous Na_2SO_4 and concentrated under reduced pressure. The remaining residue was purified by column chromatography on silica gel (eluent: hexane/EtOAc).

2-Butyl-4,5-dimethoxybenzo[*b*]furan (8a)

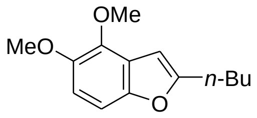

Reaction of **5a** (126 mg, 0.5 mmol) following **GP4** under microwave irradiation, and purification by column chromatography (hexane/EtOAc, 30/1), afforded **8a** (64 mg, 55%) as a yellow oil. R_f 0.45 (hexane/EtOAc, 20/1). ^{1}H NMR (300 MHz, $CDCl_3$): δ 0.95 (t, J = 7.4 Hz, 3H), 1.35–1.49 (m, 2H), 1.66–1.78 (m, 2H), 2.73 (t, J = 6.8 Hz, 2H), 3.88 (s, 3H), 4.03 (s, 3H), 6.49 (s, 1H), 6.84 (d, J = 8.8 Hz, 1H), 7.05 (d, J = 8.8 Hz, 1H) ppm. ^{13}C NMR (75.4 MHz, $CDCl_3$) δ 14.0 (CH_3), 22.4 (CH_2), 28.3 (CH_2), 29.8 (CH_2), 57.7 (CH_3), 60.6 (CH_3), 99.7 (CH), 105.0 (CH), 110.0 (CH), 122.2 (C), 141.4 (CH), 146.6 (C), 151.0 (C), 159.9 (C) ppm. LRMS (70 eV, EI): *m/z* (%) 234 (M^+, 56), 191 (95), 147 (100), 133 (84), 107 (58). HRMS (EI) calcd for $C_{14}H_{18}O_3$, 234.1256; found, 234.1253.

2-Cyclohex-1-enyl-4,5-dimethoxybenzo[*b*]furan (8b)

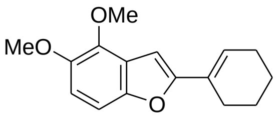

Reaction of **5b** (139 mg, 0.5 mmol) following **GP4** under conventional heating, and purification by column chromatography (hexane/EtOAc, 30/1), afforded **8b** (74 mg, 57%) as a yellow solid. Mp 48–50 °C. ^{1}H NMR (300 MHz, $CDCl_3$): δ 1.61–1.84 (m, 4H), 2.22–2.30 (m, 2H), 2.34–2.43 (m, 2H), 3.89 (s, 3H), 4.04 (s, 3H), 6.54–6.60 (m, 1H), 6.61 (s, 1H), 6.86 (d, *J* = 8.8 Hz, 1H), 7.06 (d, *J* = 8.8 Hz, 1H) ppm. ^{13}C NMR (75.4 MHz, $CDCl_3$) δ 22.2 (CH_2), 22.4 (CH_2), 25.0 (CH_2), 25.5 (CH_2), 57.5 (CH_3), 60.7 (CH_3), 97.9 (CH), 105.0 (CH), 110.6 (CH), 122.5 (C), 126.4 (CH), 127.1 (C), 141.6 (C), 146.6 (C), 150.7 (C), 157.6 (C) ppm. LRMS (70 eV, EI): *m/z* (%) 258 (M^+, 100), 243 (34), 230 (10). HRMS (EI) calcd for $C_{16}H_{18}O_3$, 258.1256; found, 258.1264.

2-Hexyl-4,5-dimethoxybenzo[*b*]furan (8c)

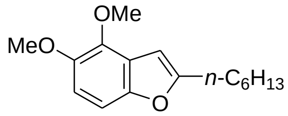

Reaction of **5c** (163 mg, 0.5 mmol) following **GP4** under microwave irradiation, and purification by column chromatography (hexane/EtOAc, 40/1), afforded **8c** (72 mg, 55%) as a yellow oil. R_f 0.44 (hexane/EtOAc, 10/1). ^{1}H NMR (300 MHz, $CDCl_3$): δ 0.89 (t, *J* = 6.5 Hz, 3H), 1.26–1.44 (m, 6H), 1.67–1.78 (m, 2H), 2.72 (t, *J* = 7.6 Hz, 2H), 3.88 (s, 3H), 4.03 (s, 3H), 6.49 (s, 1H), 6.84 (d, *J* = 8.7 Hz, 1H), 7.05 (d, *J* = 8.7 Hz, 1H) ppm. ^{13}C NMR (75.4 MHz, $CDCl_3$) δ 14.2 (CH_3), 22.7 (CH_2), 27.7 (CH_2), 28.6 (CH_2), 29.0 (CH_2), 31.7 (CH_2), 57.6 (CH_3), 60.5 (CH_3), 99.7 (CH), 105.0 (CH), 110.0 (CH), 122.2 (C), 141.3 (C), 146.6 (C), 151.0 (C), 159.9 (C) ppm. LRMS (70 eV, EI): *m/z* (%) 262 (M^+, 100), 247 (33), 191 (59), 147 (22). HRMS (EI) calcd for $C_{16}H_{22}O_3$, 262.1569; found, 262.1565.

4,5-Dimethoxy-2-phenylbenzo[*b*]furan (8d)

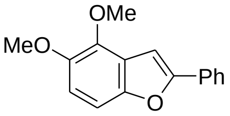

Reaction of **5d** (158 mg, 0.5 mmol) following **GP4** under conventional heating, and purification by column chromatography (hexane/EtOAc, 40/1), afforded **8d** (64 mg, 50%) as a yellow solid. Mp 68–70 °C. ^{1}H NMR (300 MHz, $CDCl_3$): δ 3.92 (s, 3H), 4.10 (s, 3H), 6.94 (d, *J* = 8.8 Hz, 1H), 7.14–7.20 (m, 2H), 7.31–7.39 (m, 1H), 7.41–7.48 (m, 2H), 7.82–7.87 (m, 2H) ppm. ^{13}C NMR (75.4 MHz, $CDCl_3$) δ 57.6 (CH_3), 60.8 (CH_3), 99.2 (CH), 105.5 (CH), 111.3 (CH), 122.8 (C), 125.0 (2 × CH), 128.7 (CH), 128.9 (2 × CH), 130.4 (C), 141.8 (C), 147.0 (C), 151.1 (C), 156.0 (C) ppm. LRMS (70 eV, EI): *m/z* (%) 254 (M^+, 100), 239 (42), 196 (24), 165 (15), 105 (17). HRMS (EI) calcd for $C_{16}H_{14}O_3$, 254.0943; found, 254.0950.

2-Butyl-4,6-dimethoxybenzo[*b*]furan (9a)

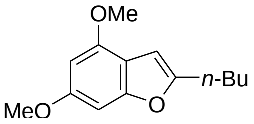

Reaction of **6a** (126 mg, 0.5 mmol) following **GP4** under conventional heating, and purification by column chromatography (hexane/EtOAc, 50/1), afforded **9a** (73 mg, 62%) as a white solid. Mp 51–53 °C. ^{1}H NMR (300 MHz, $CDCl_3$): δ 0.96 (t, *J* = 7.3 Hz, 3H), 1.35–1.49 (m, 2H), 1.65–1.77 (m, 2H), 2.73 (t, *J* = 7.5 Hz, 2H), 3.83 (s, 3H), 3.90 (s, 3H), 6.31 (s, 1H), 6.38 (s, 1H), 6.63 (s, 1H) ppm. ^{13}C NMR (75.4 MHz, $CDCl_3$) δ 13.9 (CH_3), 22.3 (CH_2), 28.1 (CH_2), 30.0 (CH_2), 55.6 (CH_3), 55.8 (CH_3), 88.2 (CH), 93.9 (CH), 98.8 (CH), 112.5 (C), 152.9 (C), 156.3 (C), 157.2 (C), 158.3 (C) ppm. LRMS (70 eV, EI): *m/z* (%) 234 (M^+, 31), 191 (100). HRMS (EI) calcd for $C_{14}H_{18}O_3$, 234.1256; found, 234.1259.

2-Cyclohex-1-enyl-4,6-dimethoxybenzo[*b*]furan (9b)

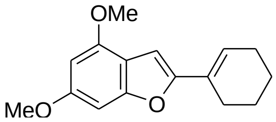

Reaction of **6b** (138 mg, 0.5 mmol) following **GP4** under conventional heating, and purification by column chromatography (hexane/EtOAc, 40/1), afforded **9b** (94 mg, 73%) as a white solid. Mp 65–67 °C. ^{1}H NMR (300 MHz, $CDCl_3$): δ 1.63–1.83 (m, 4H), 2.21–2.30 (m, 2H), 2.32–2.40 (m, 2H), 3.83 (s, 3H), 3.89 (s, 3H), 6.29 (d, *J* = 1.9 Hz, 1H), 6.45–6.50 (m, 1H), 6.51 (s, 1H), 6.62 (d, *J* = 1.9 Hz, 1H) ppm. ^{13}C NMR (75.4 MHz, $CDCl_3$) δ 22.3 (CH_2), 22.5 (CH_2), 24.9 (CH_2), 25.4 (CH_2), 55.6 (CH_3), 55.8 (CH_3), 88.1 (CH), 93.9 (CH), 97.4 (CH), 113.0 (C), 124.2 (CH), 127.2 (C), 153.3 (C), 155.4 (C), 156.2 (C), 158.9 (C) ppm. LRMS (70 eV, EI): *m/z* (%) 258 (M^+, 100), 243 (23), 230 (41), 215 (15). HRMS (EI) calcd for $C_{16}H_{18}O_3$, 258.1256; found, 258.1256.

4,6-Dimethoxy-2-pentylbenzo[*b*]furan (9c)

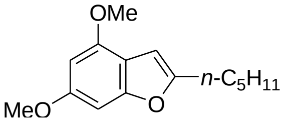

Reaction of **6c** (156 mg, 0.5 mmol) following **GP4** under conventional heating, and purification by column chromatography (hexane/EtOAc, 50/1), afforded **9c** (87 mg, 70%) as a pale yellow oil. R_f 0.56 (hexane/EtOAc, 10/1). ^{1}H NMR (300 MHz, $CDCl_3$): δ 0.90 (t, *J* = 7.0 Hz, 3H), 1.31–1.40 (m, 4H), 1.66–1.76 (m, 2H), 2.71 (t, *J* = 7.4 Hz, 2H), 3.83 (s, 3H), 3.89 (s, 3H), 6.30 (d, *J* = 1.9 Hz, 1H), 6.37 (s, 1H), 6.61 (s, 1H) ppm. ^{13}C NMR (75.4 MHz, $CDCl_3$) δ 14.1 (CH_3), 22.6 (CH_2), 27.6 (CH_2), 28.4 (CH_2), 31.4 (CH_2), 55.6 (CH_3), 55.9 (CH_3), 88.3 (CH), 93.9 (CH), 98.8 (CH), 112.6 (C), 152.9 (C), 156.3 (C), 157.3 (C), 158.3 (C) ppm. LRMS (70 eV, EI): *m/z* (%) 248 (M^+, 29), 191 (100), 176 (5). HRMS (EI) calcd for $C_{15}H_{20}O_3$, 248.1412; found, 248.1411.

4,6-Dimethoxy-2-phenylbenzo[*b*]furan (9d)

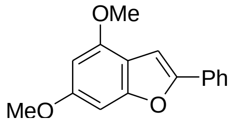

Reaction of **6d** (158 mg, 0.5 mmol) following **GP4** under conventional heating, and purification by column chromatography (hexane/EtOAc, 30/1), afforded **9d** (103 mg, 81%) as a white solid. Mp 69–71 °C. ^{1}H NMR (300 MHz, $CDCl_3$): δ 3.87 (s, 3H), 3.93 (s, 3H), 6.34 (d, *J* = 1.9 Hz, 1H), 6.70–6.72 (m, 1H), 7.05 (s, 1H), 7.25–7.35 (m, 1H), 7.39–7.47 (m, 2H), 7.78–7.85 (m, 2H) ppm. ^{13}C NMR (75.4 MHz, $CDCl_3$) δ 55.7 (CH_3), 55.9 (CH_3), 88.3 (CH), 94.4 (CH), 98.9 (CH), 113.3 (C), 124.3 (2 $\times$ CH), 127.9 (CH), 128.9 (2 $\times$ CH), 130.9 (C), 153.6 (C), 153.8 (C), 156.7 (C), 159.3 (C) ppm. LRMS (70 eV, EI): *m/z* (%) 254 (M^+, 76), 239 (100), 152 (48), 127 (47), 105 (48), 77 (74). HRMS (EI) calcd for $C_{16}H_{14}O_3$, 254.0943; found, 254.0943.

4,6-Dimethoxy-2-*p*-tolylbenzo[*b*]furan (9e)

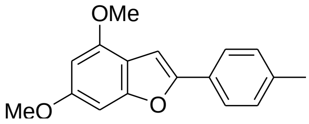

Reaction of **6e** (165 mg, 0.5 mmol) following **GP4** under conventional heating, and purification by column chromatography (hexane/EtOAc, 30/1), afforded **9e** (101 mg, 75%) as a brown solid. Mp 97–99 °C. ^{1}H NMR (300 MHz, $CDCl_3$): δ 2.40 (s, 3H), 3.87 (s, 3H), 3.93 (s, 3H), 6.35 (d, *J* = 1.8 Hz, 1H), 6.72 (d, *J* = 1.8 Hz, 1H), 7.01 (s, 1H), 7.25 (d, *J* = 8.0 Hz, 2H), 7.71 (d, *J* = 8.0 Hz, 2H) ppm. ^{13}C NMR (75.4 MHz, $CDCl_3$) δ 21.4 (CH_3), 55.6 (CH_3), 55.8 (CH_3), 88.3 (CH), 94.3 (CH), 98.1 (CH), 113.4 (C), 124.3 (2 $\times$ CH), 128.1 (C), 129.5 (2 $\times$ CH), 137.8 (C), 153.4 (C), 154.0 (C), 156.5 (C), 159.1 (C) ppm. LRMS (70 eV, EI): *m/z* (%) 268 (M^+, 100), 253 (86), 210 (11). HRMS (EI) calcd for $C_{17}H_{16}O_3$, 268.1099; found, 268.1097.

2-Butyl-4,7-dimethoxybenzo[*b*]furan (10a)

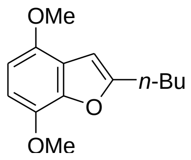

Reaction of **7a** (126 mg, 0.5 mmol) following **GP4** under conventional heating, and purification by column chromatography (hexane/EtOAc, 40/1), afforded **10a** (70 mg, 60%) as a yellow oil. R_f 0.28 (hexane/EtOAc, 20/1). ^{1}H NMR (300 MHz, $CDCl_3$): δ 0.94 (t, J = 7.3 Hz, 3H), 1.34–1.48 (m, 2H), 1.66–1.80 (m, 2H), 2.78 (t, J = 7.6 Hz, 2H), 3.87 (s, 3H), 3.96 (s, 3H), 6.46–6.52 (m, 2H), 6.62 (d, J = 8.5 Hz, 1H) ppm. ^{13}C NMR (75.4 MHz, $CDCl_3$) δ 13.9 (CH_3), 22.3 (CH_2), 28.1 (CH_2), 29.9 (CH_2), 55.8 (CH_3), 56.4 (CH_3), 99.6 (CH), 102.6 (CH), 105.2 (CH), 120.7 (C), 140.0 (C), 144.6 (C), 147.0 (C), 159.0 (C) ppm. LRMS (70 eV, EI): *m/z* (%) 234 (M^+, 37), 219 (100), 191 (30), 176 (16), 161.4 (14). HRMS (EI) calcd for $C_{14}H_{18}O_3$: 234.1256; found, 234.1263.

4,7-Dimethoxy-2-pentylbenzo[*b*]furan (10b)

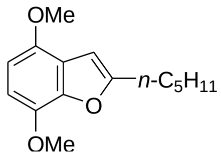

Reaction of **7b** (156 mg, 0.5 mmol) following **GP4** under conventional heating, and purification by column chromatography (hexane/EtOAc, 50/1), afforded **10b** (79 mg, 64%) as a pale yellow oil. R_f 0.53 (hexane/EtOAc, 8/1). ^{1}H NMR (300 MHz, $CDCl_3$): δ 0.90 (t, J = 7.0 Hz, 3H), 1.33–1.40 (m, 4H), 1.69–1.80 (m, 2H), 2.77 (t, J = 7.6 Hz, 2H), 3.88 (s, 3H), 3.96 (s, 3H), 6.46–6.48 (m, 1H), 6.50 (d, J = 8.6 Hz, 1H), 6.62 (d, J = 8.6 Hz, 1H) ppm. ^{13}C NMR (75.4 MHz, $CDCl_3$) δ 14.1 (CH_3), 22.5 (CH_2), 27.6 (CH_2), 28.4 (CH_2), 31.5 (CH_2), 55.9 (CH_3), 56.5 (CH_3), 99.6 (CH), 102.7 (CH), 105.2 (CH), 120.8 (C), 140.0 (C), 144.6 (C), 147.0 (C), 159.0 (C) ppm. LRMS (70 eV, EI): *m/z* (%) 248 (M^+, 79), 233 (100), 191 (31), 176 (17), 161 (18); HRMS (EI) calcd for $C_{15}H_{20}O_3$: 248.1412; found, 248.1415.

4,7-Dimethoxy-2-phenylbenzo[*b*]furan (10c)

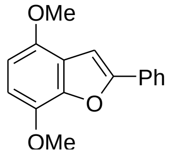

Reaction of **7c** (158 mg, 0.5 mmol) following **GP4** under microwave irradiation, and purification by column chromatography (hexane/EtOAc, 40/1), afforded **10c** (90 mg, 71%) as a yellow solid. Mp 75–77 °C. ^{1}H NMR (300 MHz, $CDCl_3$): δ 3.92 (s, 3H), 4.01 (s, 3H), 6.54 (d, *J* = 8.5 Hz, 1H), 6.70 (d, *J* = 8.5 Hz, 1H), 7.14 (s, 1H), 7.31–7.38 (m, 1H), 7.40–7.48 (m, 2H), 7.88–7.92 (m, 2H) ppm. ^{13}C NMR (75.4 MHz, $CDCl_3$) δ 55.9 (CH_3), 56.7 (CH_3), 99.3 (CH), 102.9 (CH), 106.9 (CH), 121.3 (C), 125.0 (CH), 128.5 (CH), 128.8 (CH), 130.4 (C), 140.2 (C), 145.0 (C), 147.5 (C), 155.2 (C) ppm. LRMS (70 eV, EI): *m/z* (%) 254 (M^+, 79), 239 (100), 224 (32), 139 (11). HRMS (EI) calcd for $C_{16}H_{14}O_3$: 254.0943; found, 254.0936.

2-(3-Fluorophenyl)-4,7-dimethoxybenzo[*b*]furan (10d)

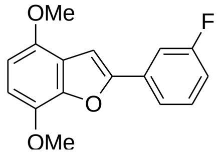

Reaction of **7d** (167 mg, 0.5 mmol) following **GP4** under microwace irradiation, and purification by column chromatography (hexane/EtOAc, 40/1), afforded **10d** (88 mg, 65%) as a white solid. Mp 105–107 °C. ^{1}H NMR (300 MHz, $CDCl_3$): δ (300 MHz, $CDCl_3$) 3.91 (s, 3H), 4.00 (s, 3H), 6.53 (d, *J* = 8.5 Hz, 1H), 6.71 (d, *J* = 8.5 Hz, 1H), 7.02 (td *J* = 8.3, 2.4 Hz, 1H), 7.14 (s, 1H), 7.38 (td, *J* = 8.0, 5.9 Hz, 1H), 7.54–7.60 (m, 1H), 7.64 (d, *J* = 7.8 Hz, 1H) ppm. ^{13}C NMR (75.4 MHz, $CDCl_3$) δ NMR (75.4 MHz, $CDCl_3$) 55.9 (CH_3), 56.7 (CH_3), 100.4 (CH), 103.0 (CH), 107.3 (CH), 111.8 (d, *J* = 23.6 Hz, CH), 115.2 (d, *J* = 21.3 Hz, CH), 120.6 (d, *J* = 2.9 Hz, CH), 121.1 (C), 130.4 (d, *J* = 8.4 Hz, CH), 132.5 (d, *J* = 8.4 Hz, C), 140.2 (C), 145.0 (C), 147.6 (C), 153.8 (d, *J* = 3.1 Hz, C), 163.2 (d, *J* = 245.5 Hz, C) ppm. LRMS (70 eV,

EI): *m/z* (%) 272 (M^+, 68), 257 (100), 242 (29), 214 (9). HRMS (EI) calcd for $C_{16}H_{13}FO_3$: 272.0849 ; found, 272.0849.

References

1. Sanz, R.; Castroviejo, M. P.; Guilarte, V.; Pérez, A.; Fañanás, F. J. *J. Org. Chem.* **2007,** *72,* 5113–5118. doi:10.1021/jo070643y
2. Liang, Y.; Xie, Y.-X.; Li, J.-H. *J. Org. Chem.* **2006,** *71,* 379–381. doi:10.1021/jo051882t
3. Anderson, K. W.; Ikawa, T.; Tundel, R. E.; Buchwald, S. L. *J. Am. Chem. Soc.* **2006,** *128,* 10694–10695. doi:10.1021/ja0639719
