# Supplementary material for: Combined directed ortho-zincation and palladium-catalyzed strategies: Synthesis of 4,n-dimethoxy-substituted benzo[b]furans
Source: Beilstein J Org Chem. 2011 Sep 12;7:1255–60. doi: 10.3762/bjoc.7.146 (PMC3182435; doi:10.3762/bjoc.7.146)

Supporting Information File 2

for

Combined directed *ortho*-zincation and palladium-catalyzed strategies: Synthesis of 4,n-dimethoxy-substituted benzo[*b*]furans

Verónica Guilarte, M. Pilar Castroviejo, Estela Álvarez and Roberto Sanz*

Address: Área de Química Orgánica, Departamento de Química, Facultad de Ciencias, Universidad de Burgos, Pza. Misael Bañuelos s/n, 09001 Burgos, Spain

Email: Roberto Sanz* - rsd@ubu.es

* Corresponding author

NMR spectra

^{1}H NMR (300 MHz, $CDCl_3$) (4a):

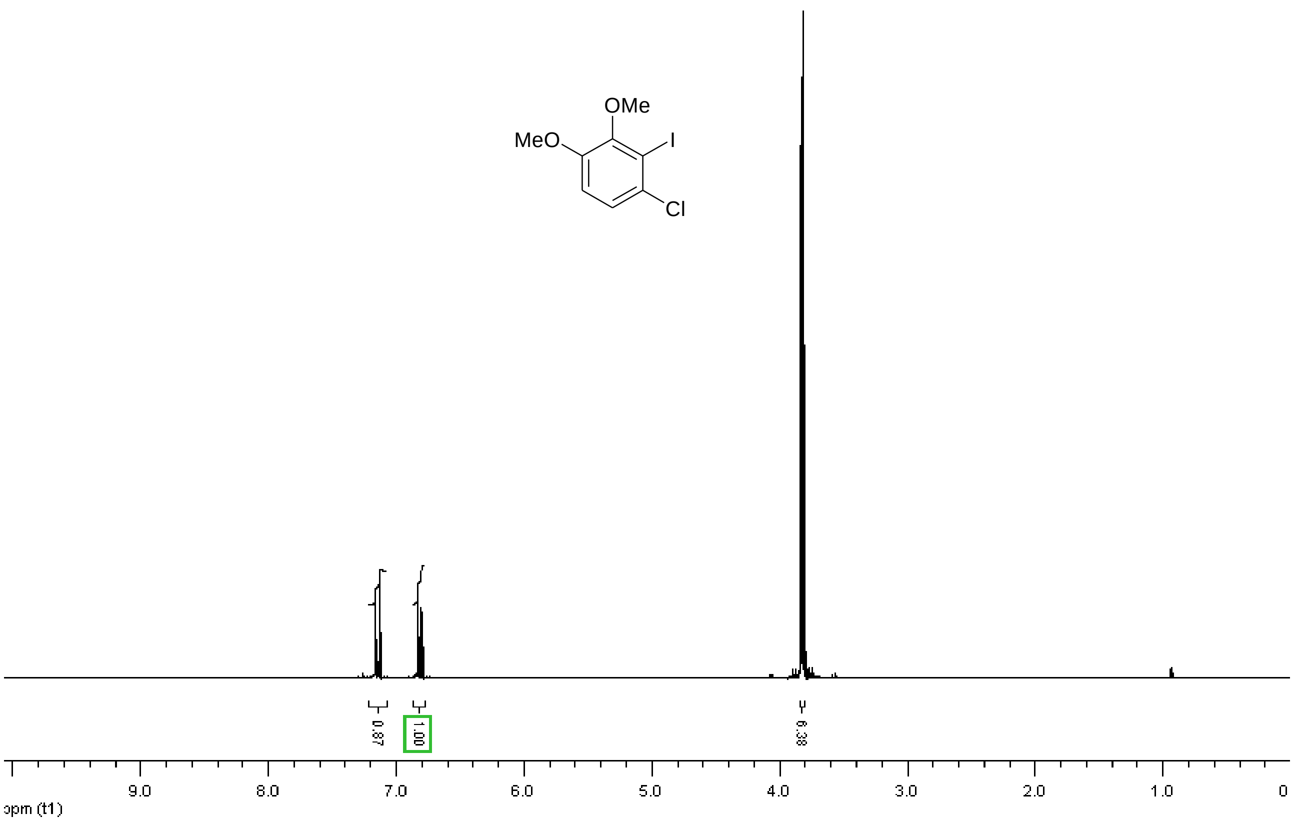

^{13}C NMR (75.4 MHz, $CDCl_3$):

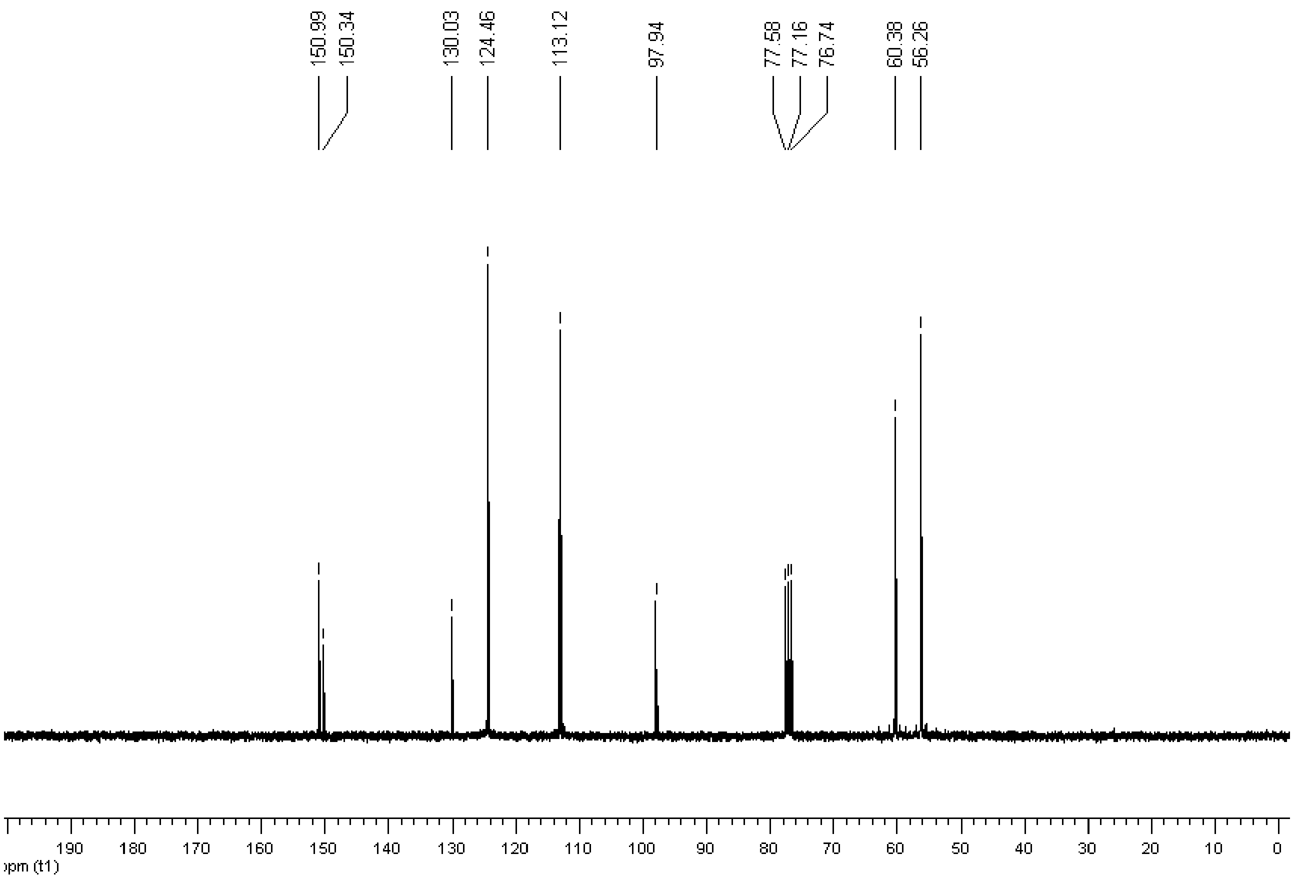

^{1}H NMR (300 MHz, $CDCl_3$) (4b):

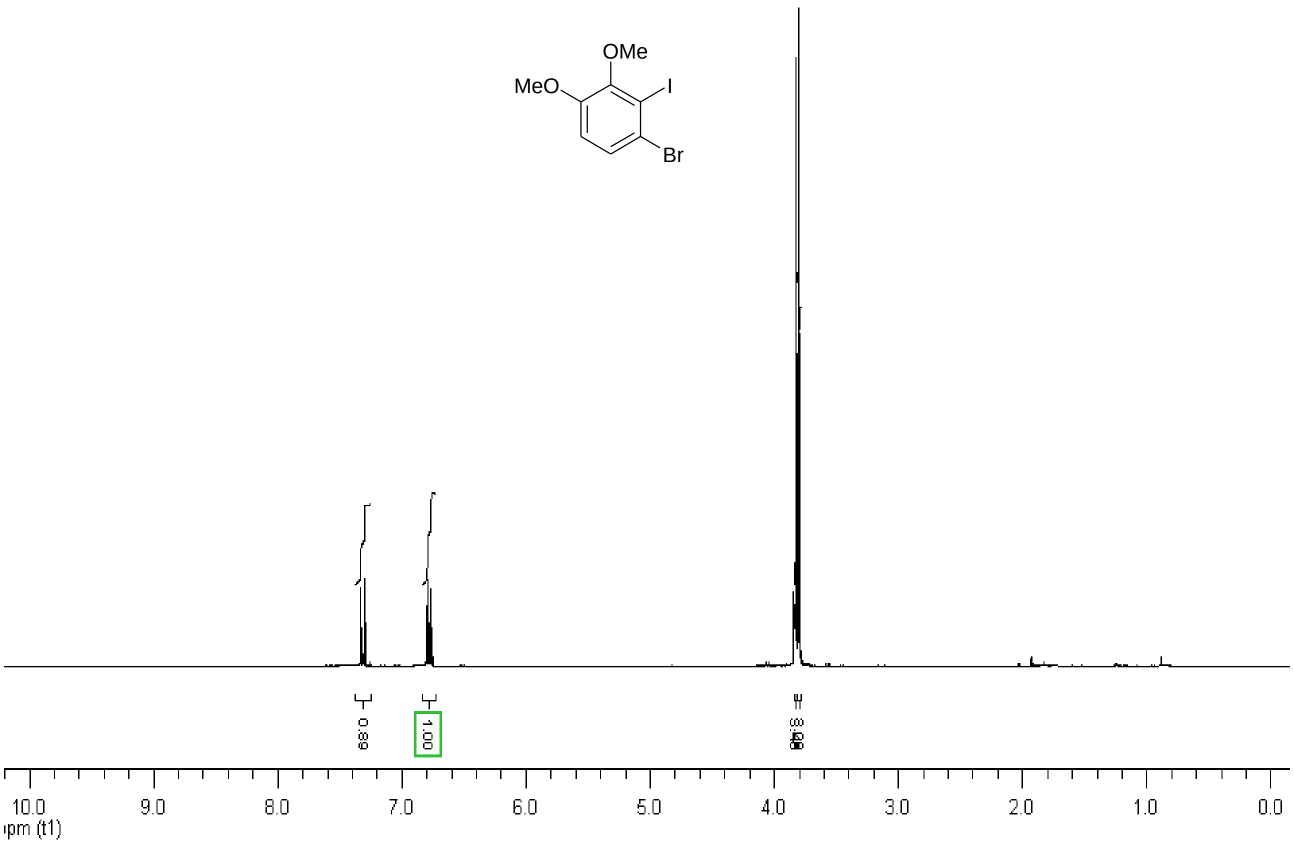

^{13}C NMR (75.4 MHz, $CDCl_3$):

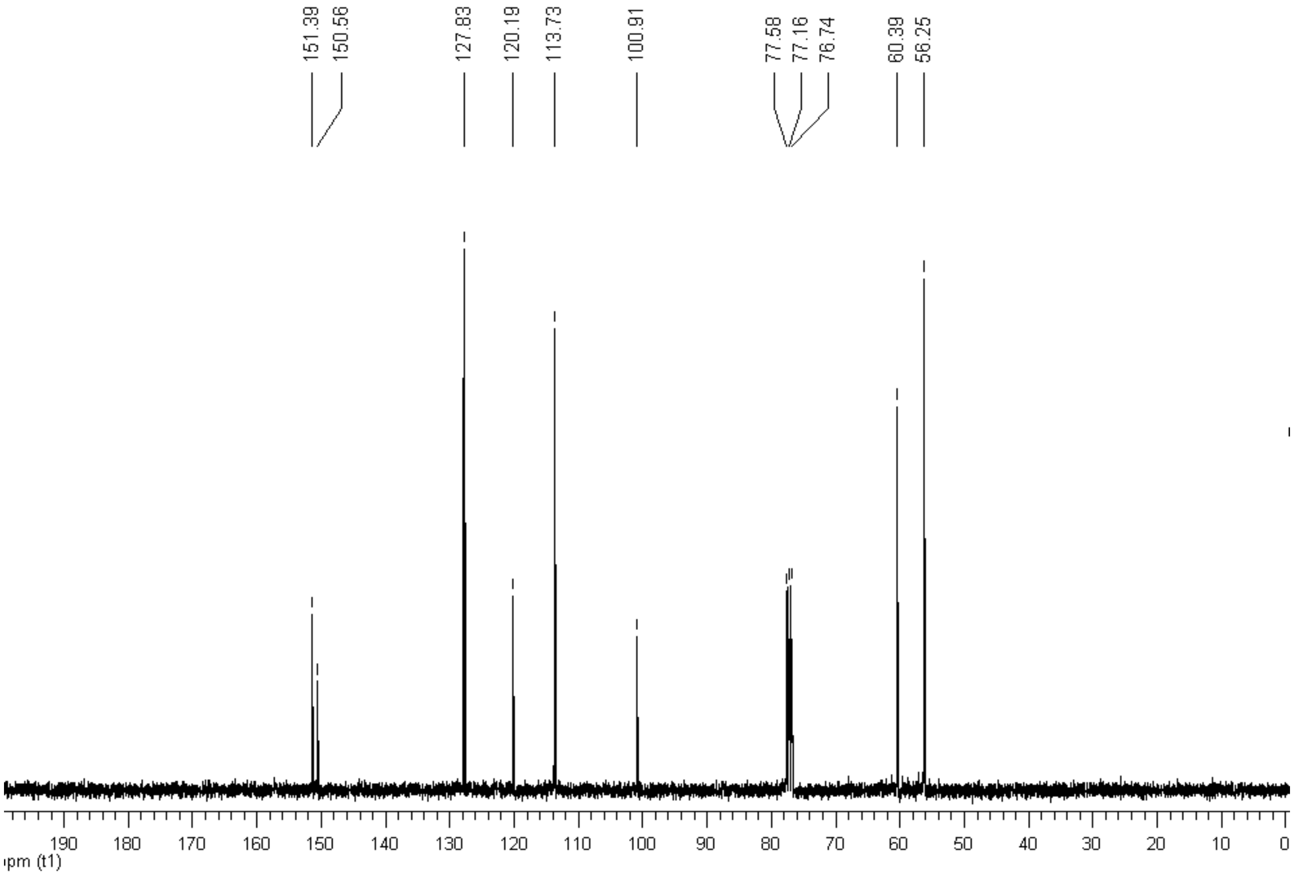

^{1}H NMR (300 MHz, $CDCl_3$) (4c):

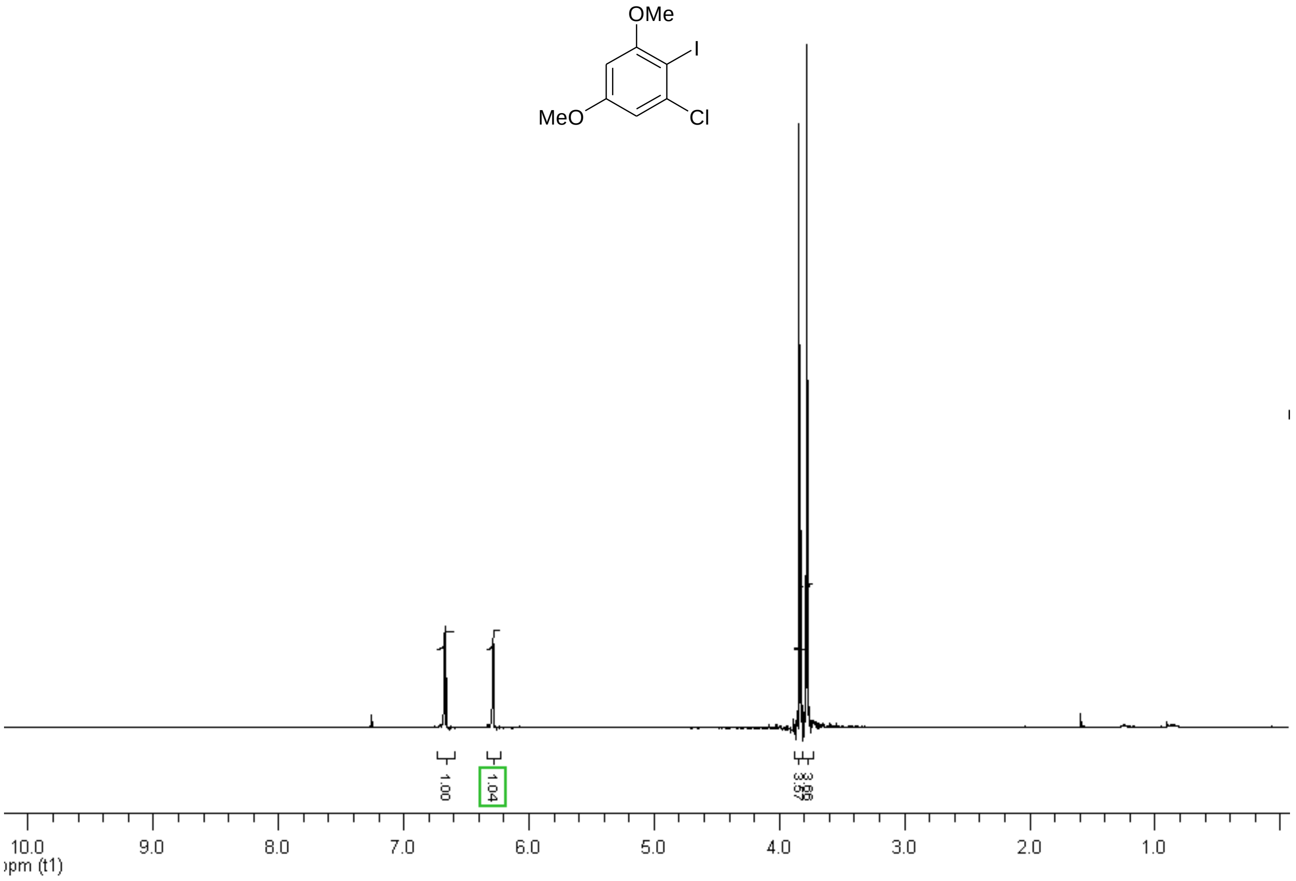

^{13}C NMR (75.4 MHz, $CDCl_3$):

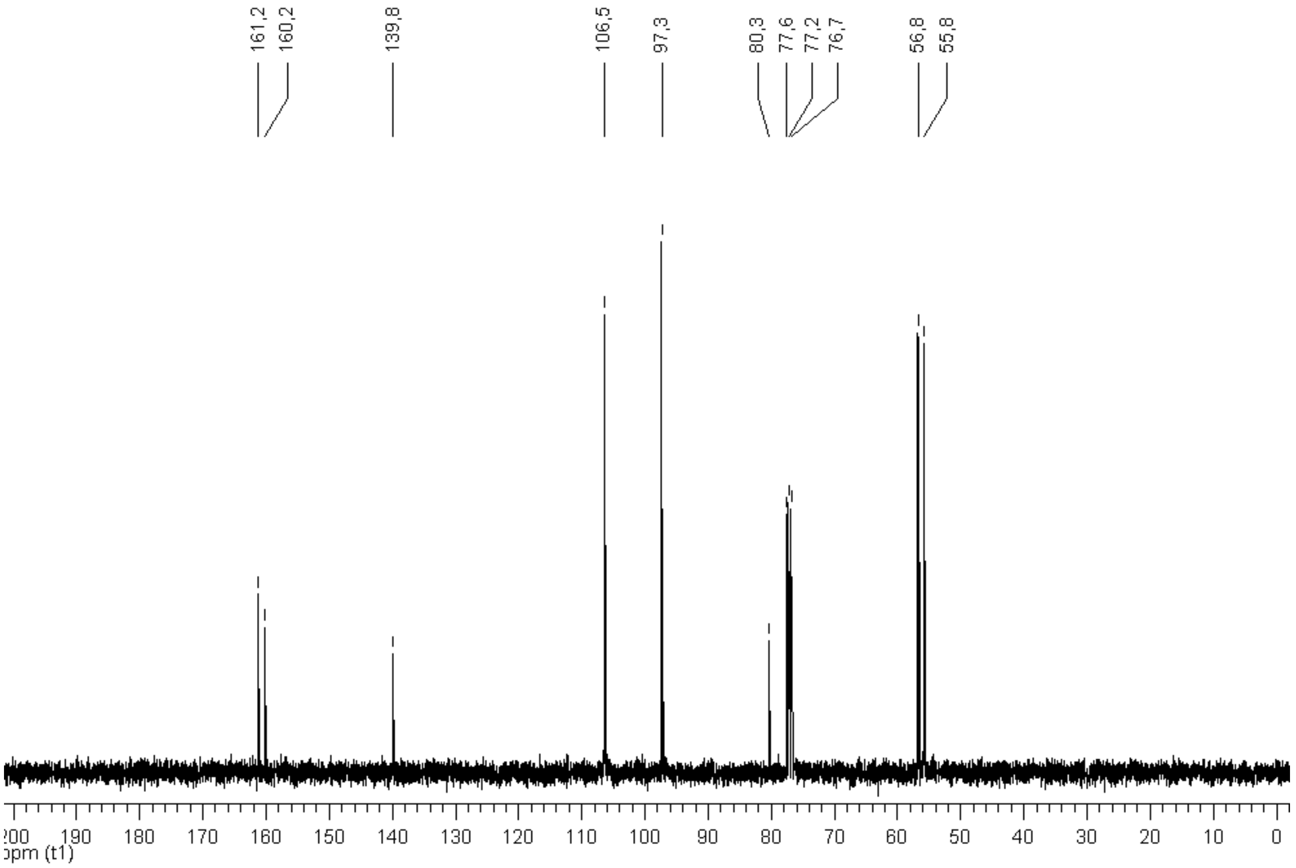

^{1}H NMR (300 MHz, $CDCl_3$) (4d):

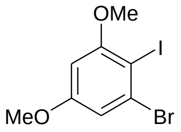

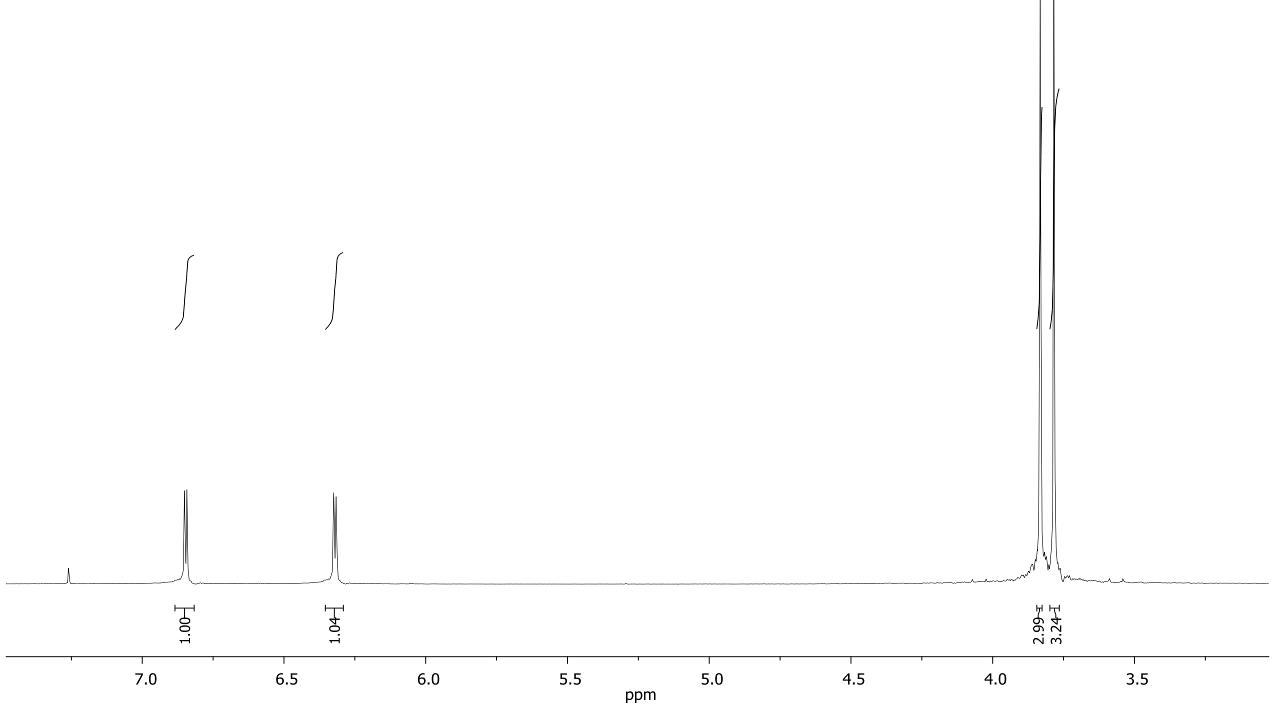

^{13}C NMR (75.4 MHz, $CDCl_3$):

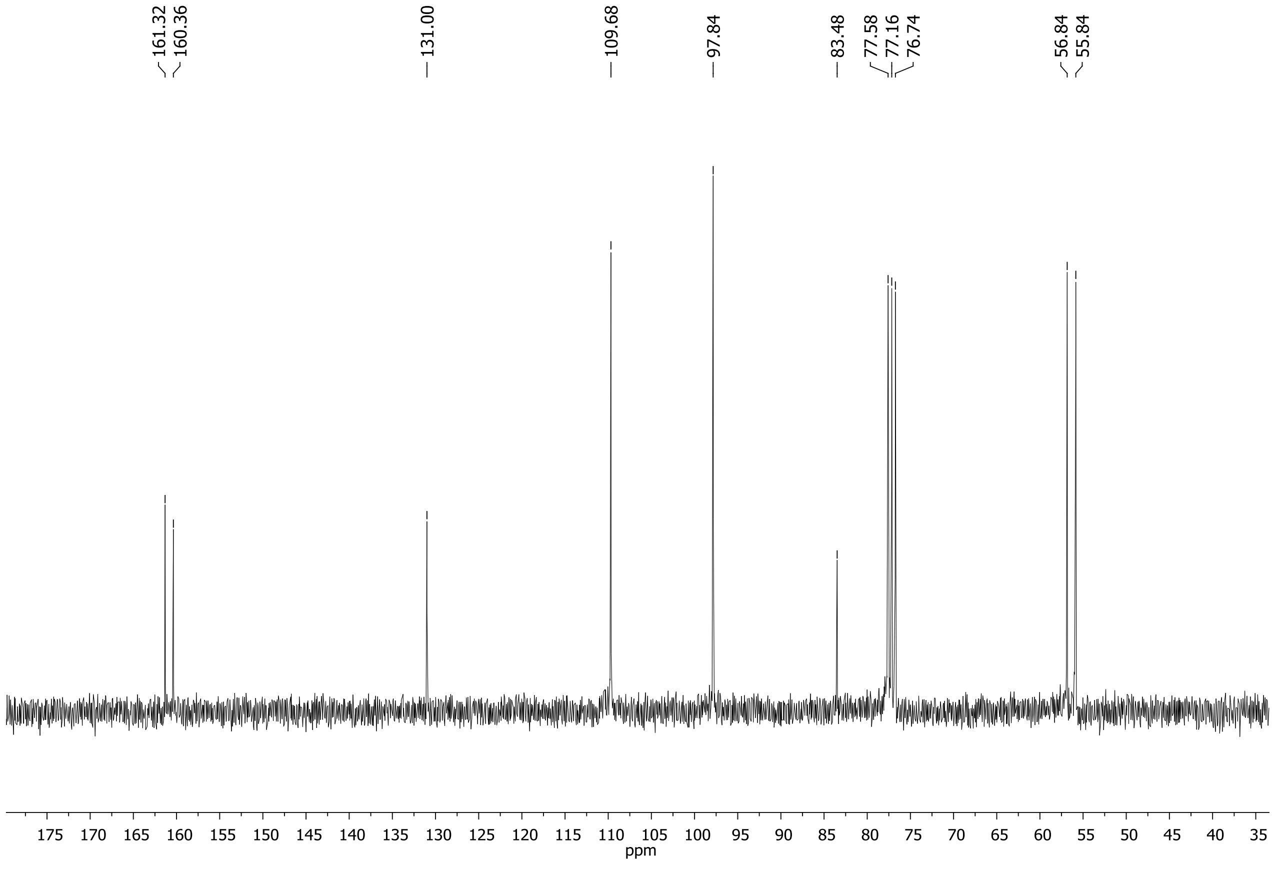

^{1}H NMR (400 MHz, $CDCl_3$) (4e):

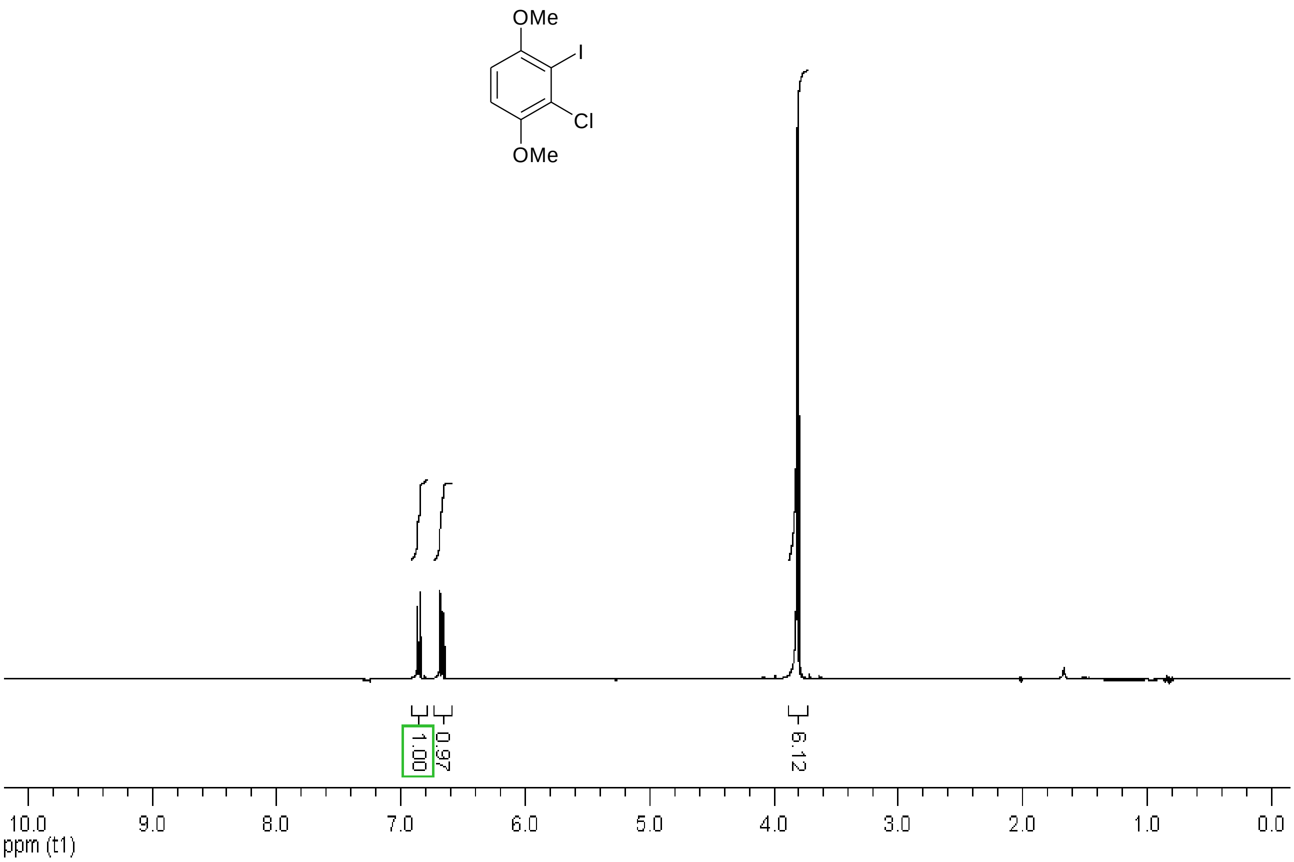

^{13}C NMR (100.6 MHz, $CDCl_3$):

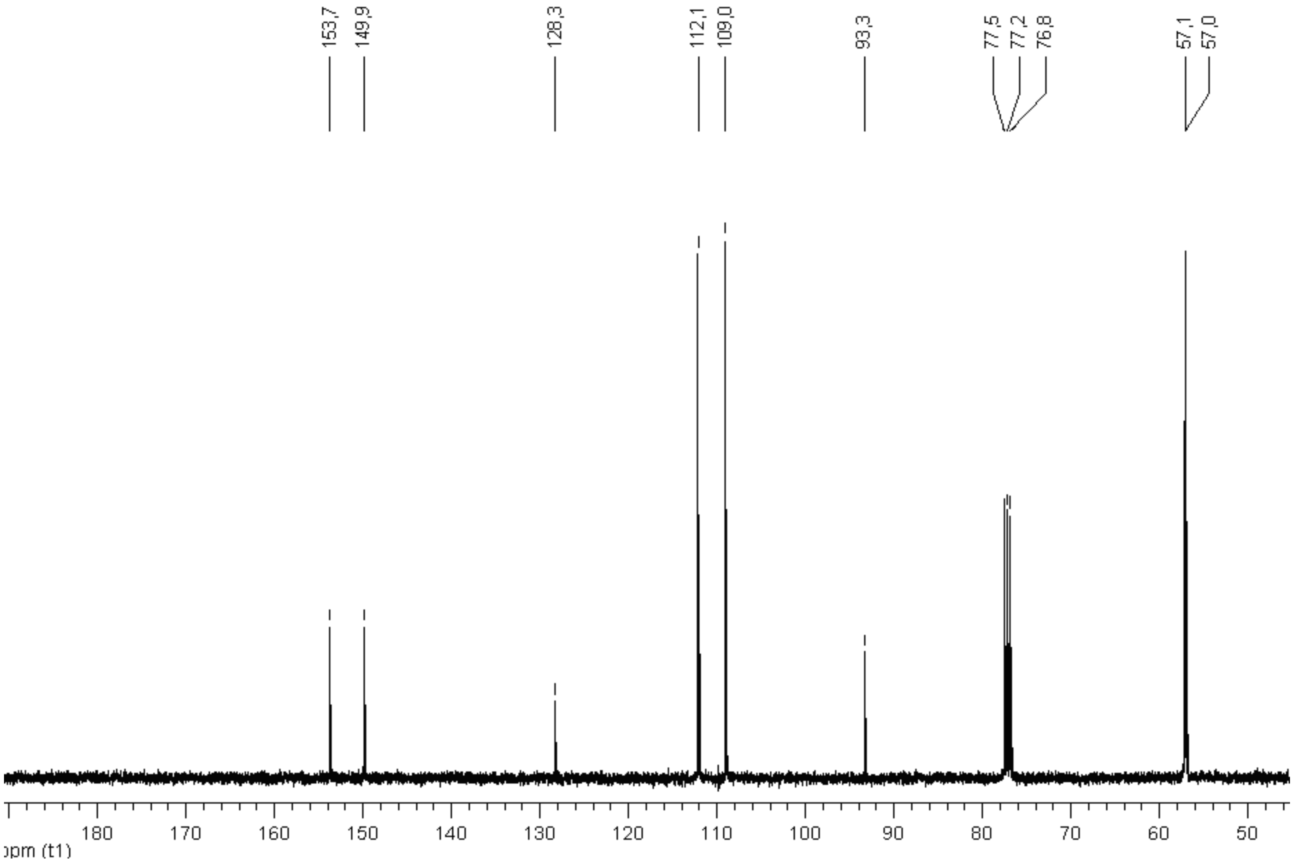

^{1}H NMR (300 MHz, $CDCl_3$) (4f):

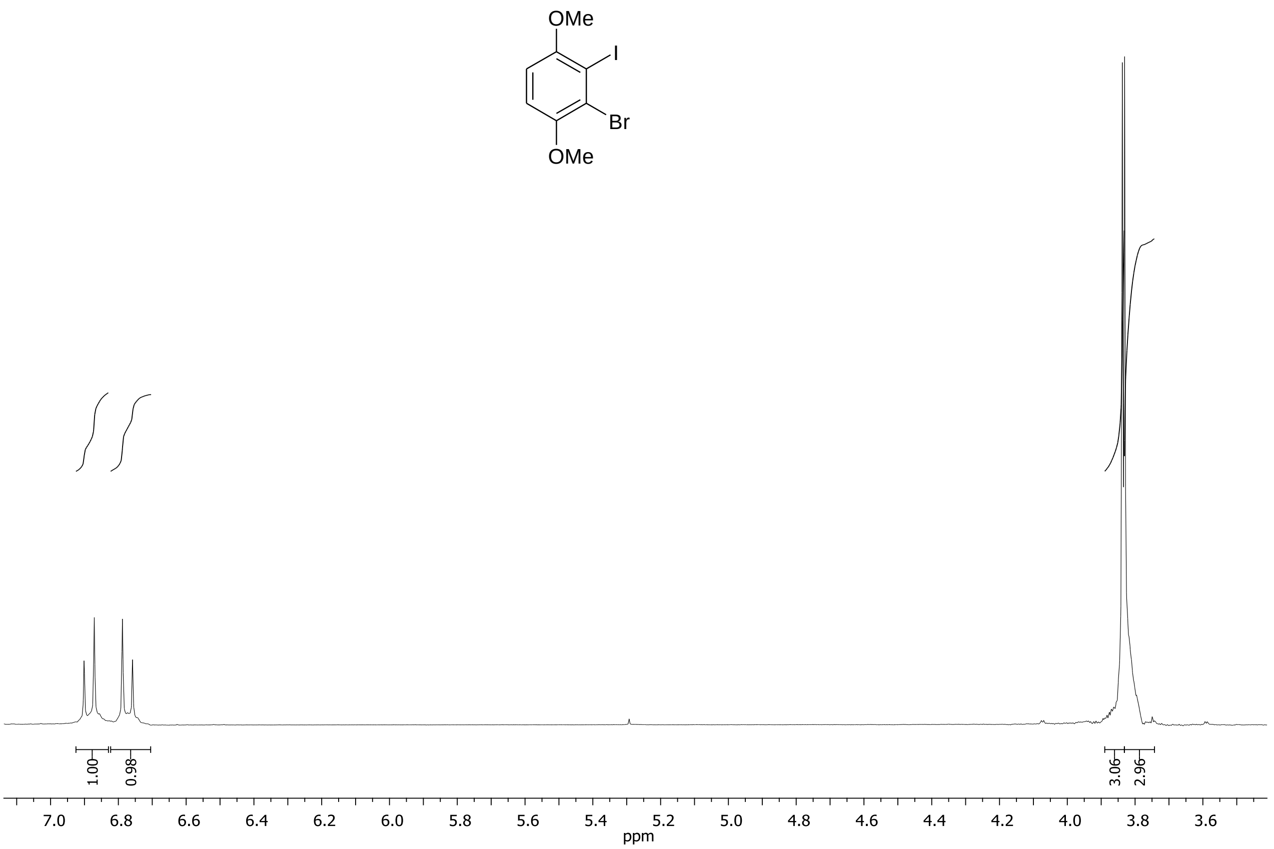

^{13}C NMR (75.4 MHz, $CDCl_3$):

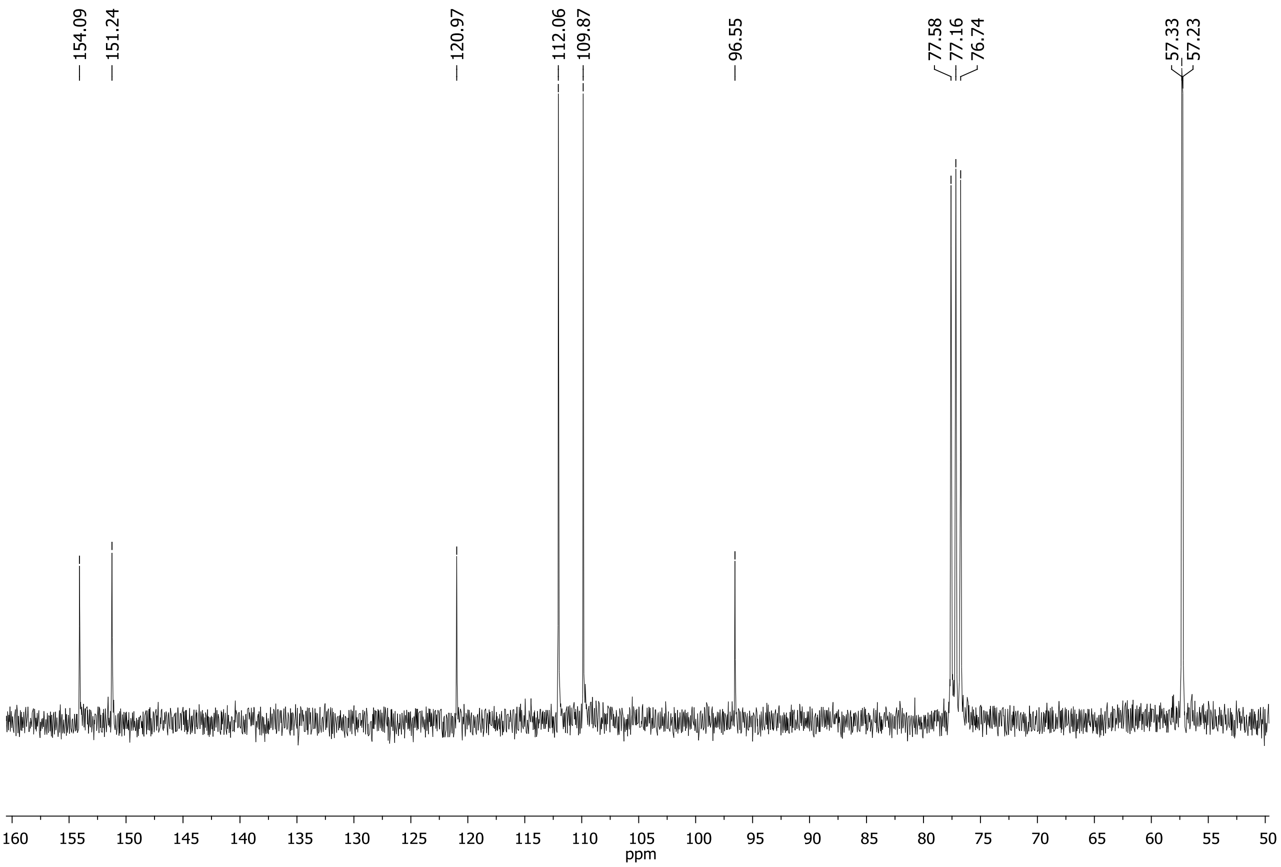

^{1}H NMR (300 MHz, $CDCl_3$) (5a):

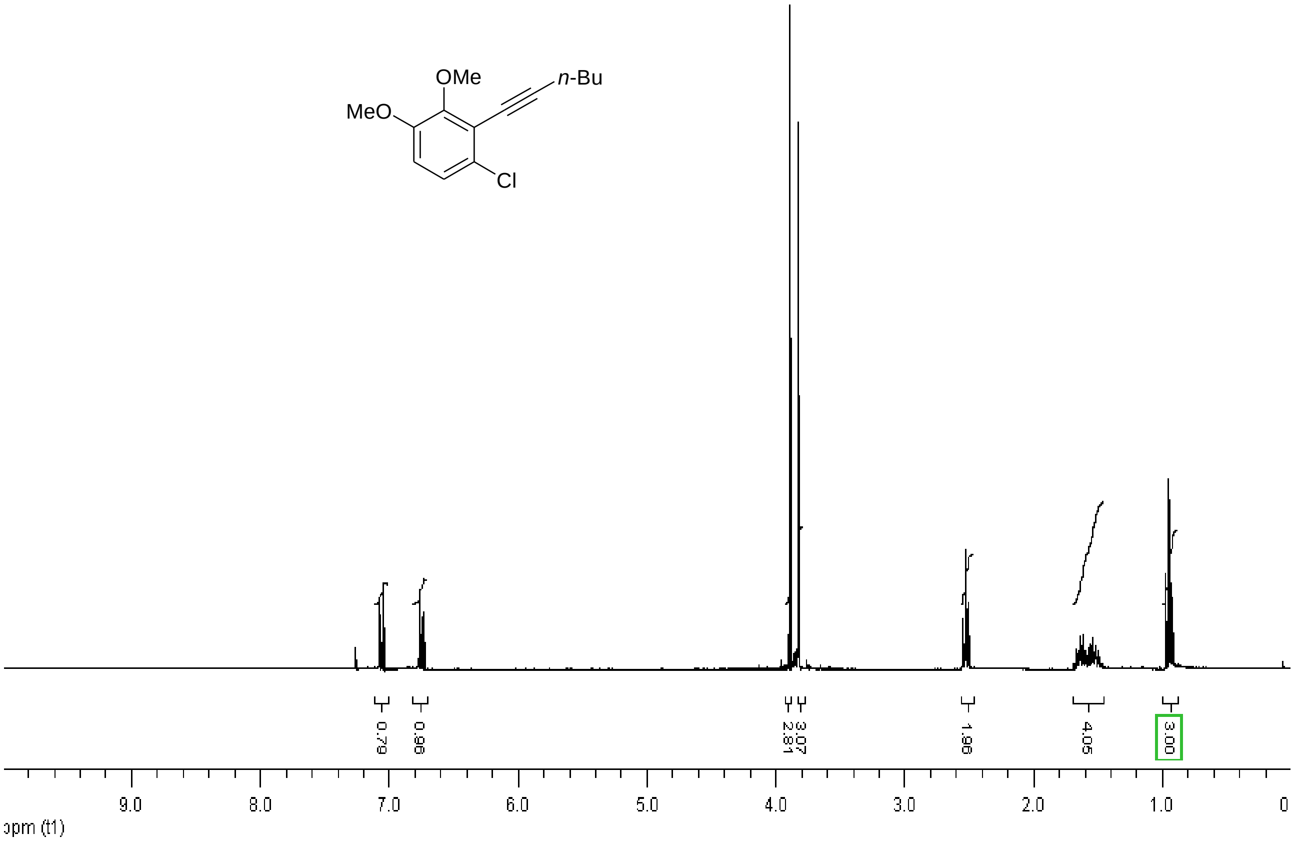

^{13}C NMR (75.4 MHz, $CDCl_3$):

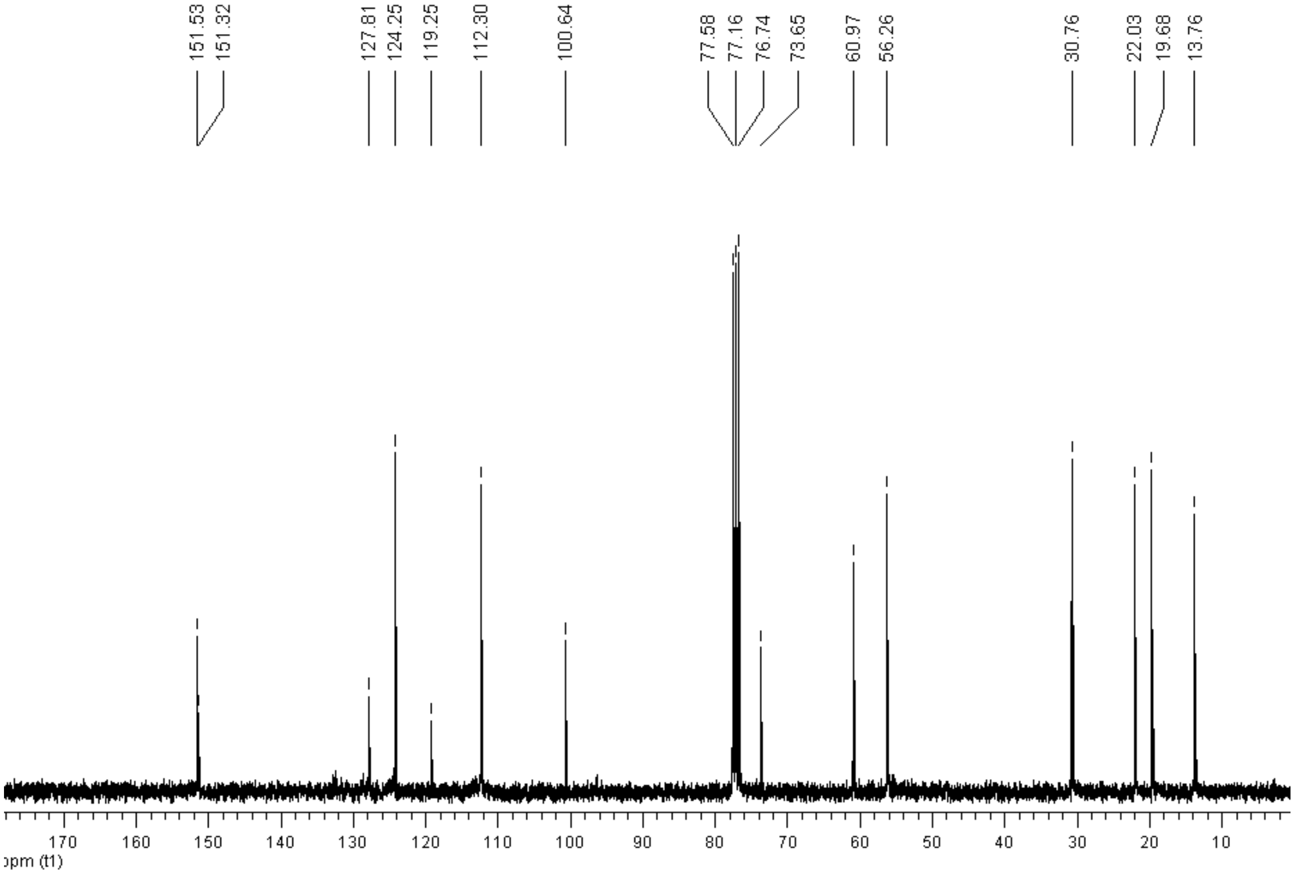

^{1}H NMR (300 MHz, $CDCl_3$) (5b):

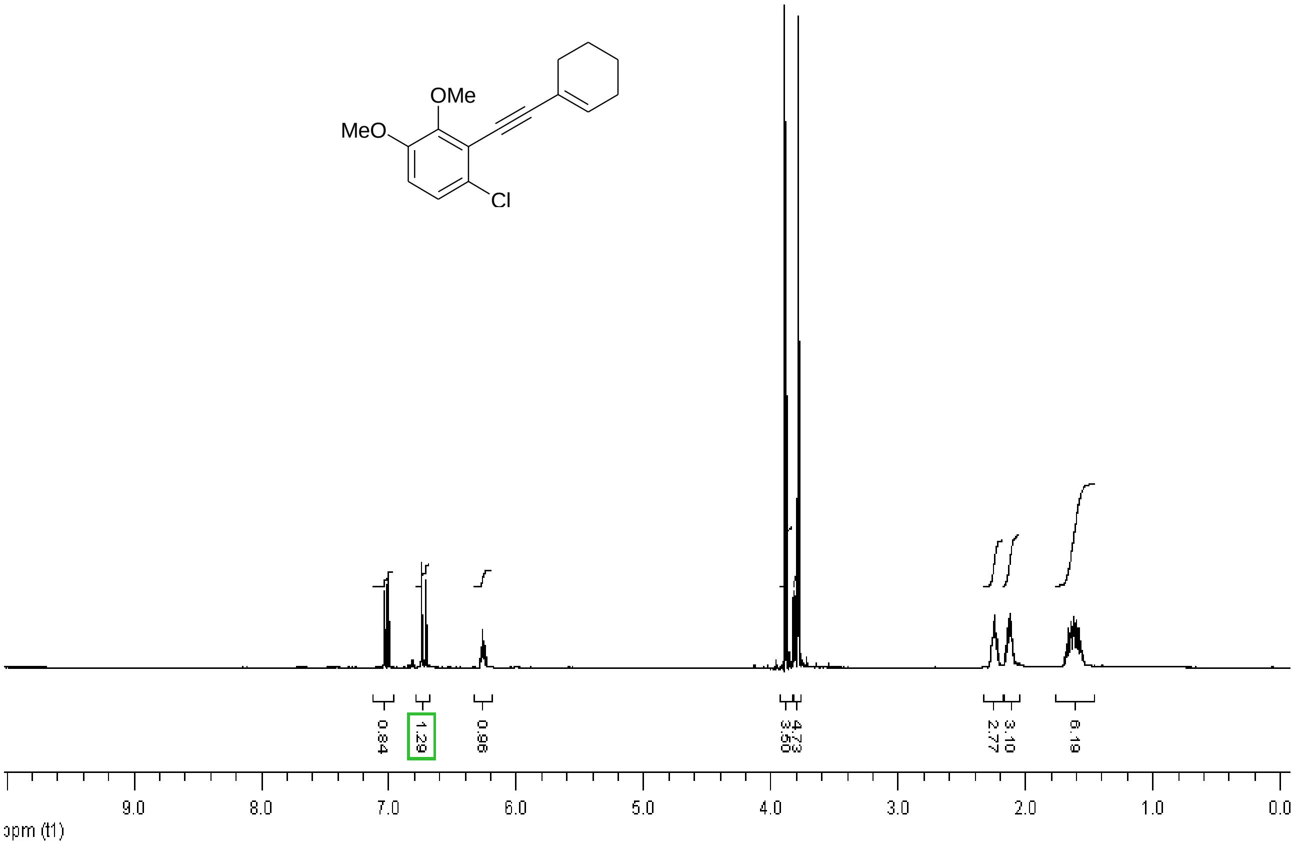

^{13}C NMR (75.4 MHz, $CDCl_3$):

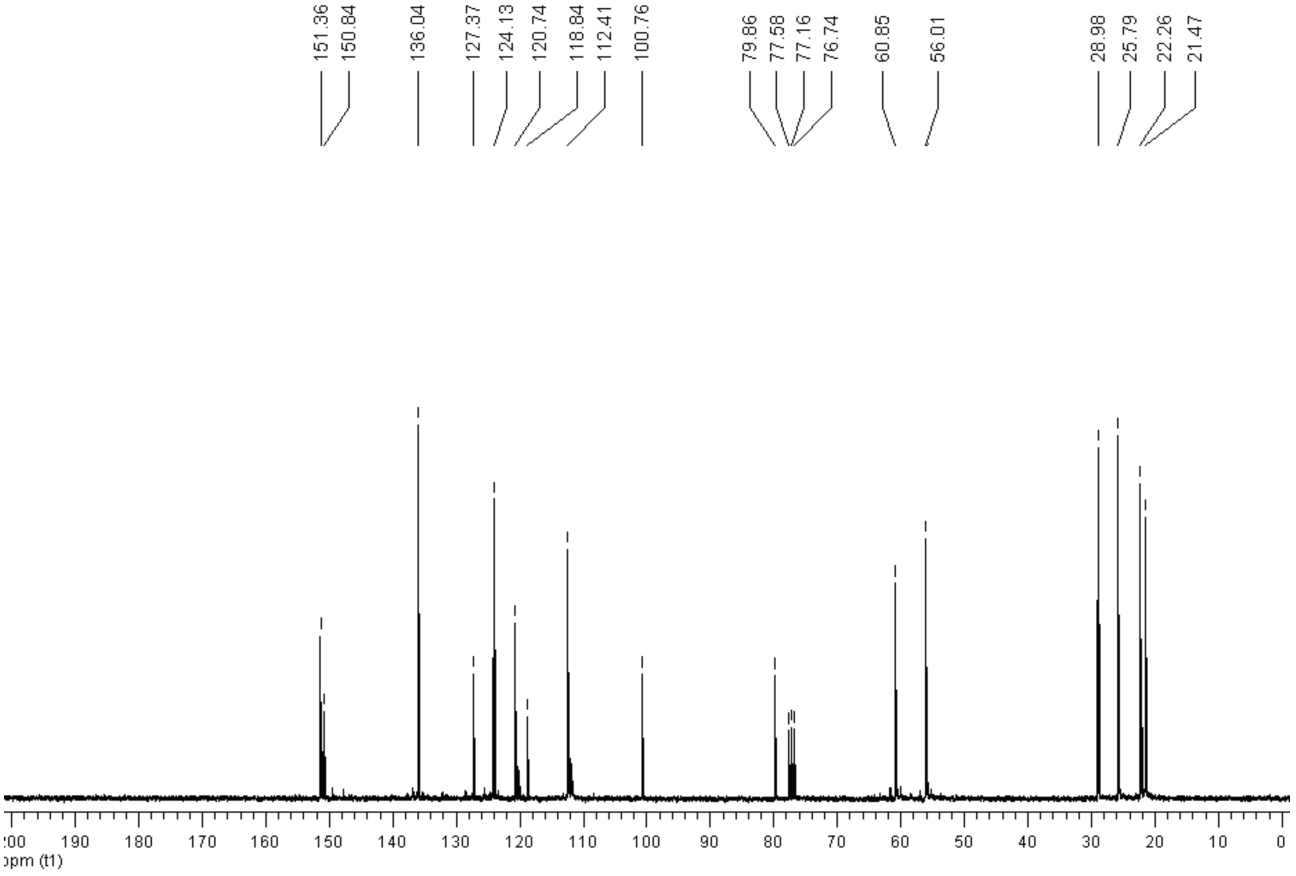

^{1}H NMR (300 MHz, CDCl$_3$) (5c):

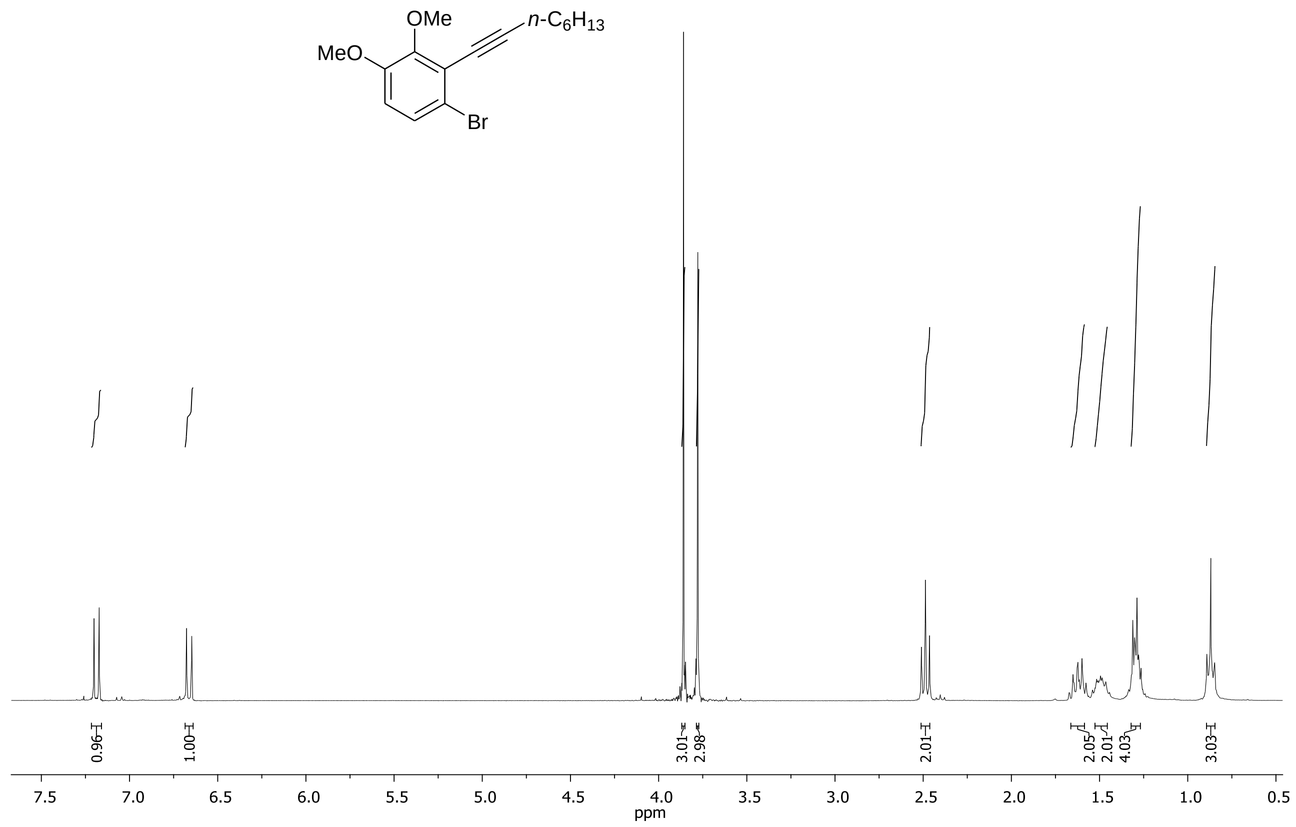

^{13}C NMR (75.4 MHz, CDCl$_3$):

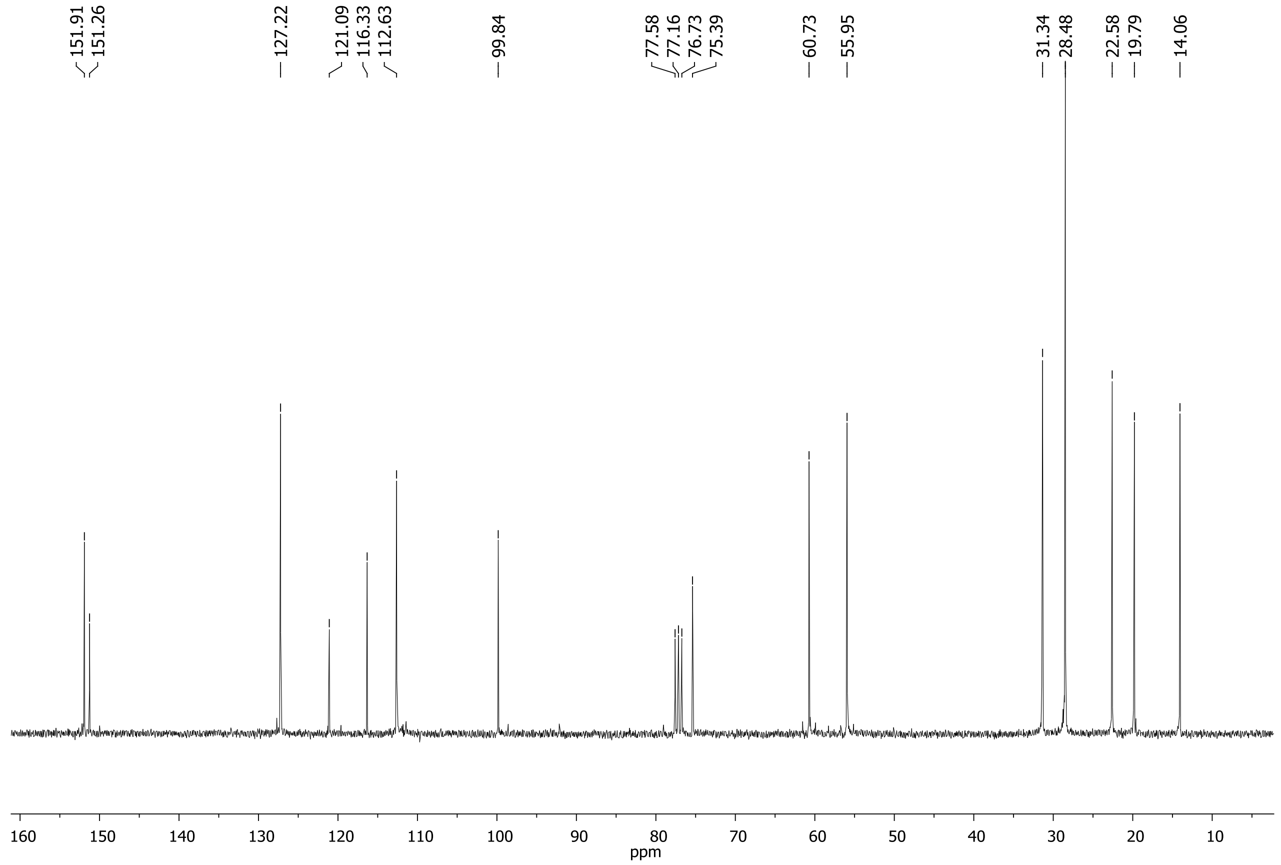

^{1}H NMR (300 MHz, $CDCl_3$) (5d):

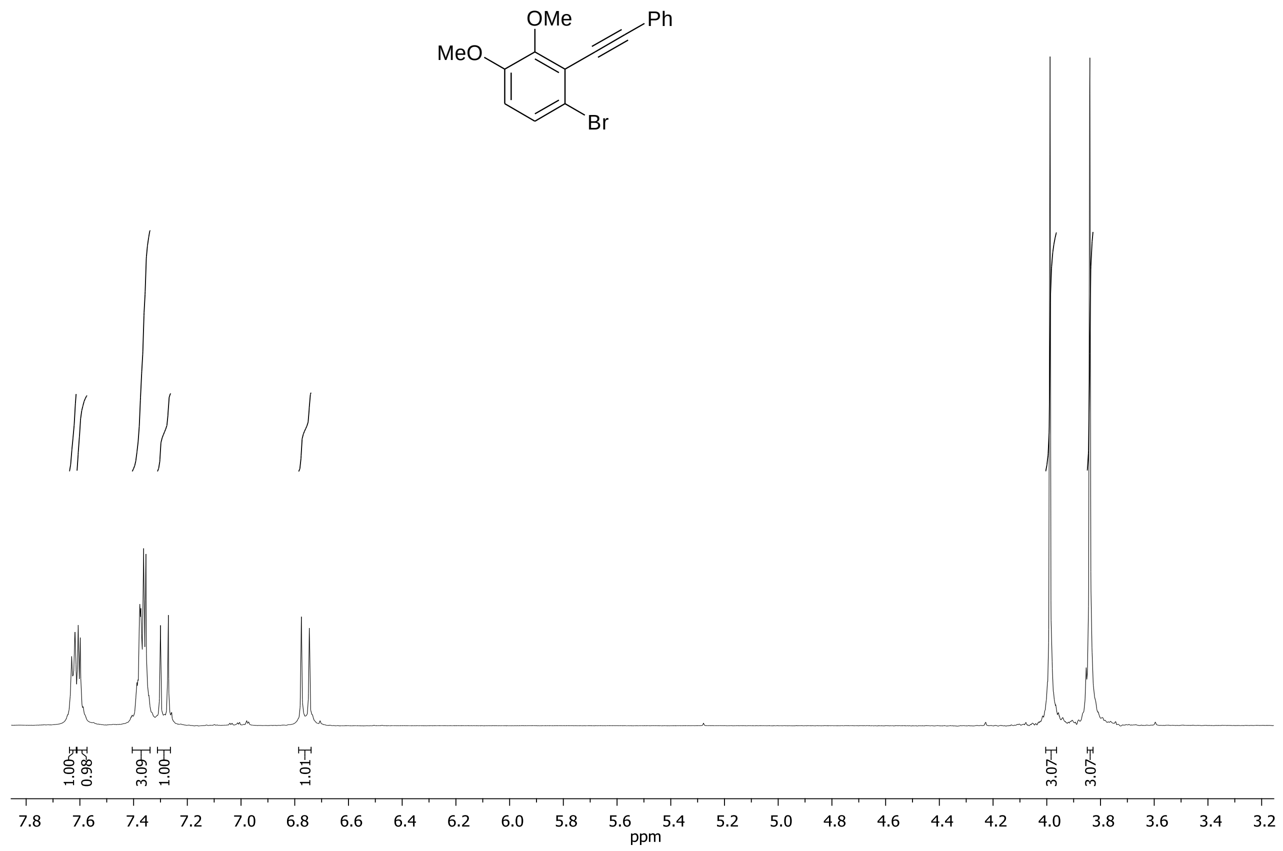

^{13}C NMR (75.4 MHz, $CDCl_3$):

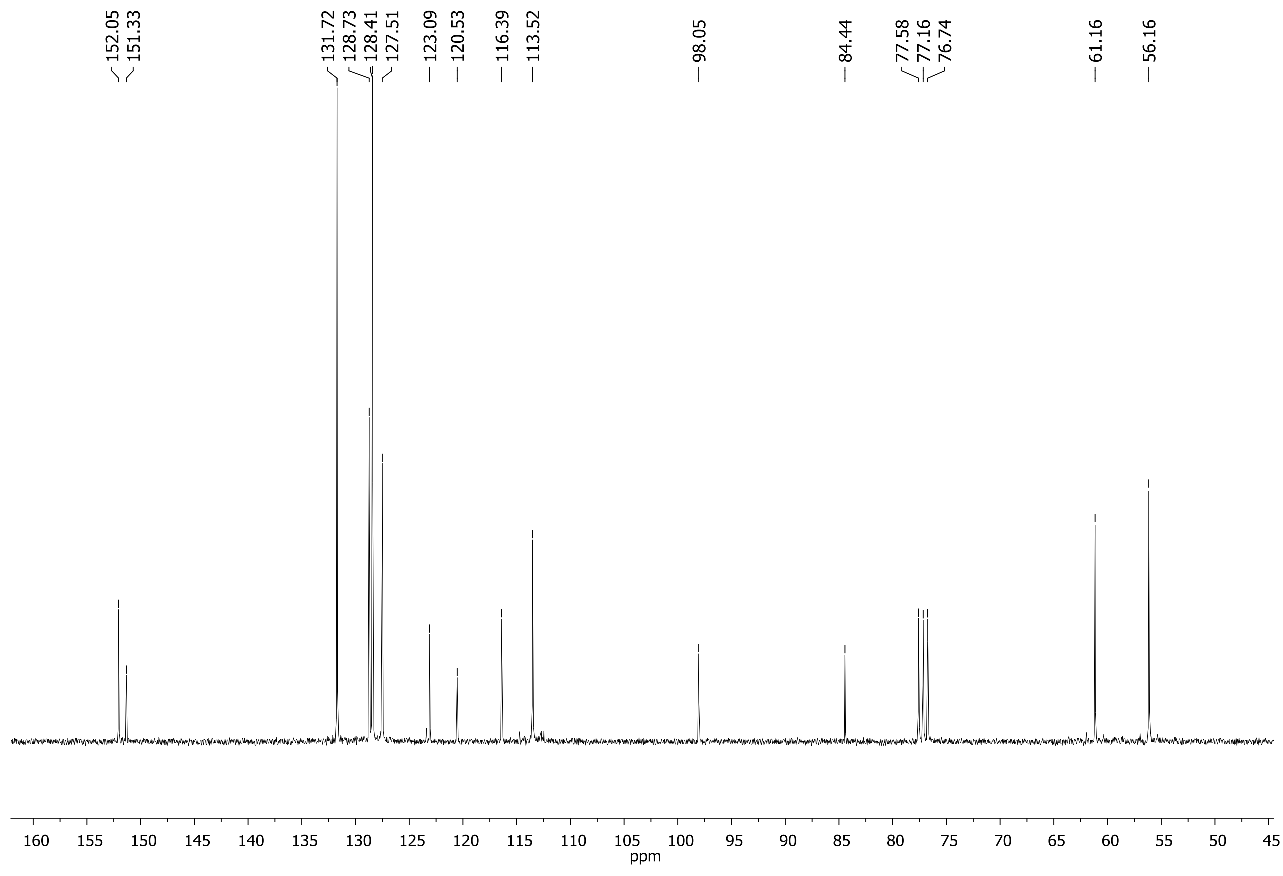

^{1}H NMR (300 MHz, $CDCl_3$) (6a):

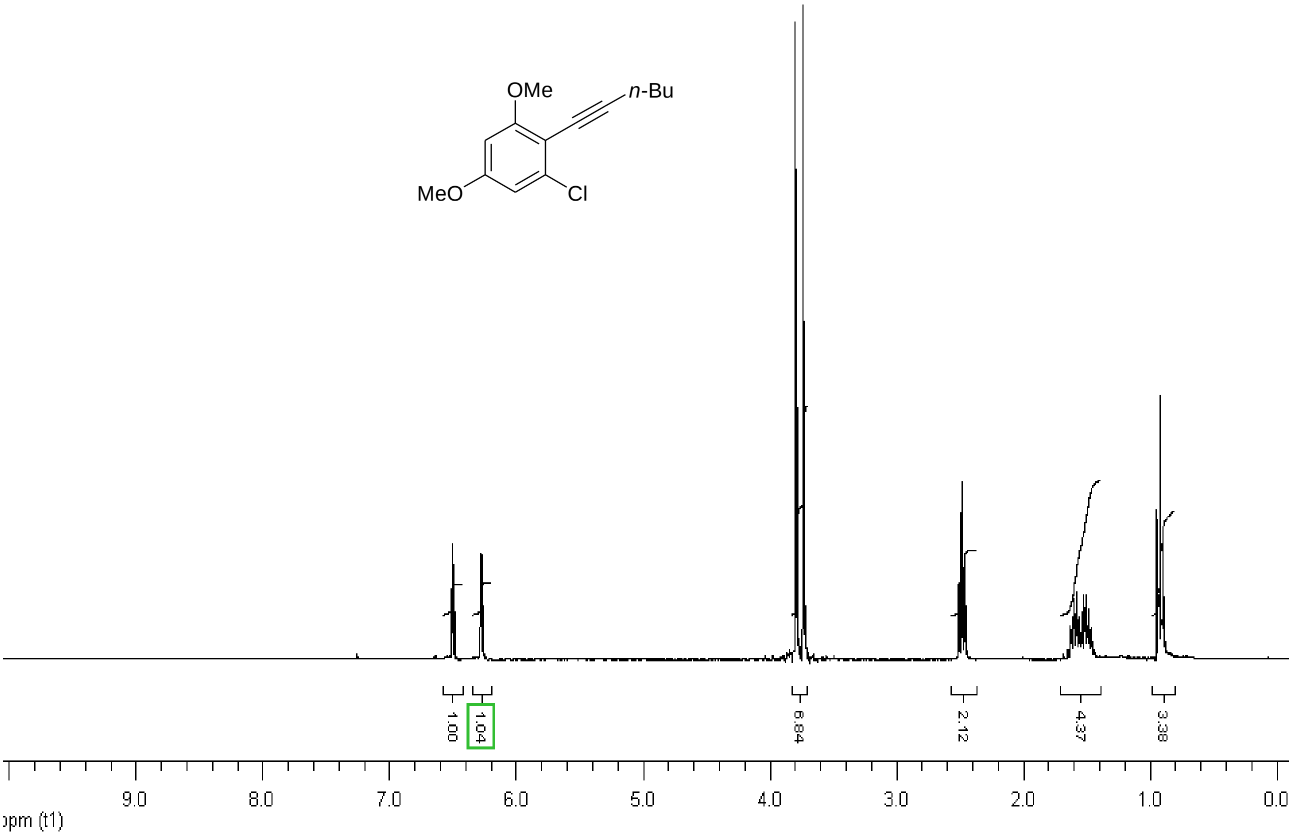

^{13}C NMR (75.4 MHz, $CDCl_3$):

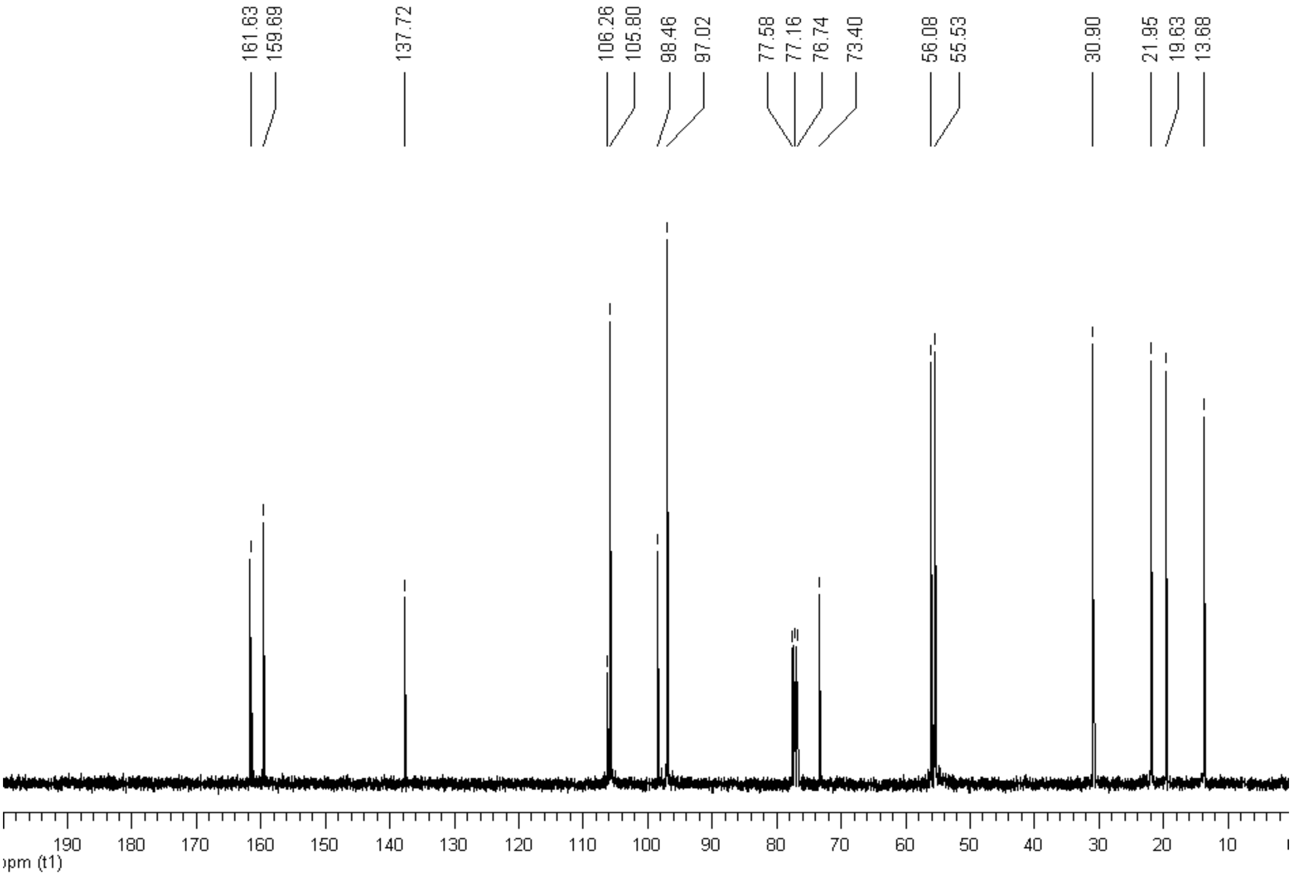

^{1}H NMR (300 MHz, $CDCl_3$) (6b):

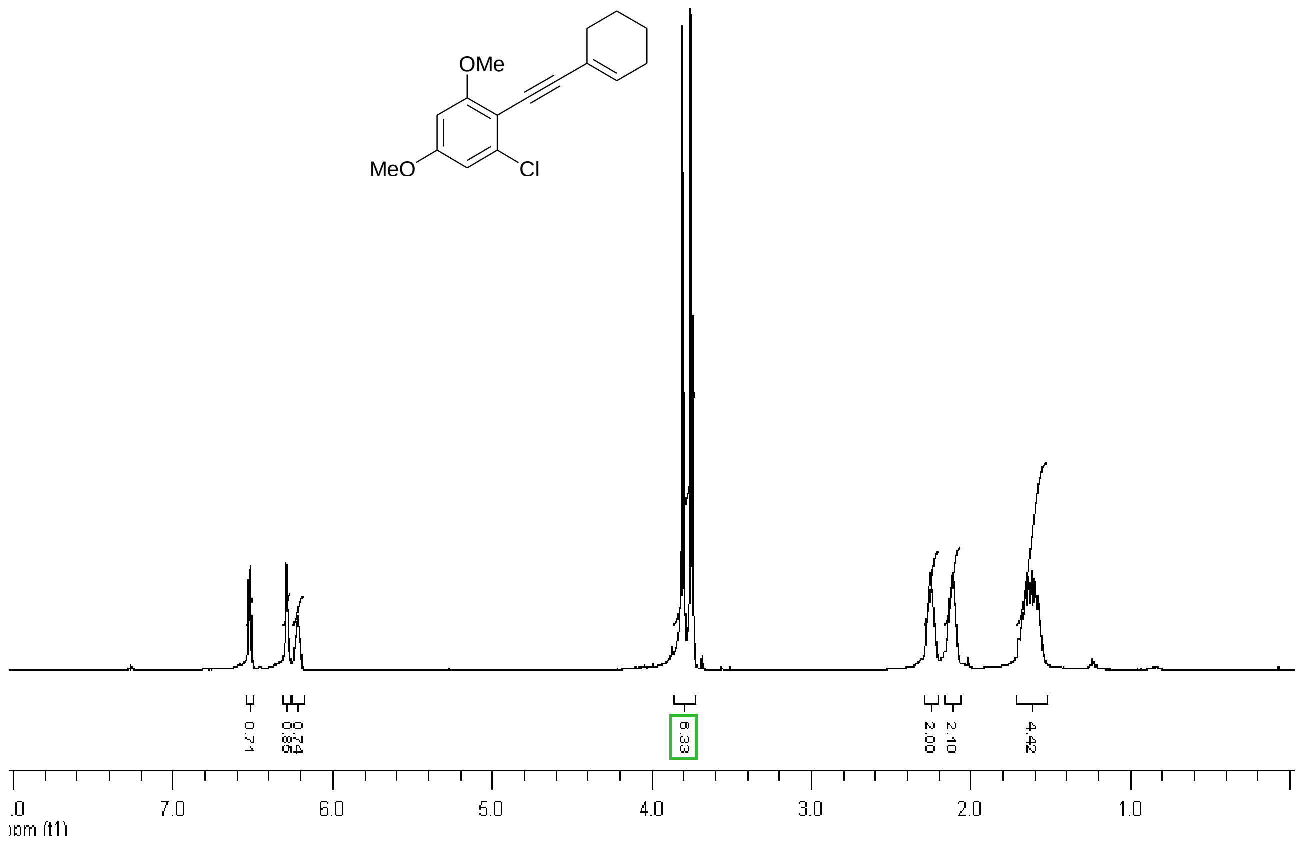

^{13}C NMR (75.4 MHz, $CDCl_3$):

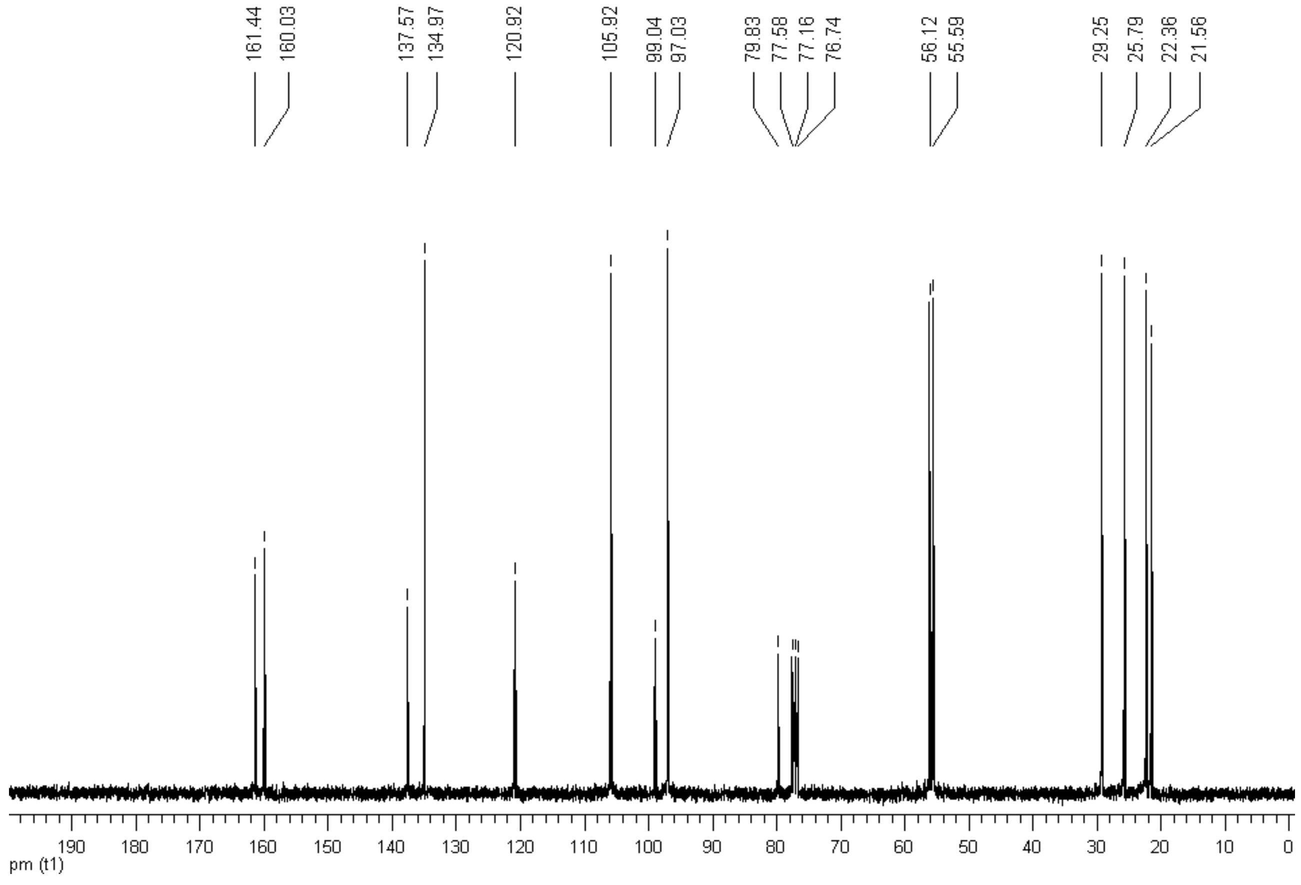

^{1}H NMR (300 MHz, $CDCl_3$) (6c):

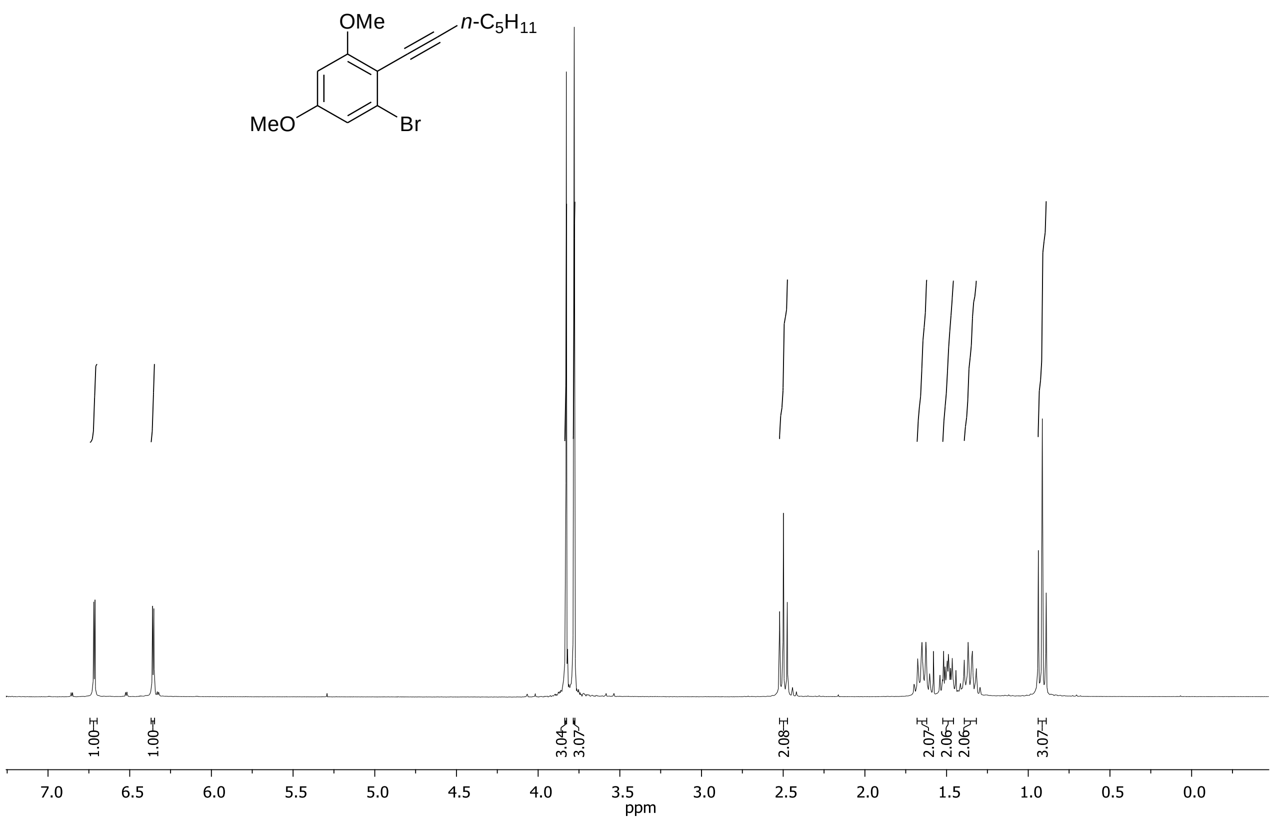

^{13}C NMR (75.4 MHz, $CDCl_3$):

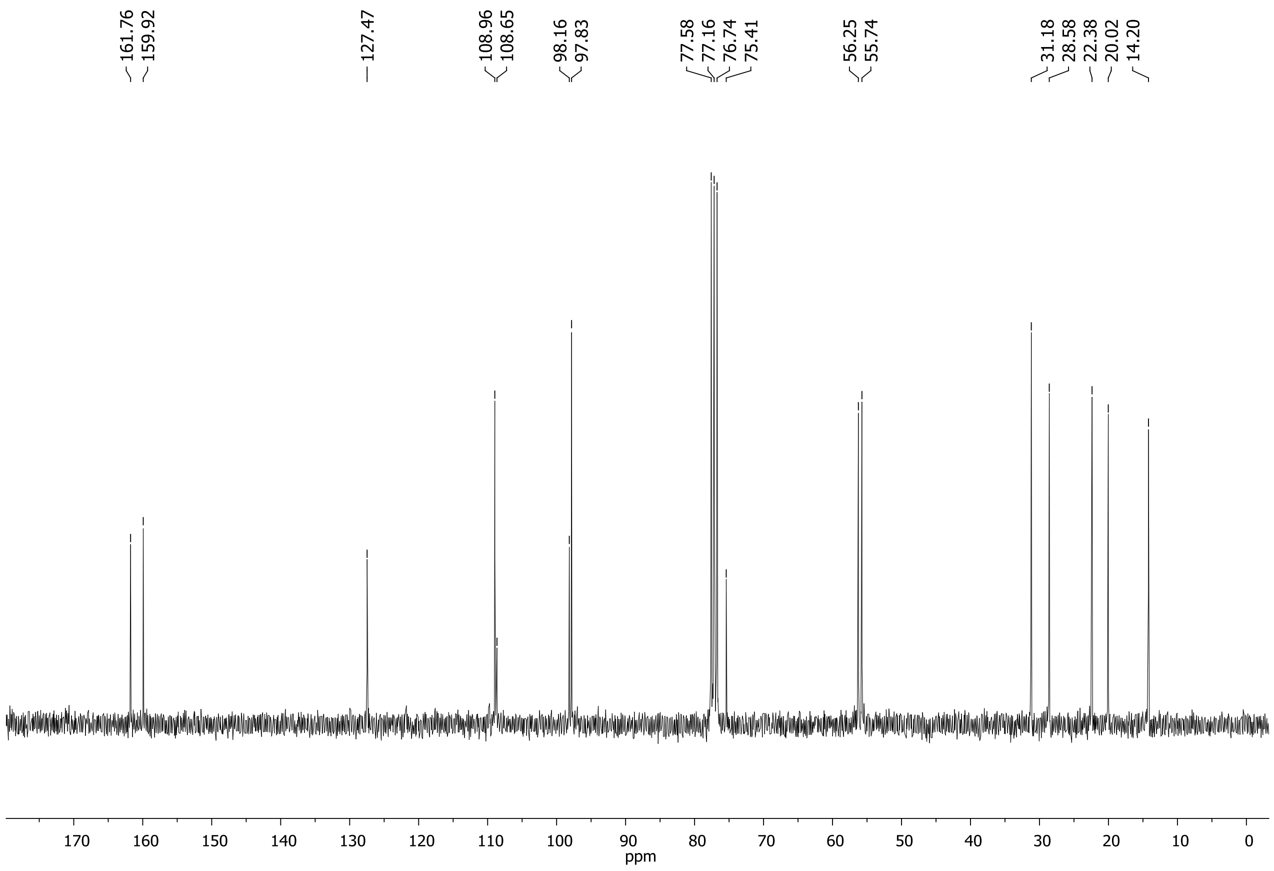

^{1}H NMR (300 MHz, $CDCl_3$) (6d):

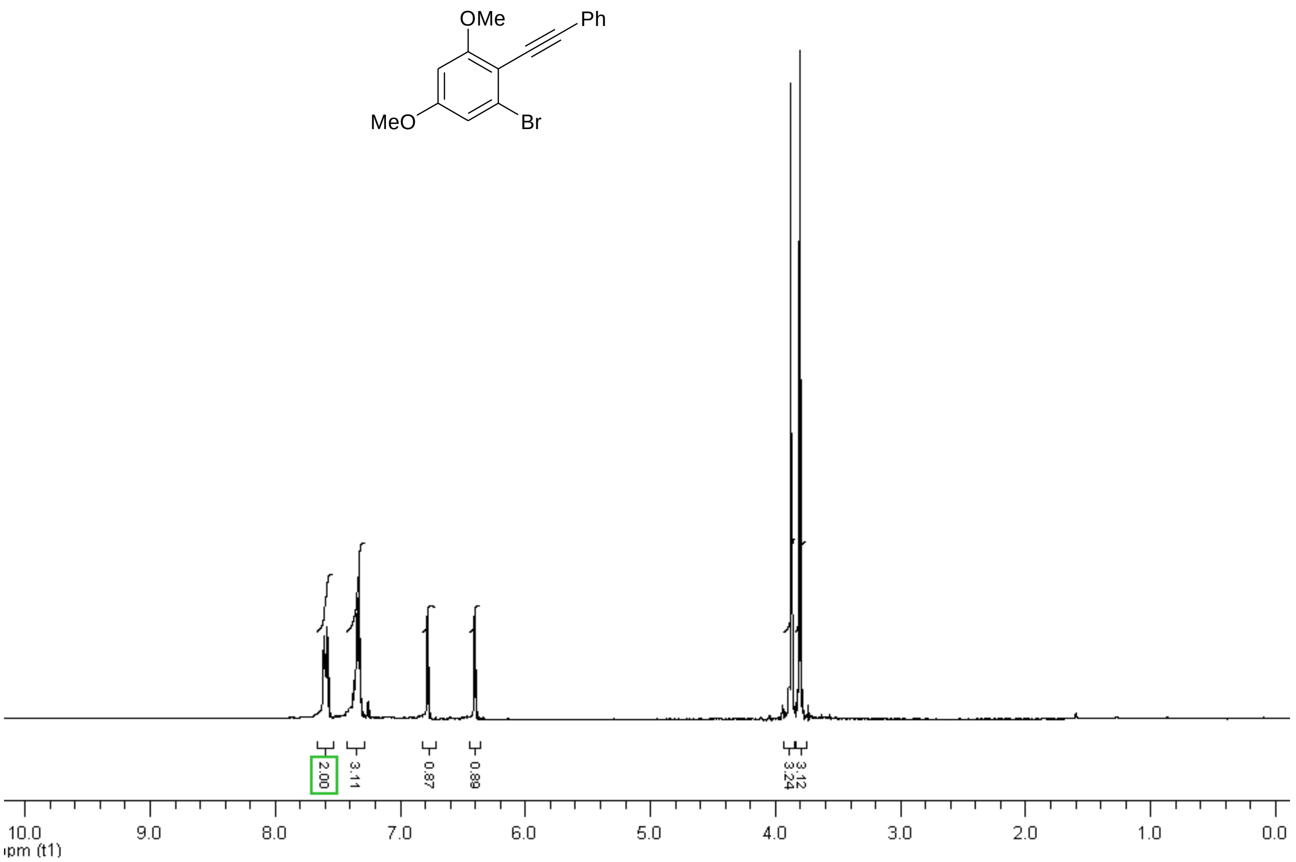

^{13}C NMR (75.4 MHz, $CDCl_3$):

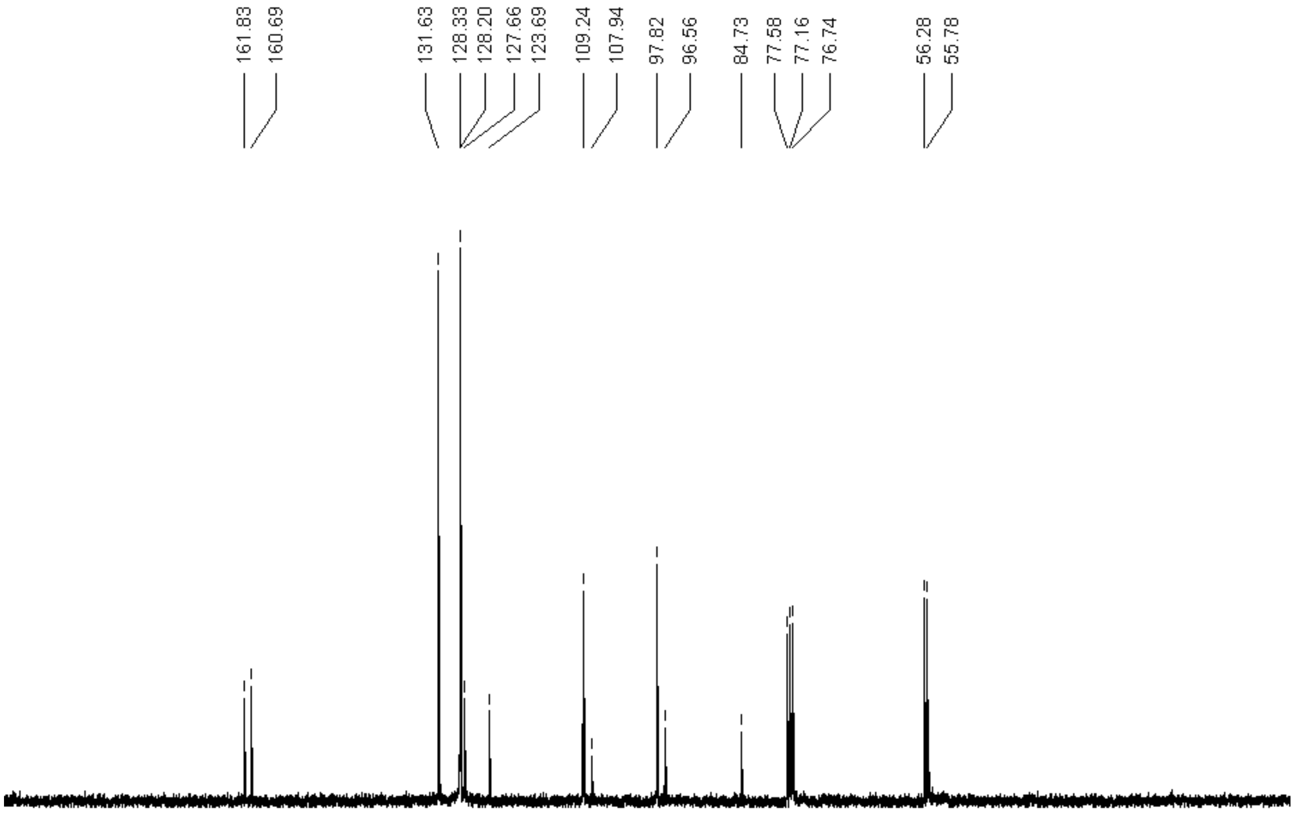

^{1}H NMR (300 MHz, $CDCl_3$) (6e):

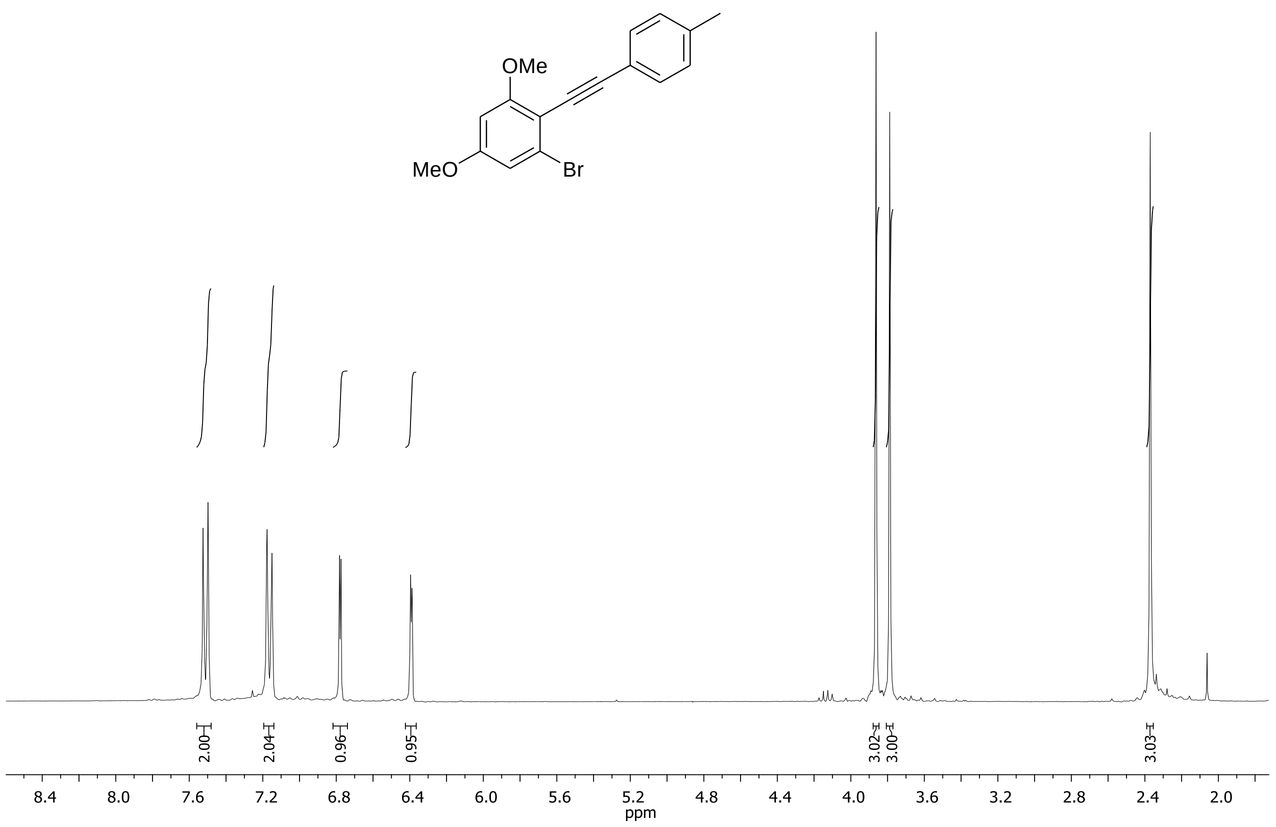

^{13}C NMR (75.4 MHz, $CDCl_3$):

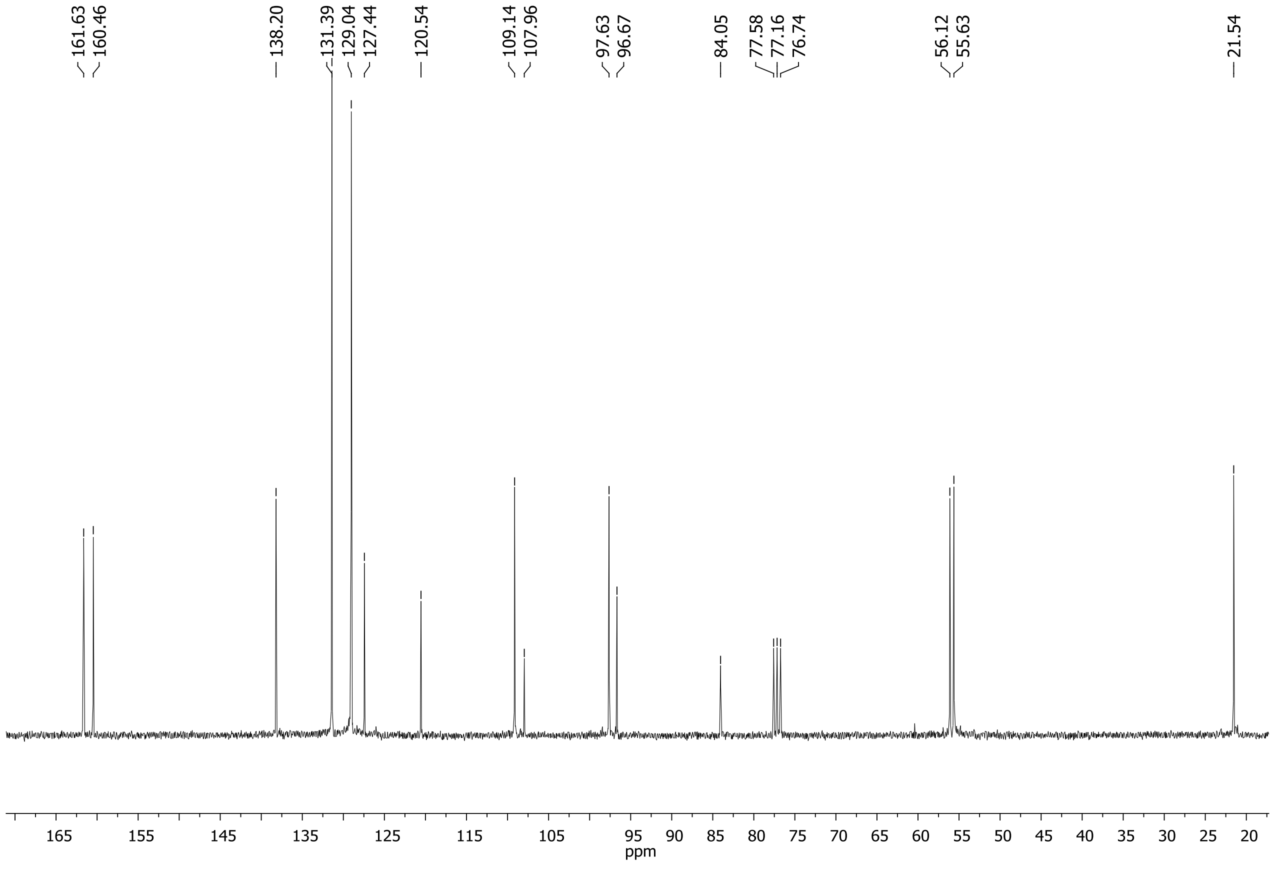

¹H NMR (300 MHz, $CDCl_3$) (7a):

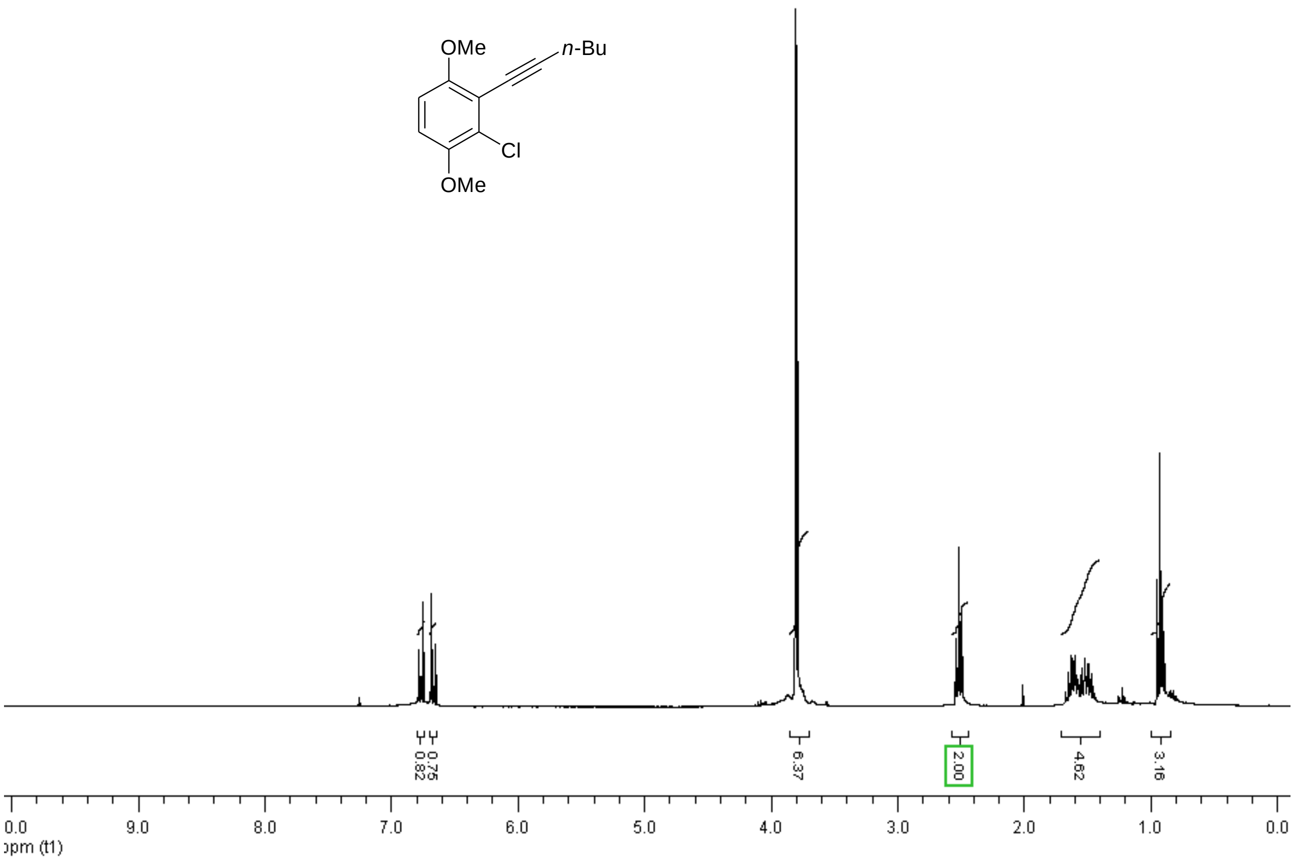

¹³C NMR (75.4 MHz, $CDCl_3$):

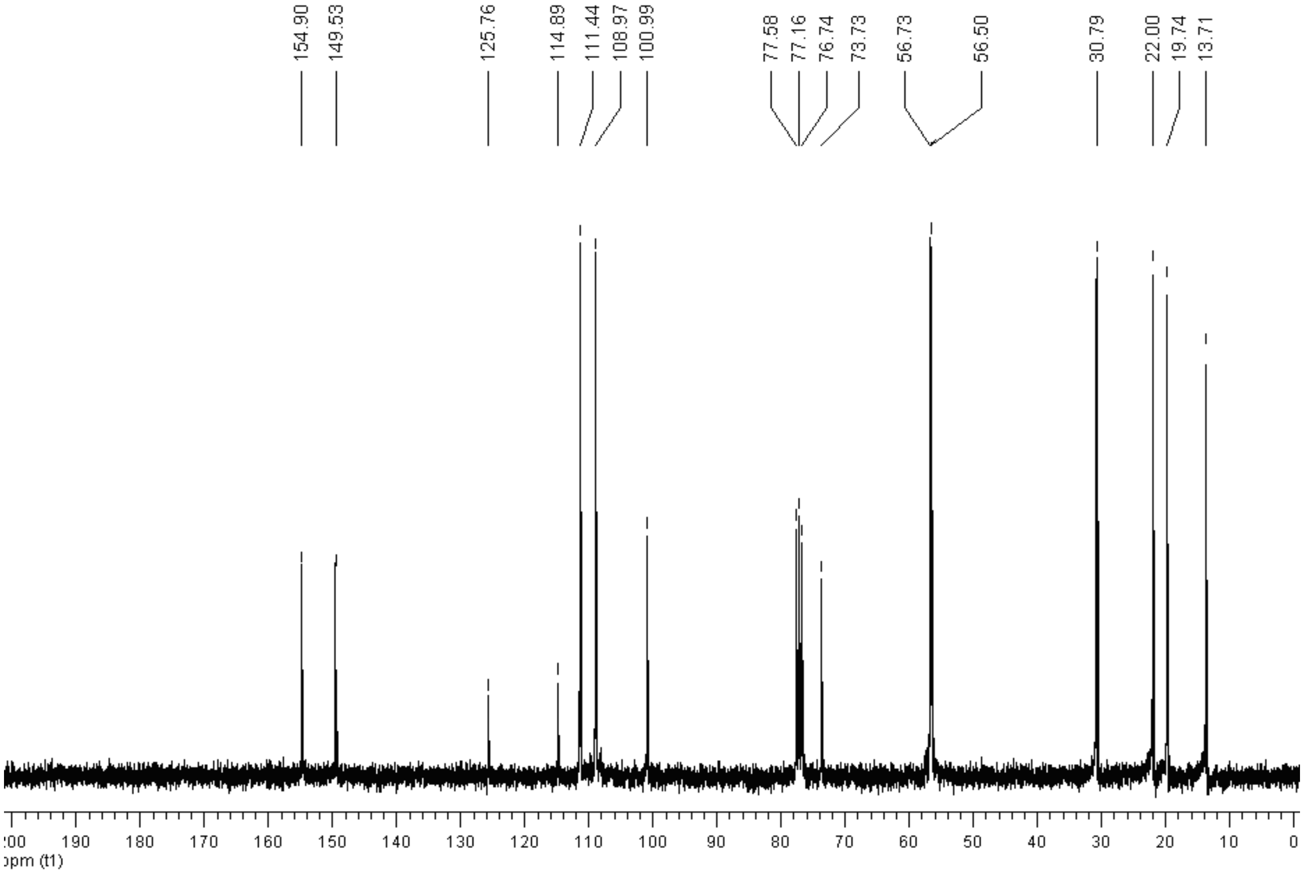

^{1}H NMR (300 MHz, $CDCl_3$) (7b):

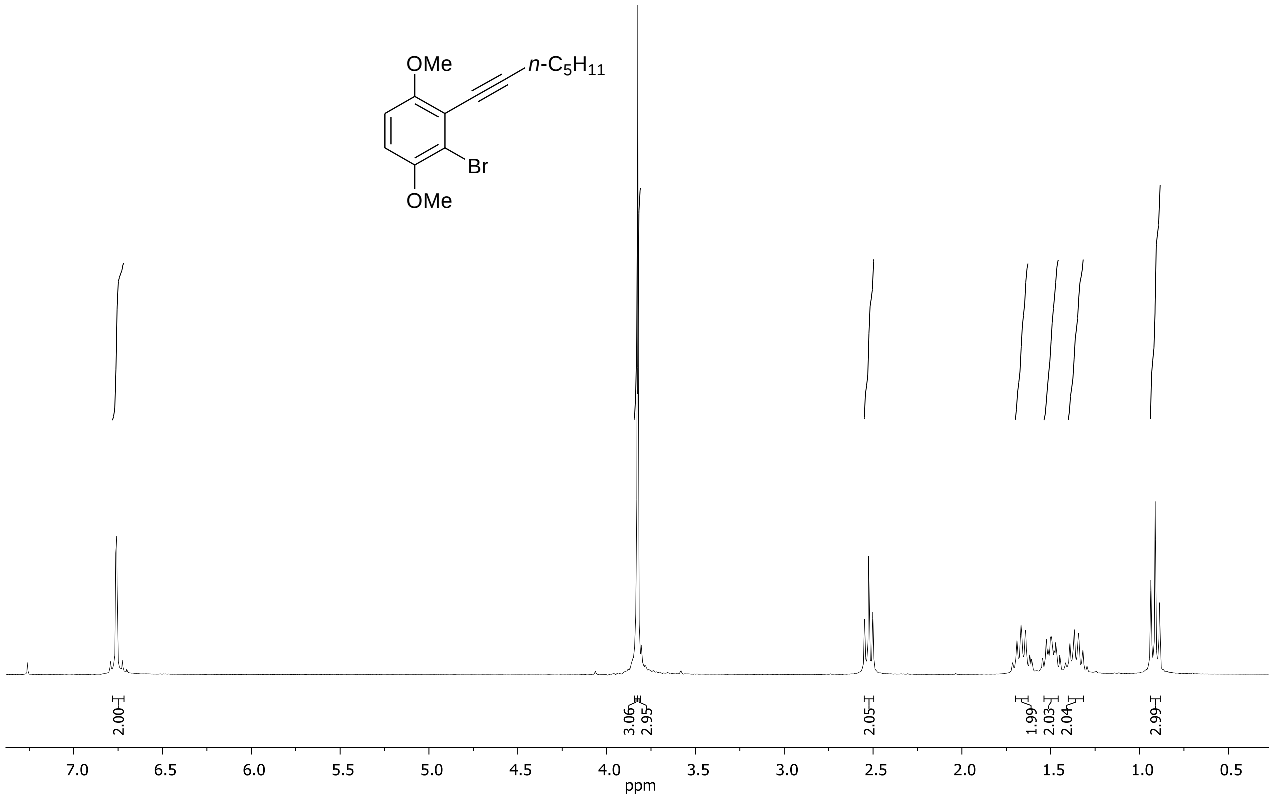

^{13}C NMR (75.4 MHz, $CDCl_3$):

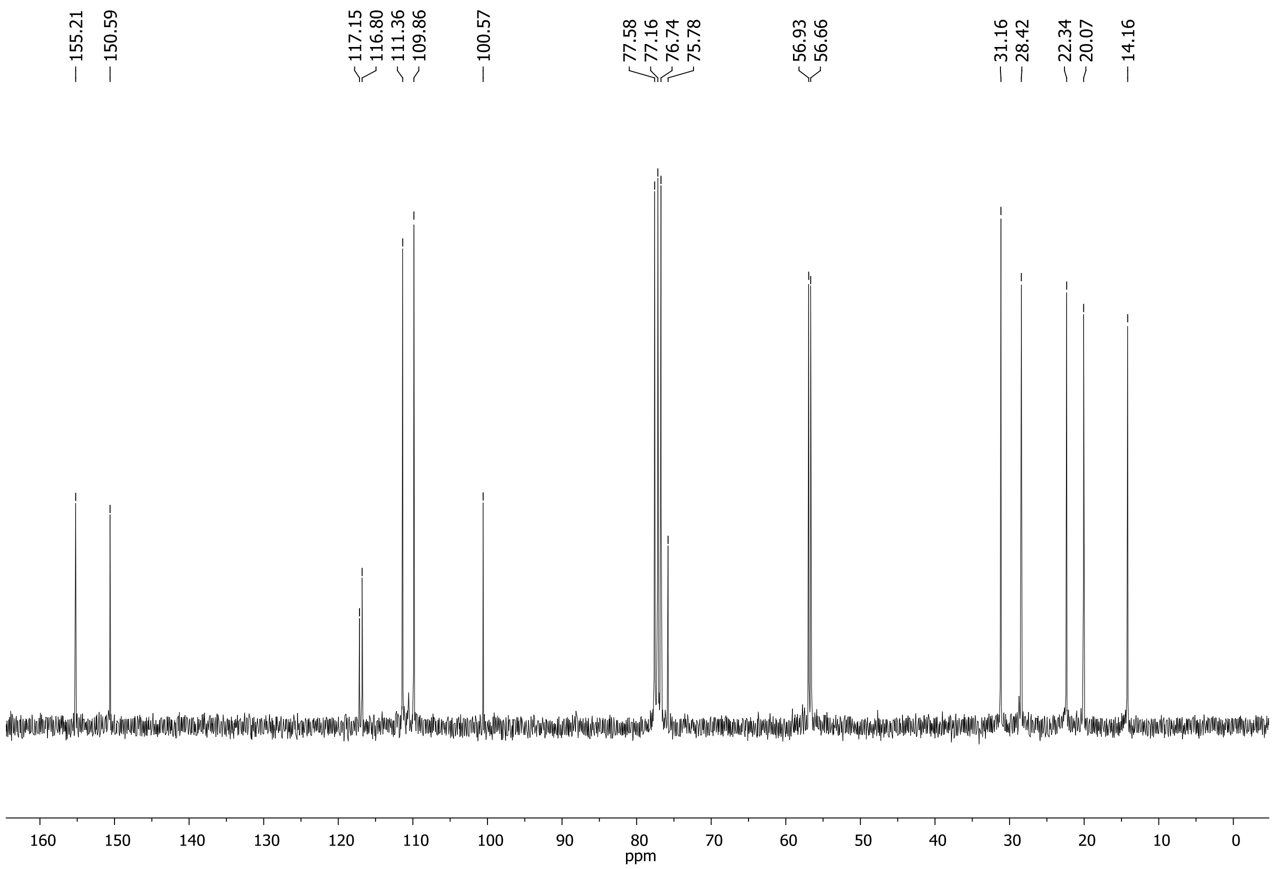

^{1}H NMR (300 MHz, $CDCl_3$) (7c):

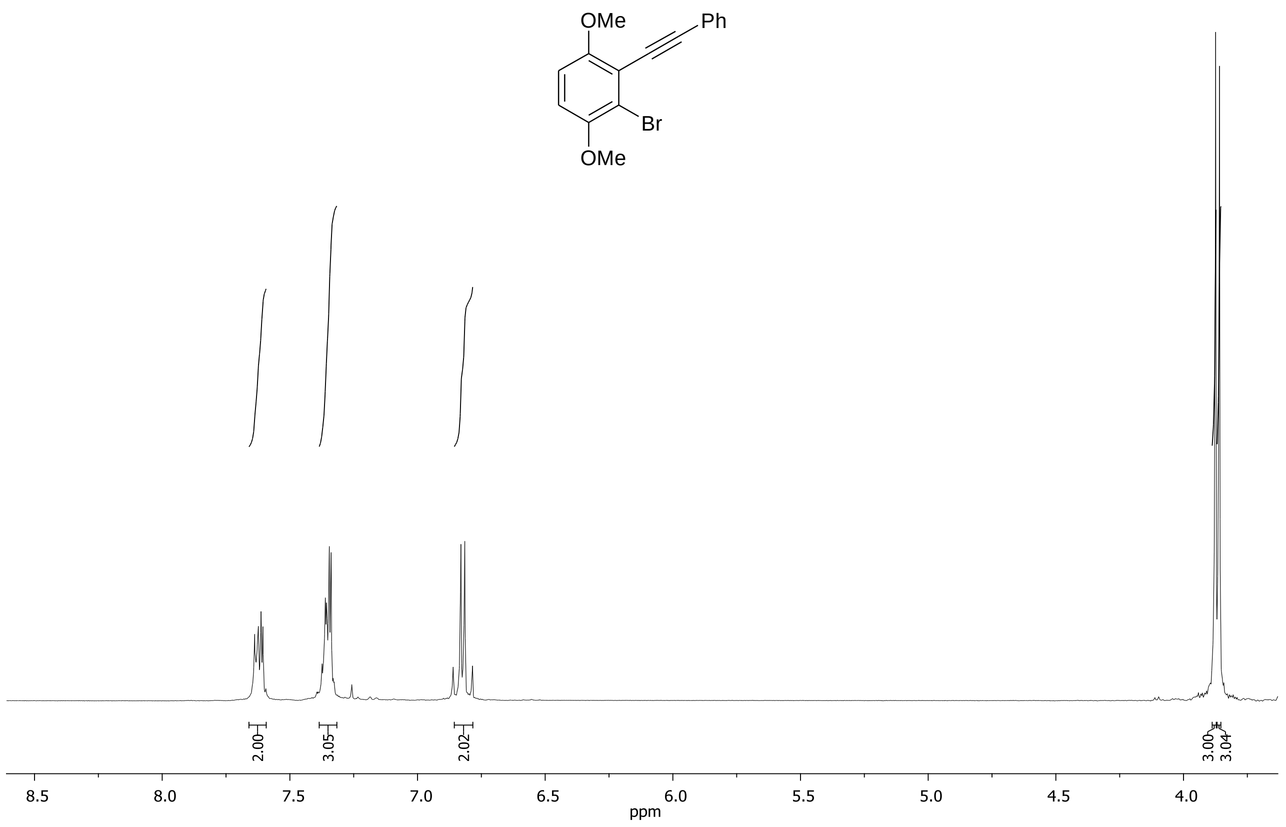

^{13}C NMR (75.4 MHz, $CDCl_3$):

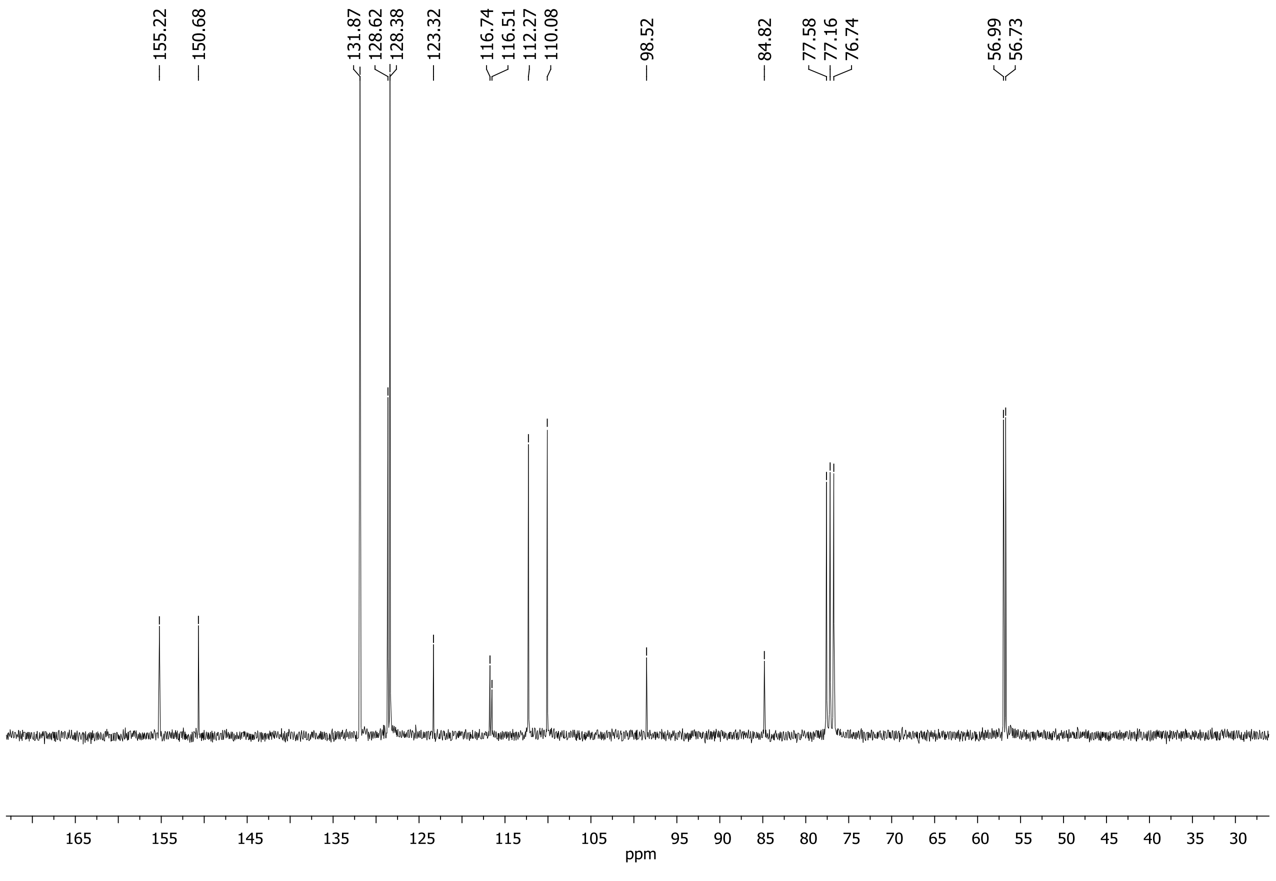

^{1}H NMR (300 MHz, $CDCl_3$) (7d):

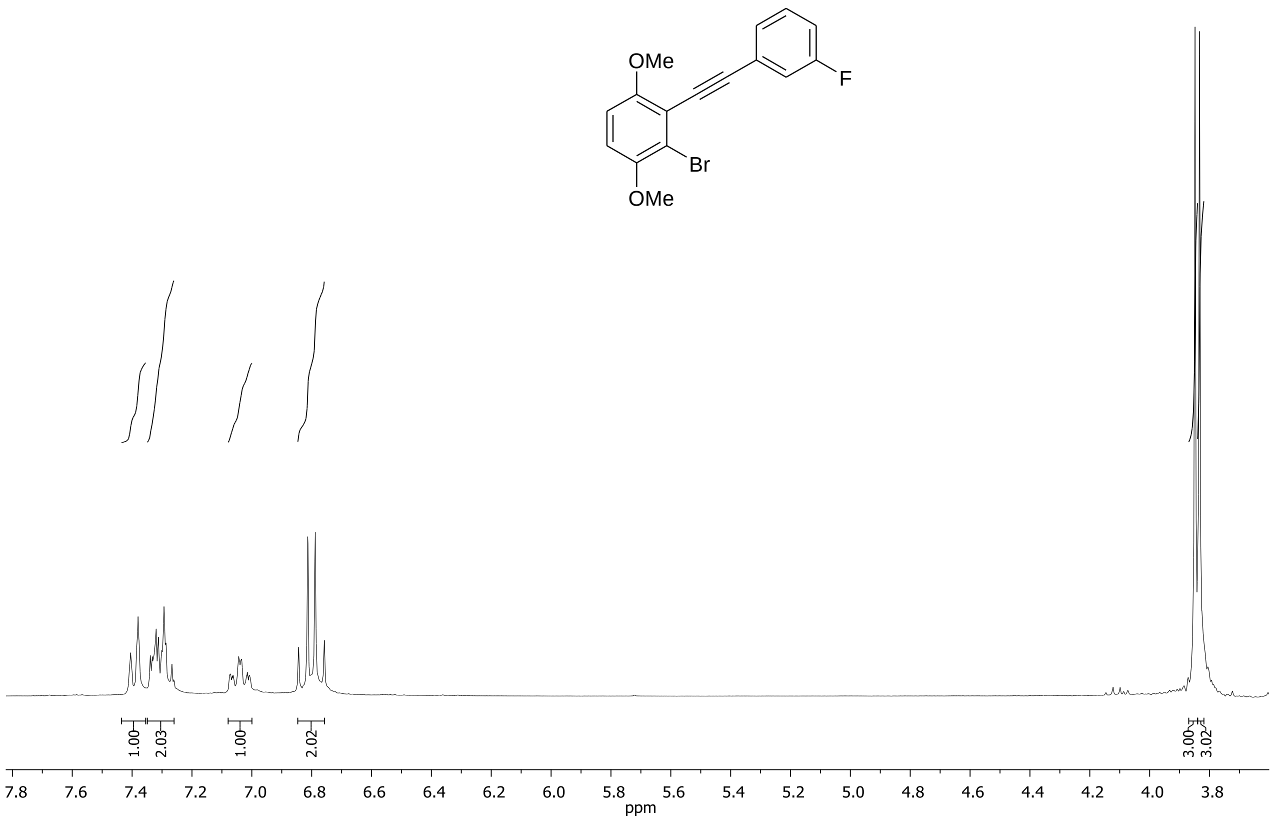

^{13}C NMR (75.4 MHz, $CDCl_3$):

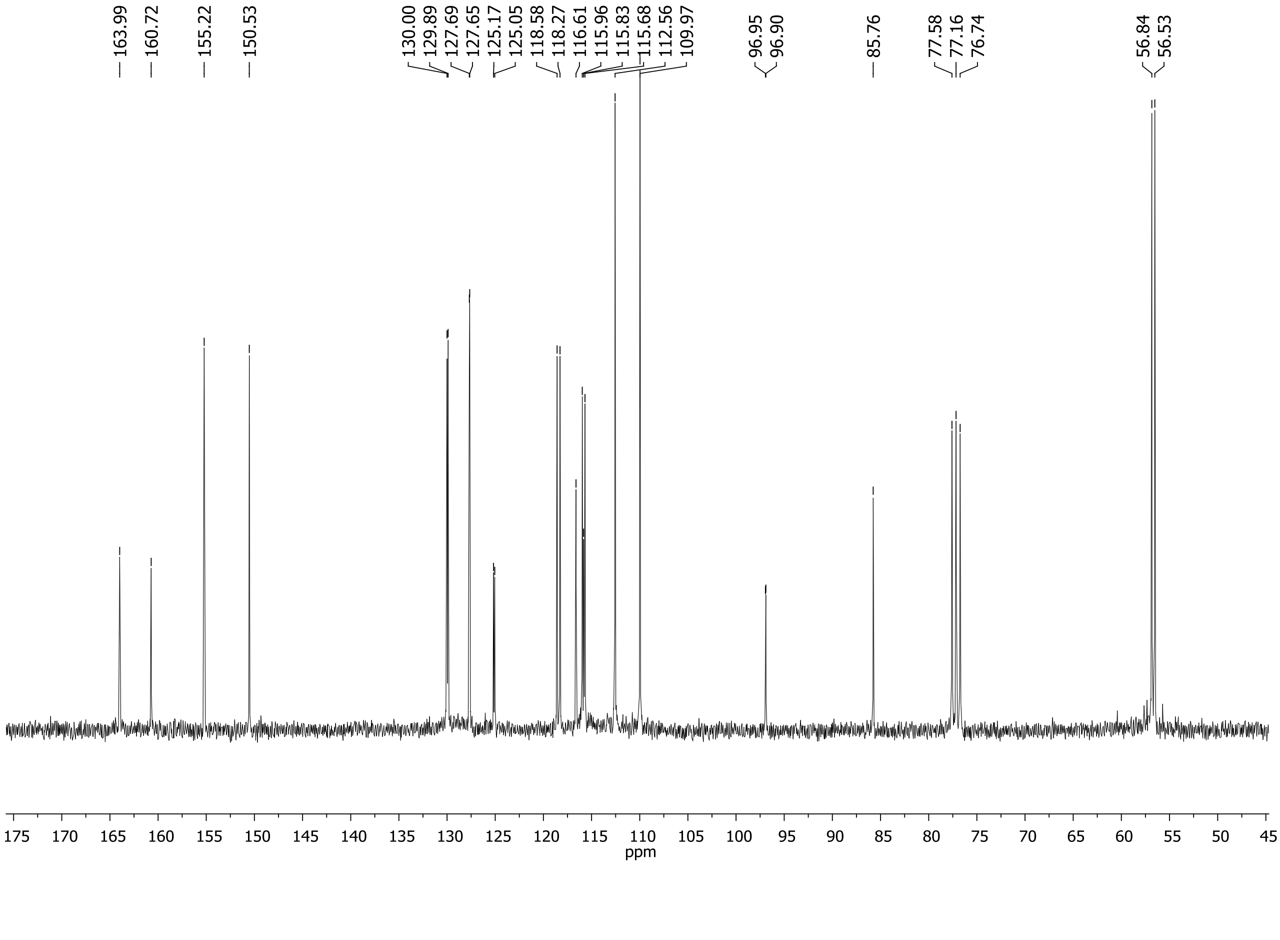

^{1}H NMR (300 MHz, $CDCl_3$) (8a):

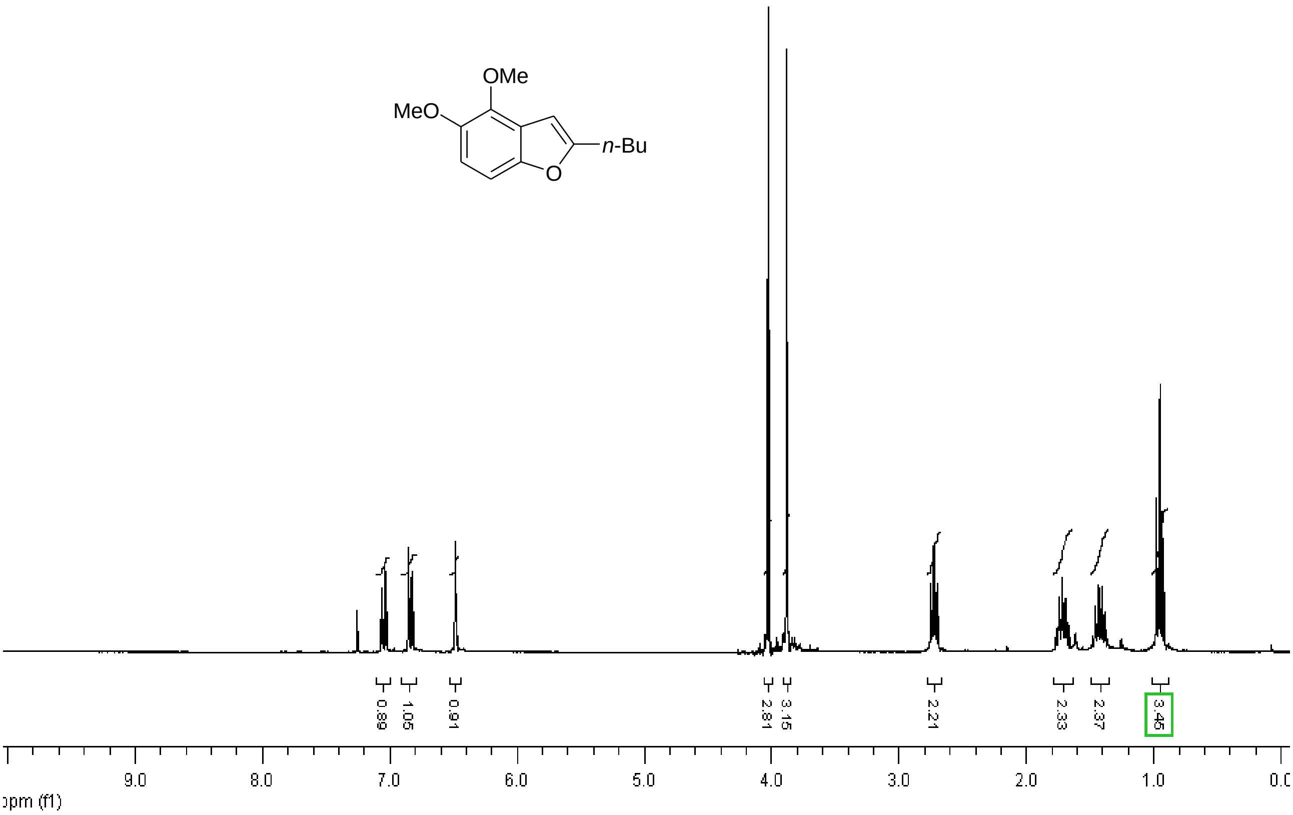

^{13}C NMR (75.4 MHz, $CDCl_3$):

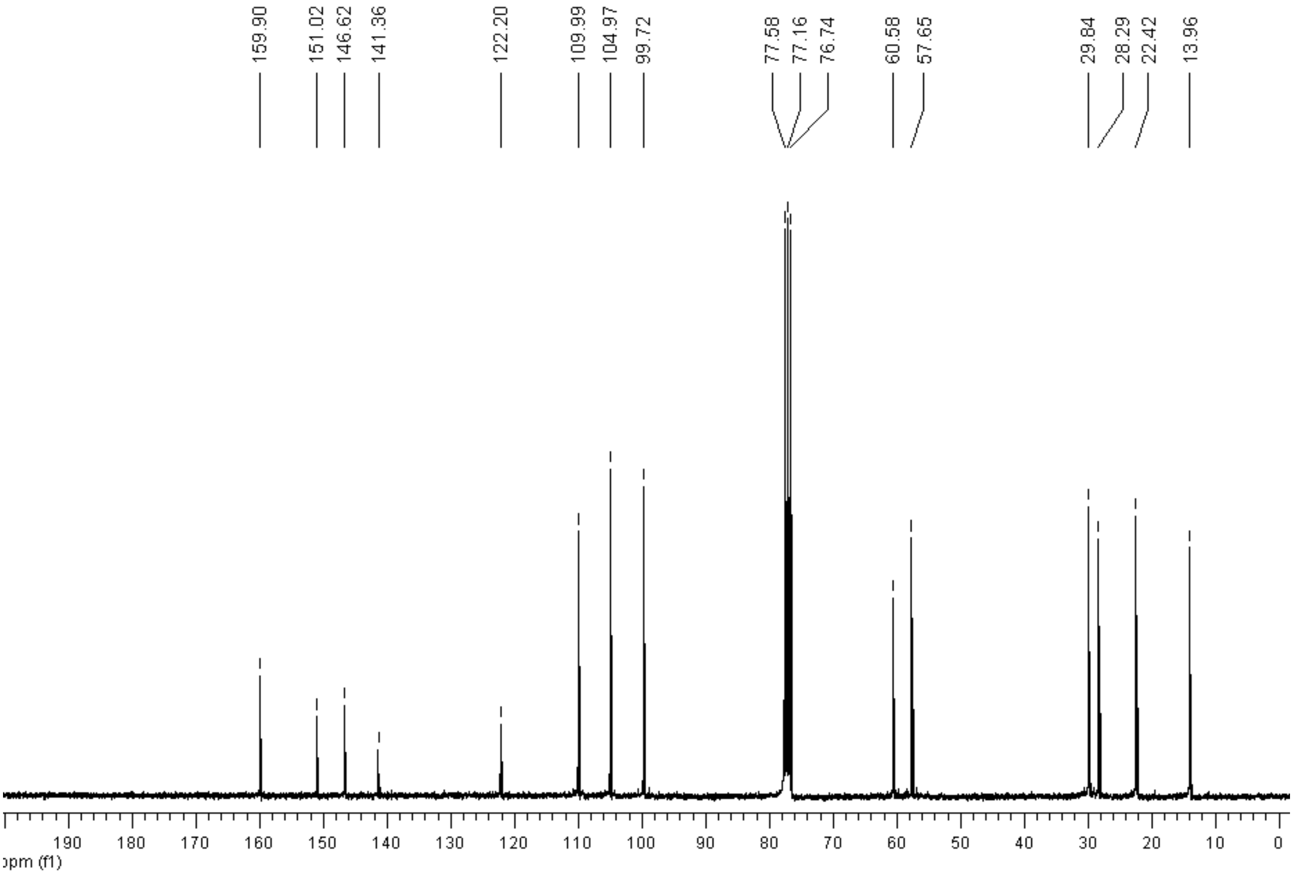

^{1}H NMR (300 MHz, $CDCl_3$) (8b):

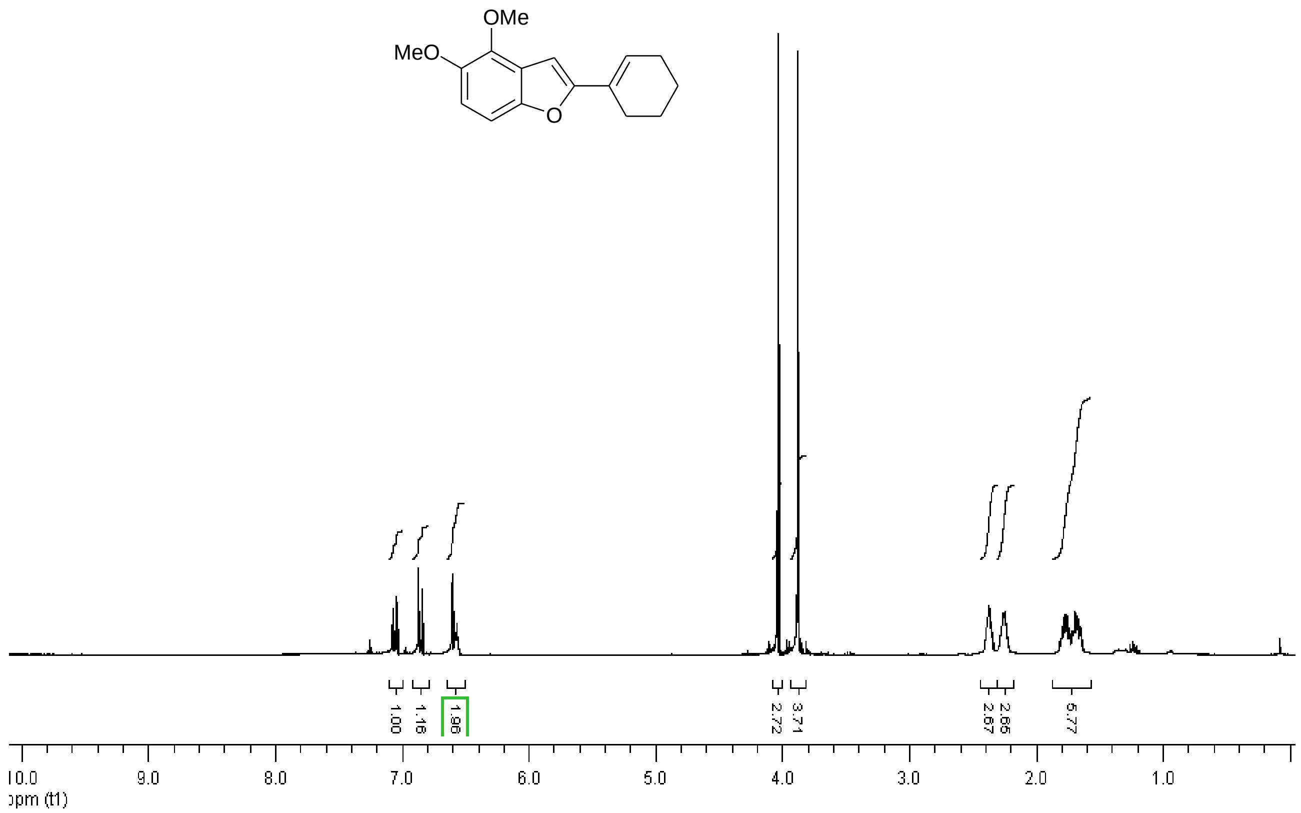

^{13}C NMR (75.4 MHz, $CDCl_3$):

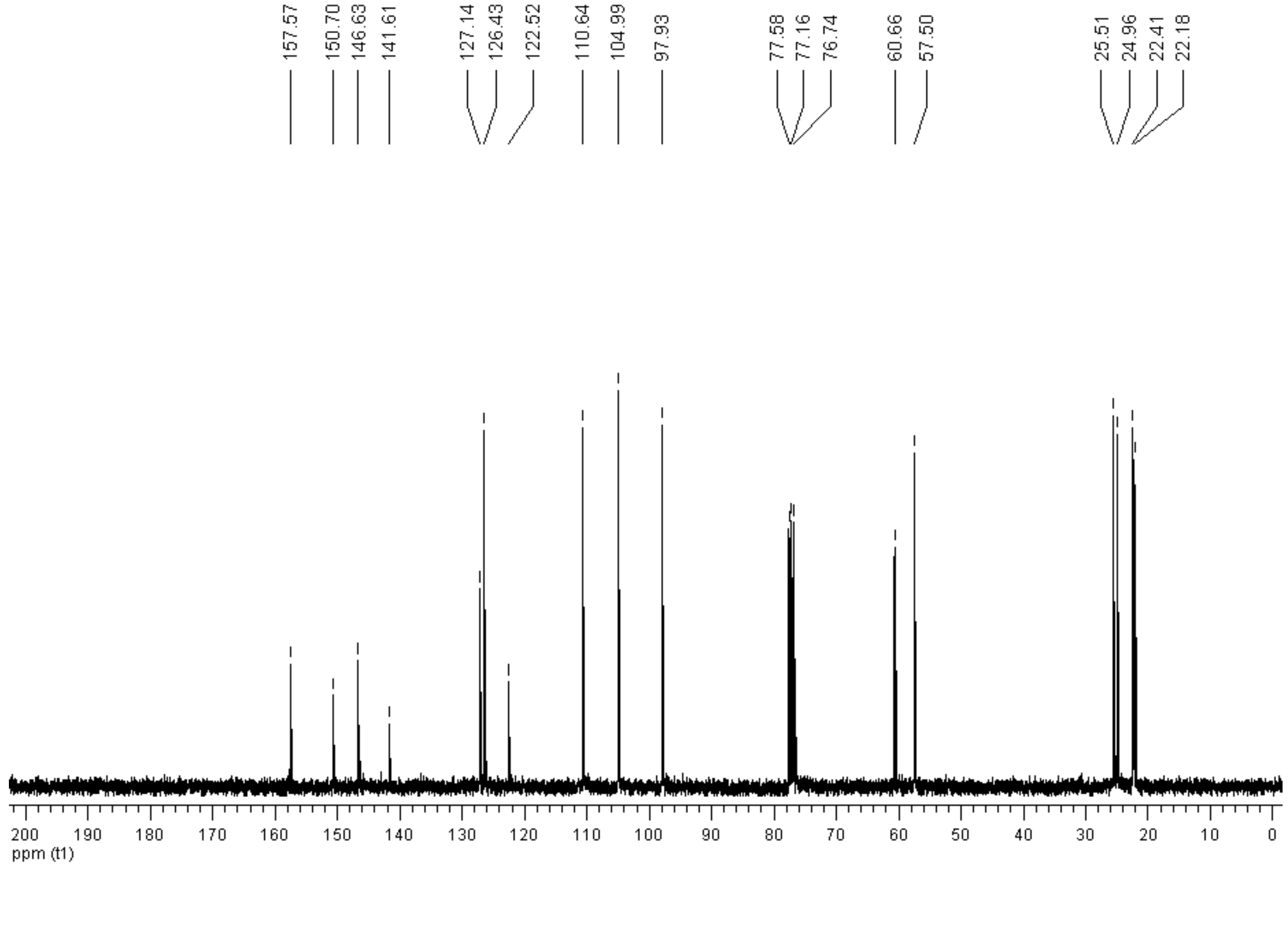

^{1}H NMR (300 MHz, $CDCl_3$) (8c):

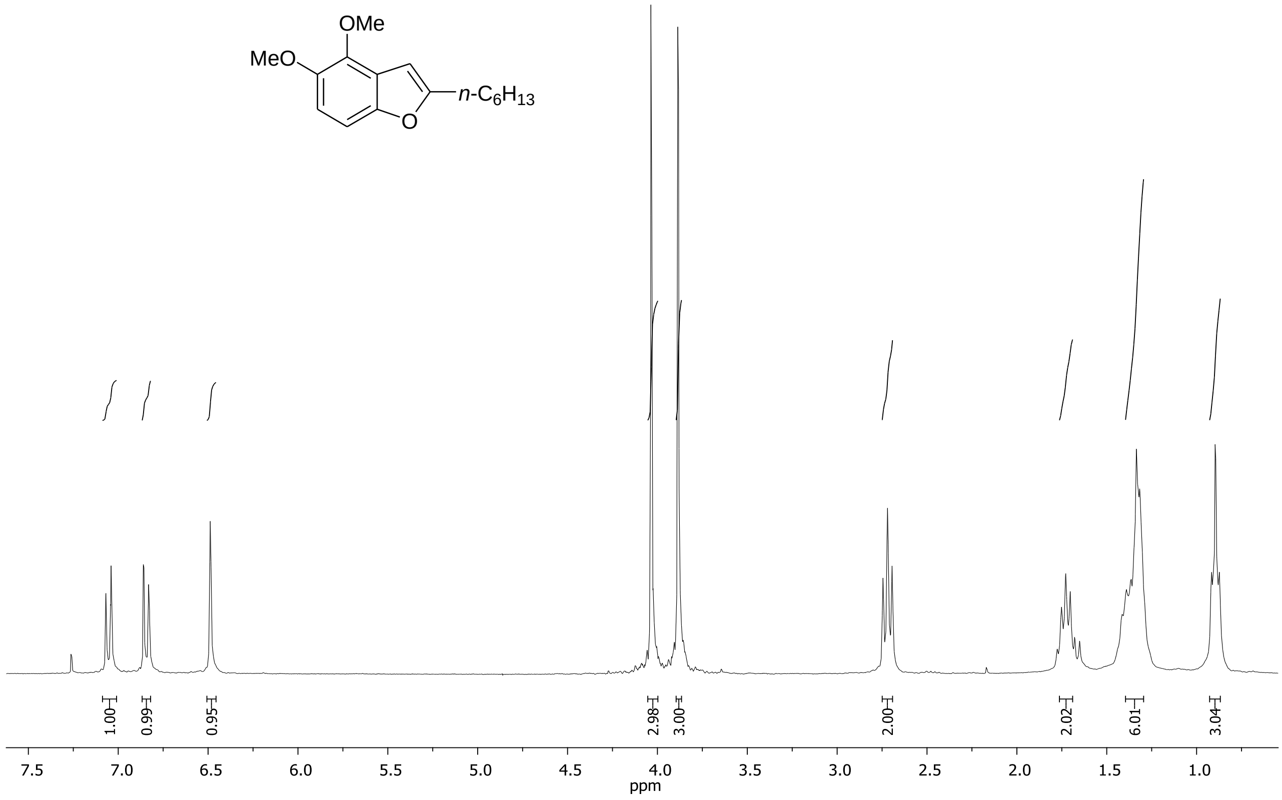

^{13}C NMR (75.4 MHz, $CDCl_3$):

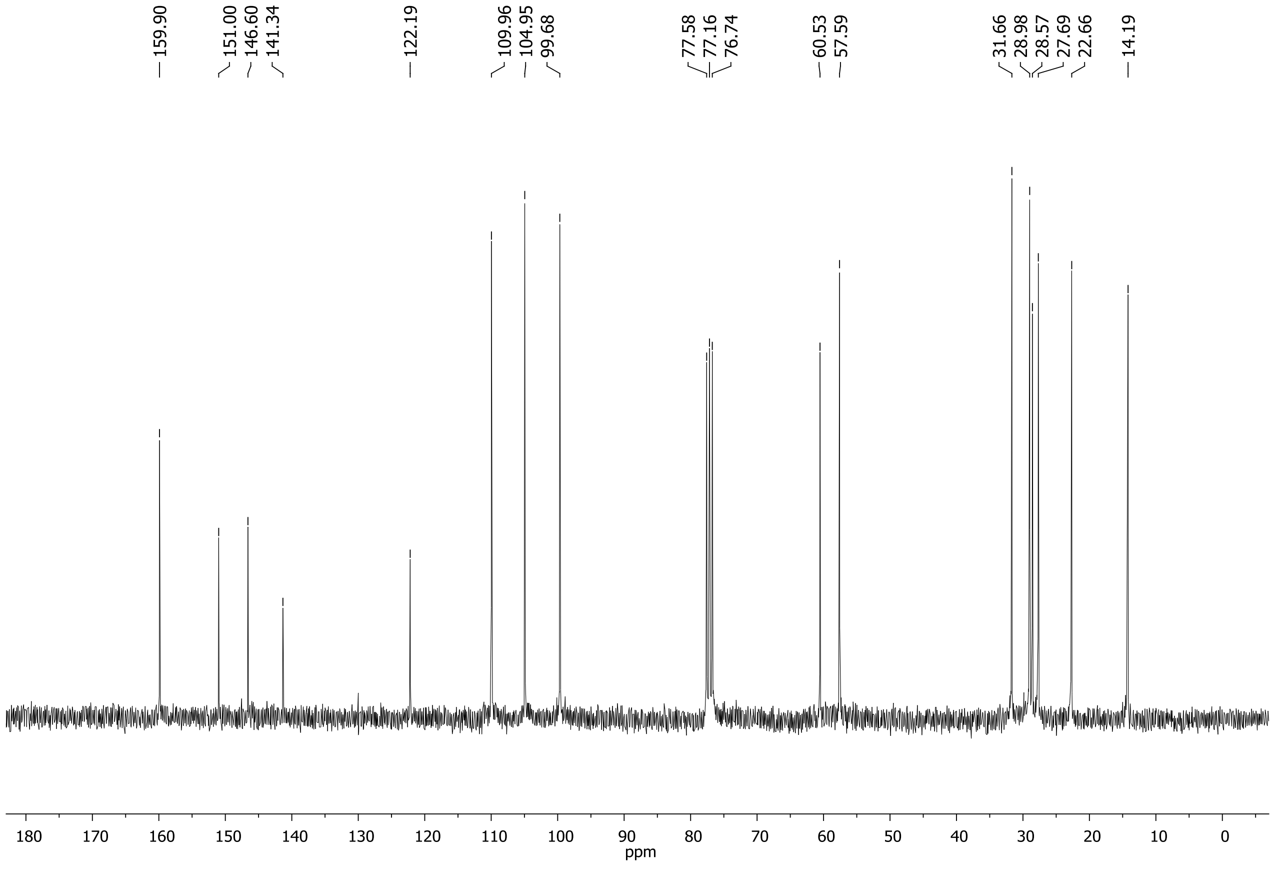

^{1}H NMR (300 MHz, $CDCl_3$) (8d):

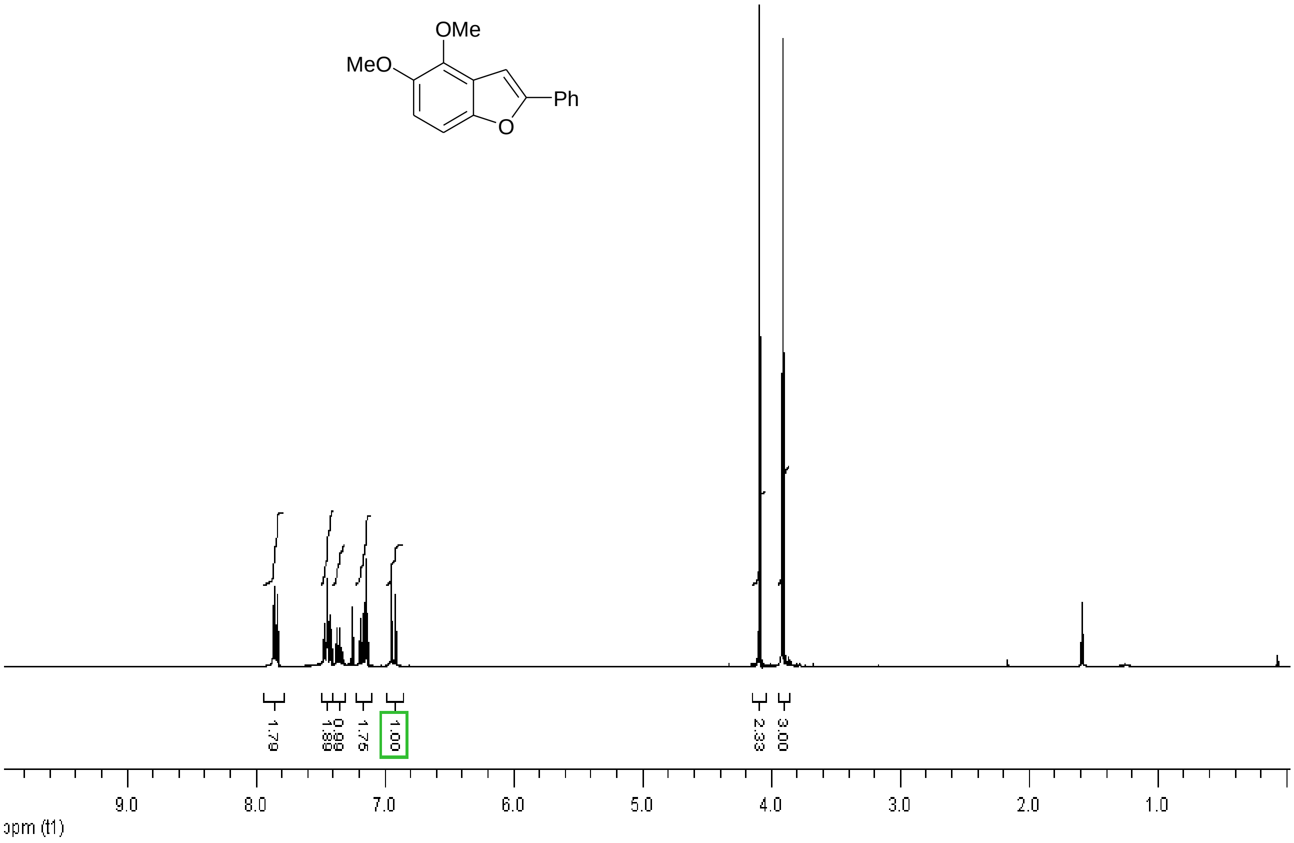

^{13}C NMR (75.4 MHz, $CDCl_3$):

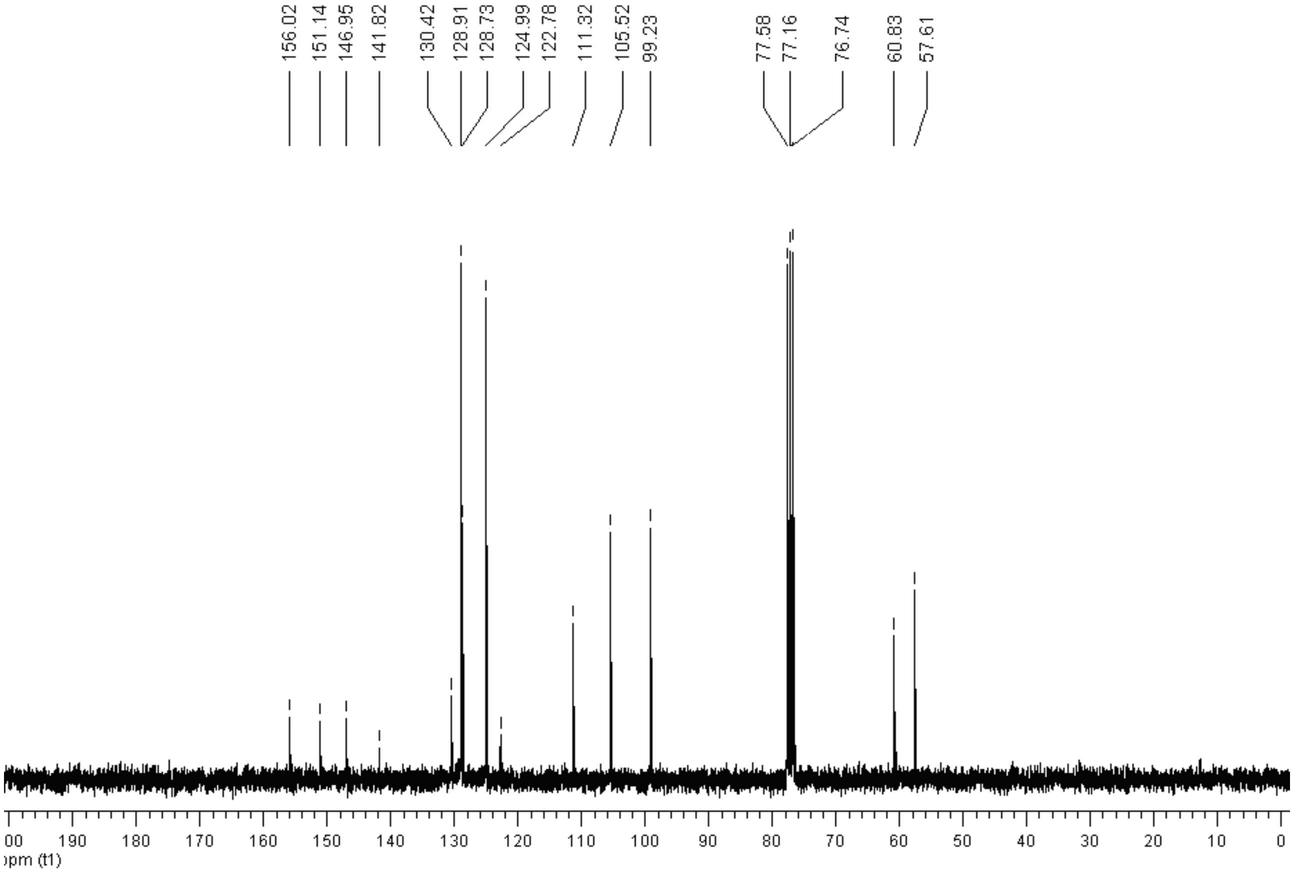

^{1}H NMR (300 MHz, $CDCl_3$) (9a):

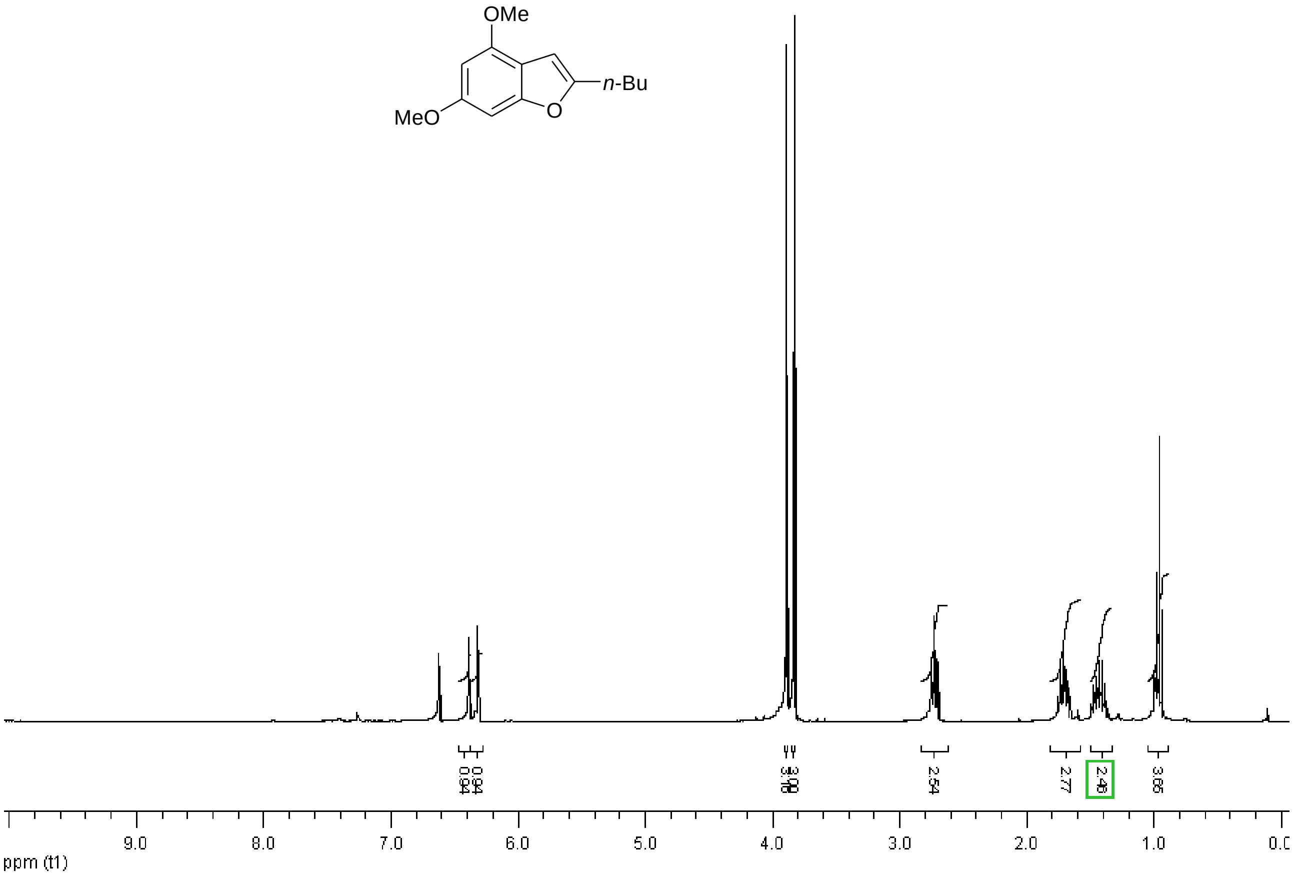

^{13}C NMR (75.4 MHz, $CDCl_3$):

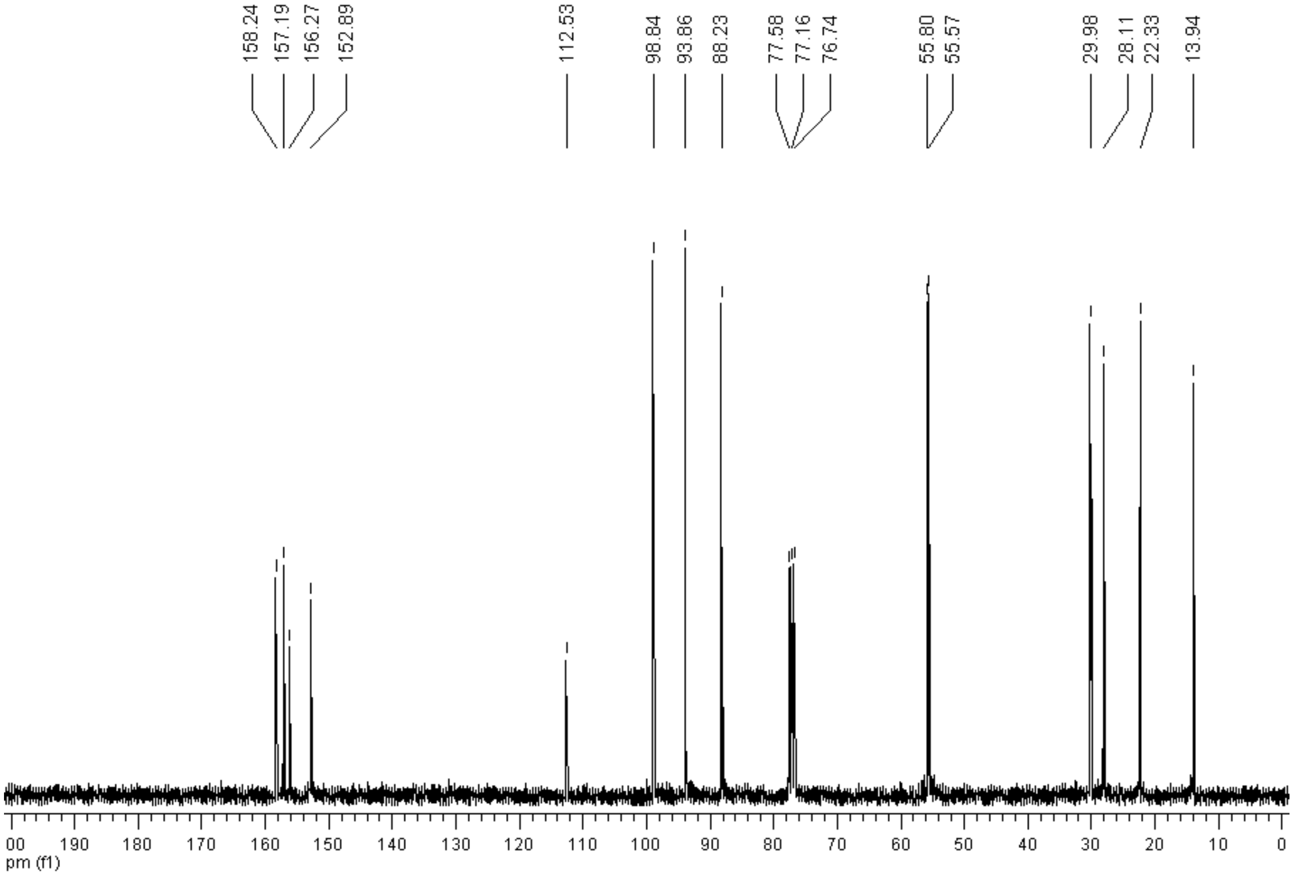

^{1}H NMR (300 MHz, $CDCl_3$) (9b):

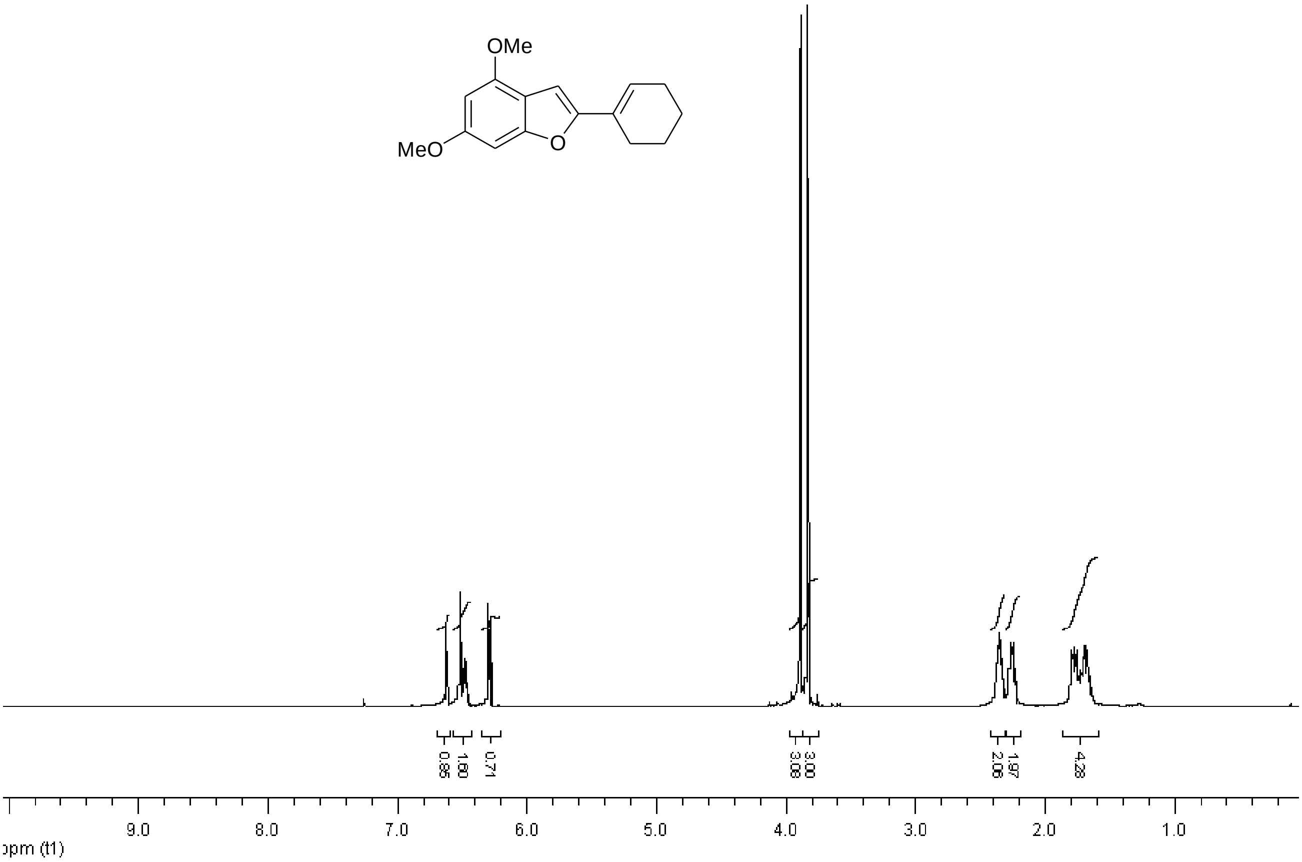

^{13}C NMR (75.4 MHz, $CDCl_3$):

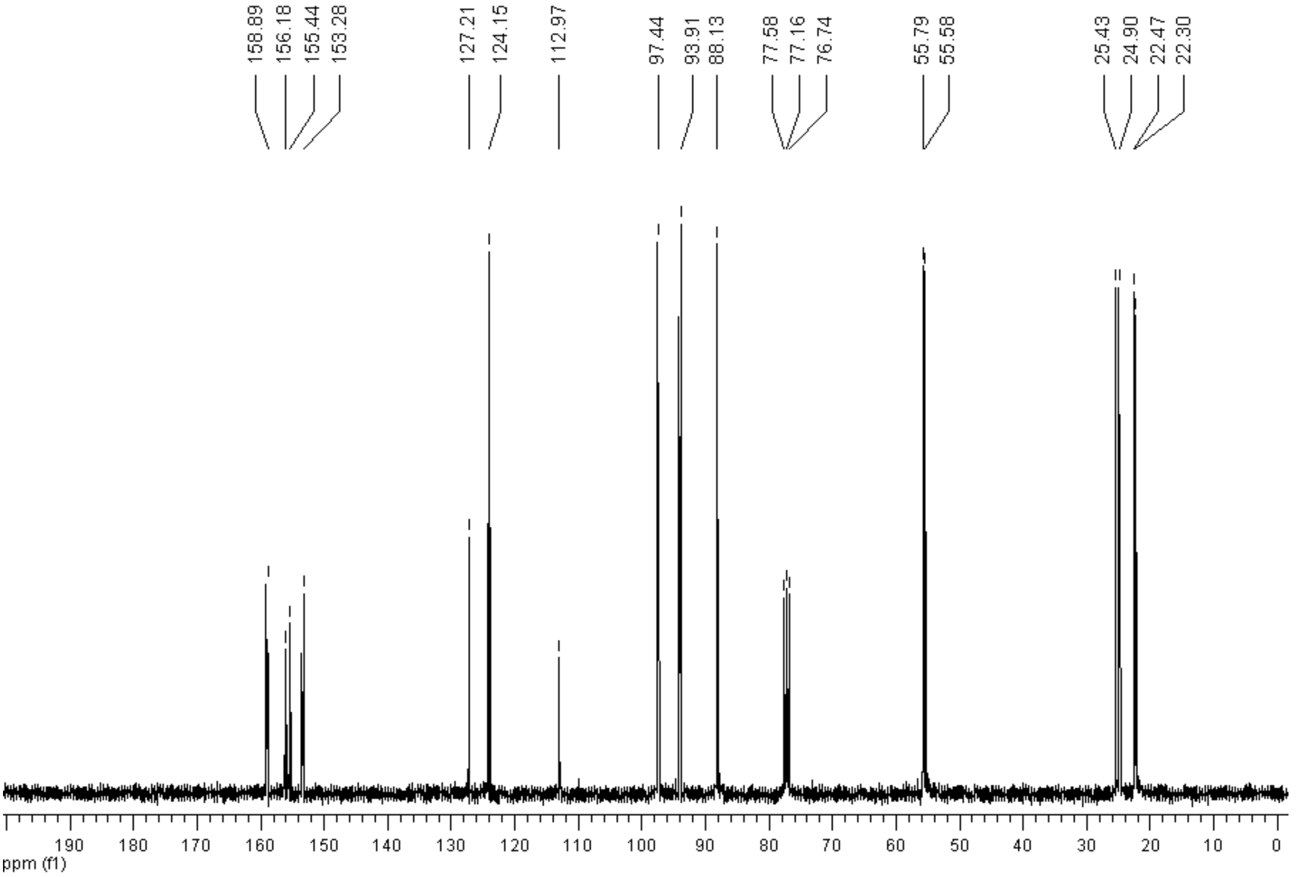

^{1}H NMR (300 MHz, $CDCl_3$) (9c):

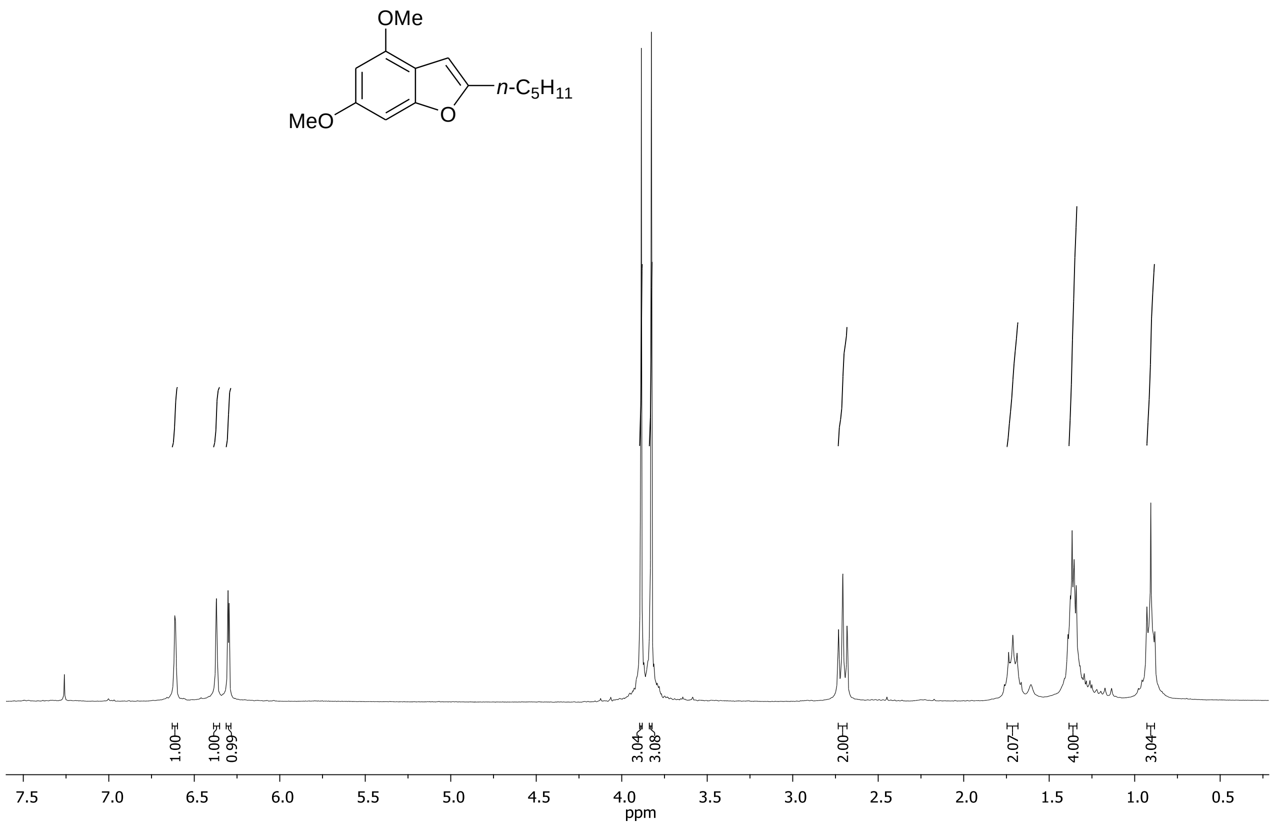

^{13}C NMR (75.4 MHz, $CDCl_3$):

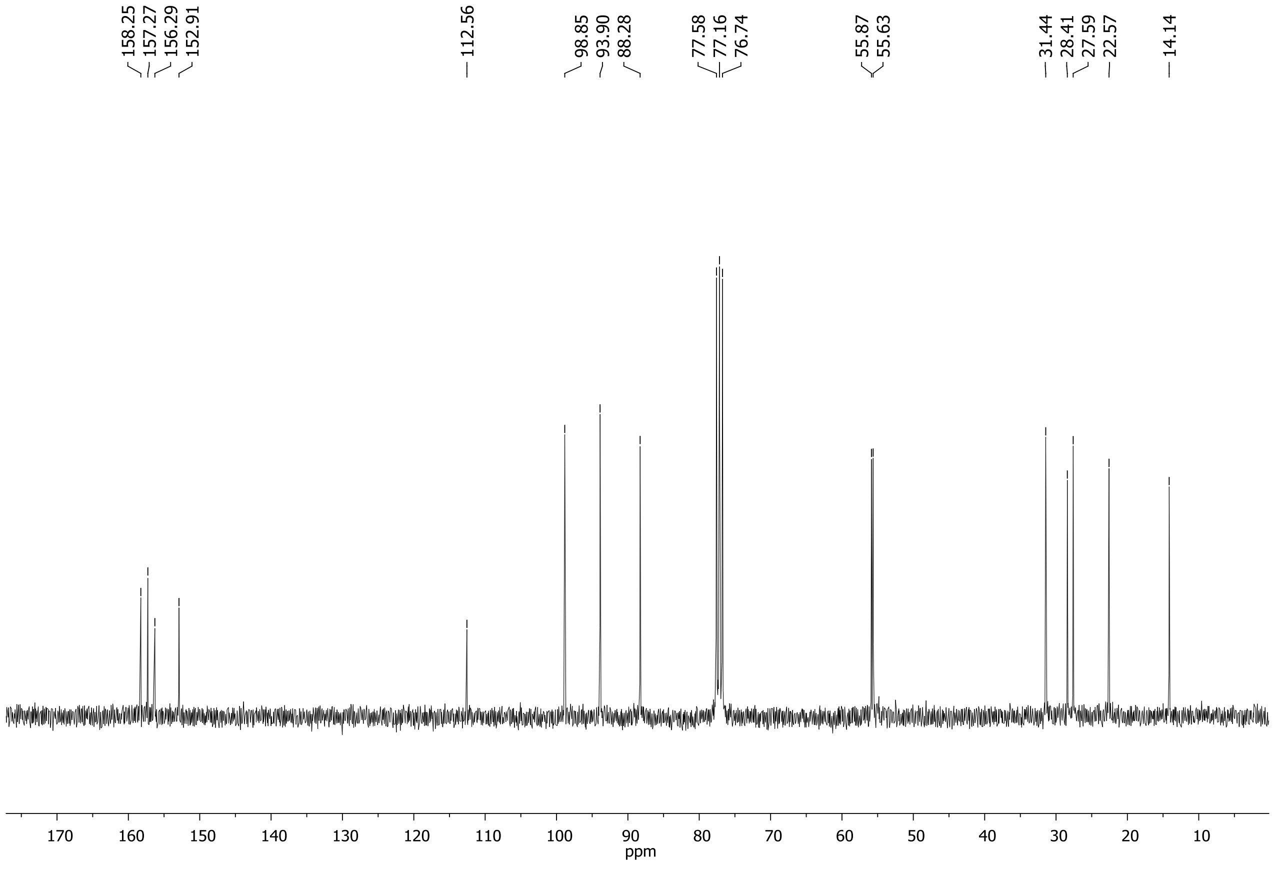

^{1}H NMR (300 MHz, $CDCl_3$) (9d):

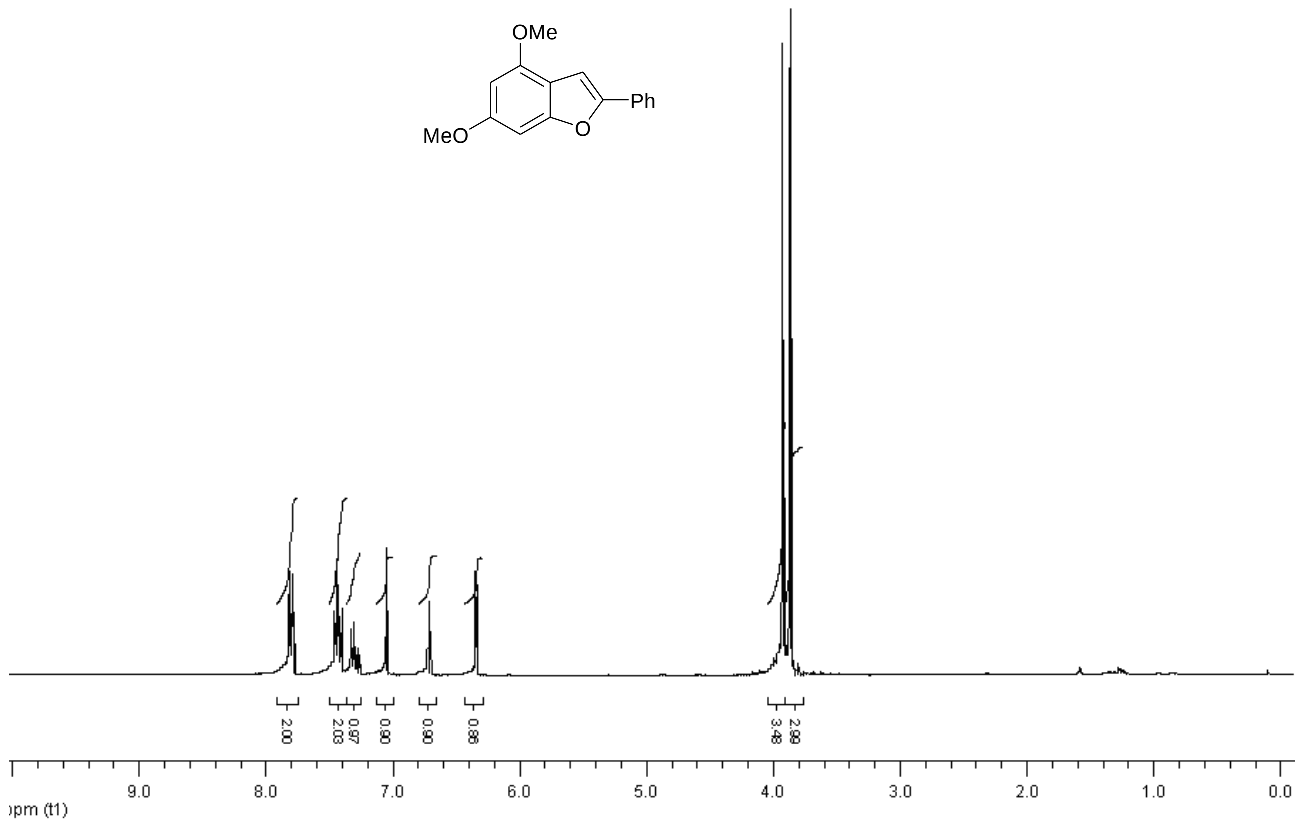

^{13}C NMR (75.4 MHz, $CDCl_3$):

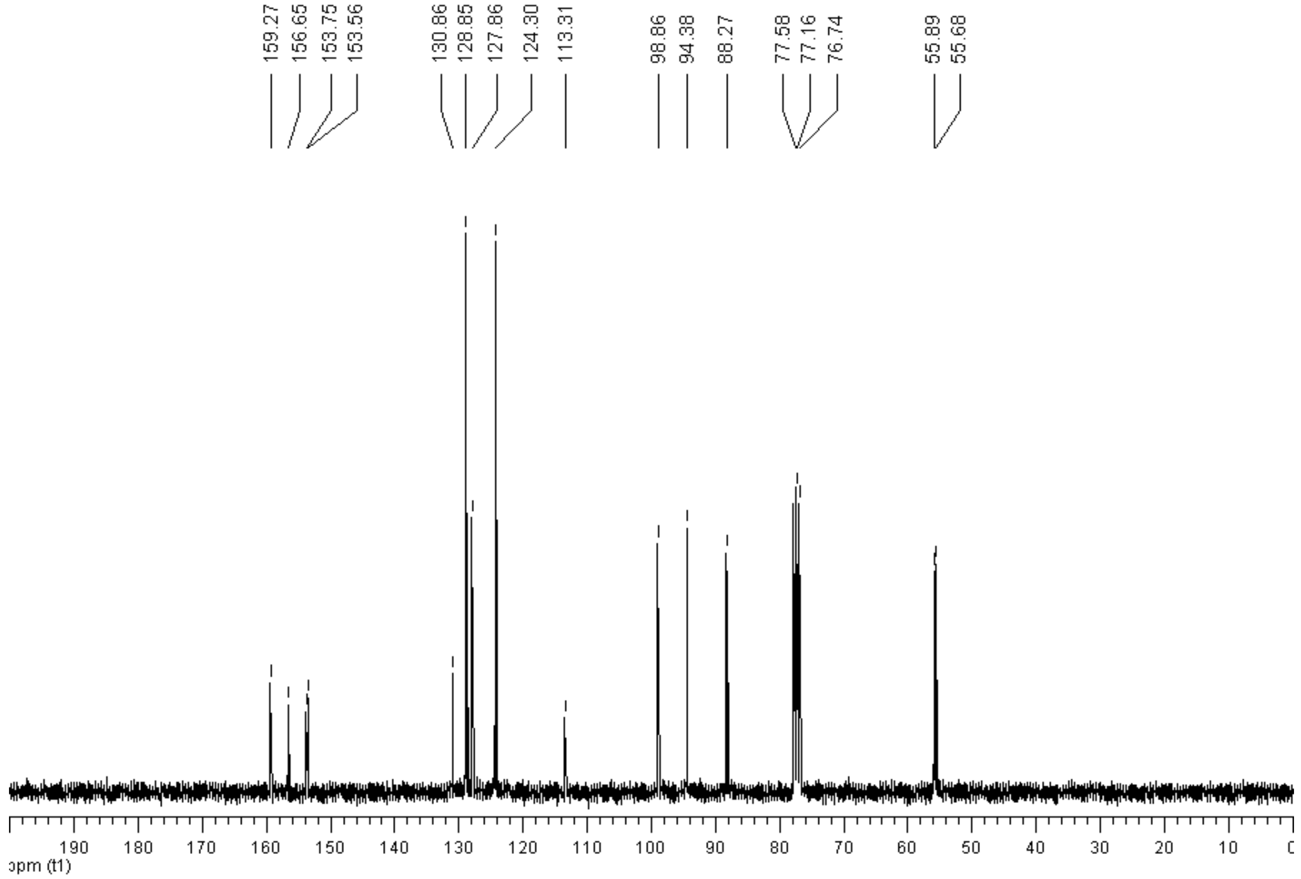

^{1}H NMR (300 MHz, $CDCl_3$) (9e):

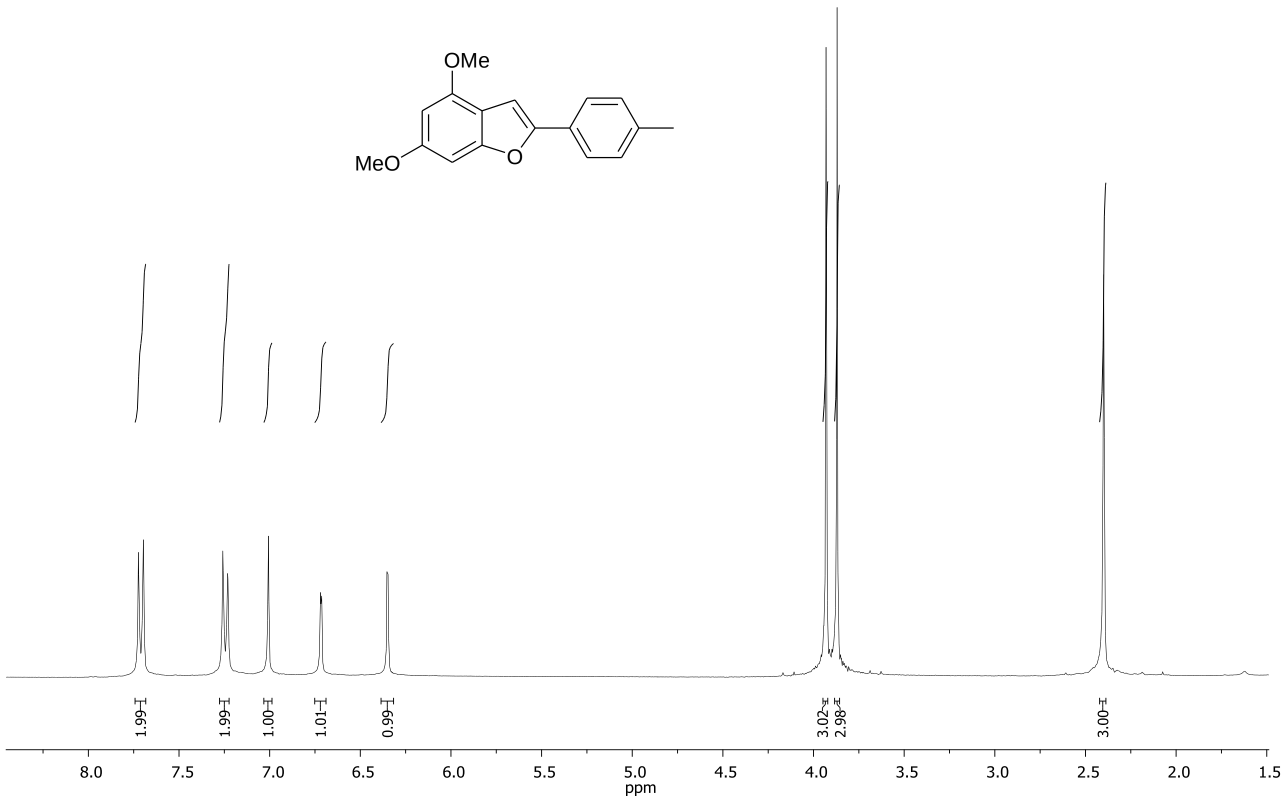

^{13}C NMR (75.4 MHz, $CDCl_3$):

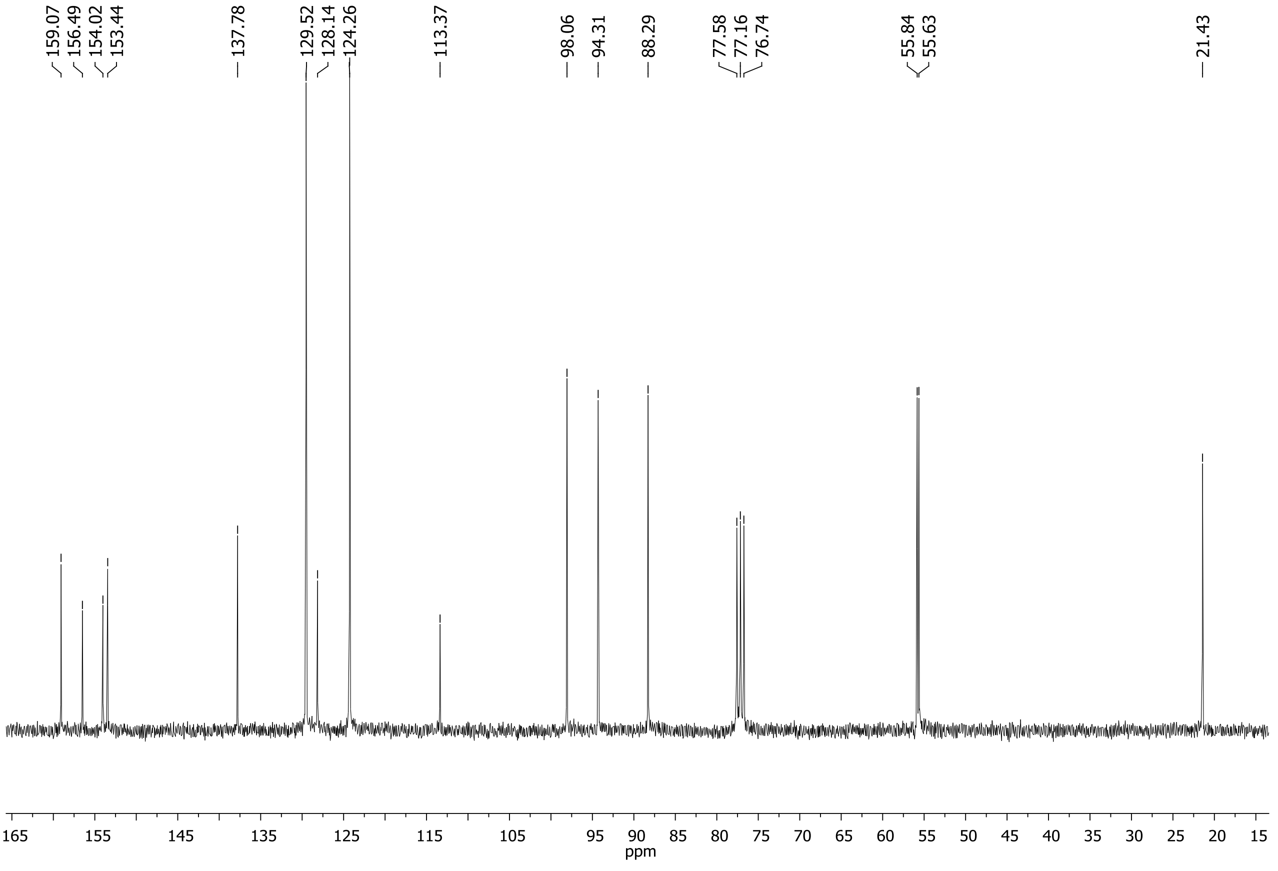

^{1}H NMR (300 MHz, $CDCl_3$) (10a):

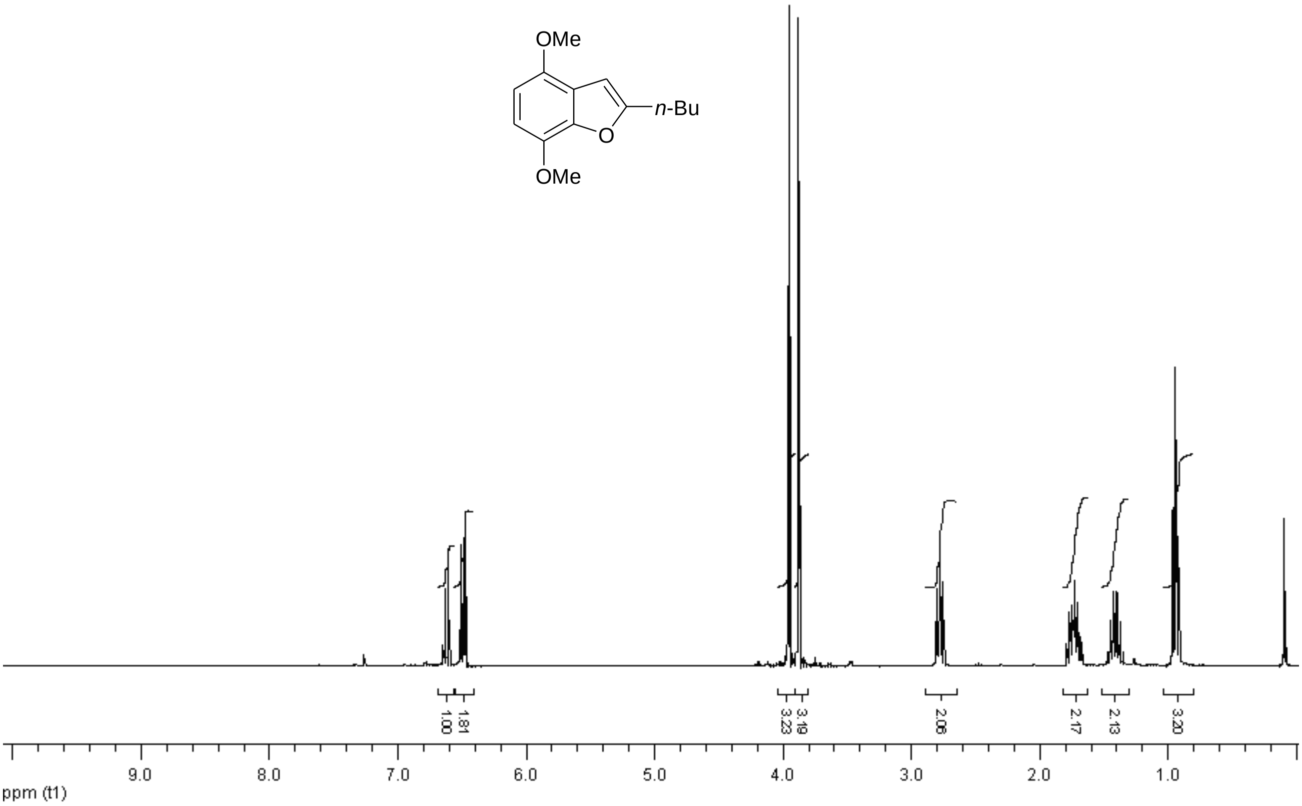

^{13}C NMR (75.4 MHz, $CDCl_3$):

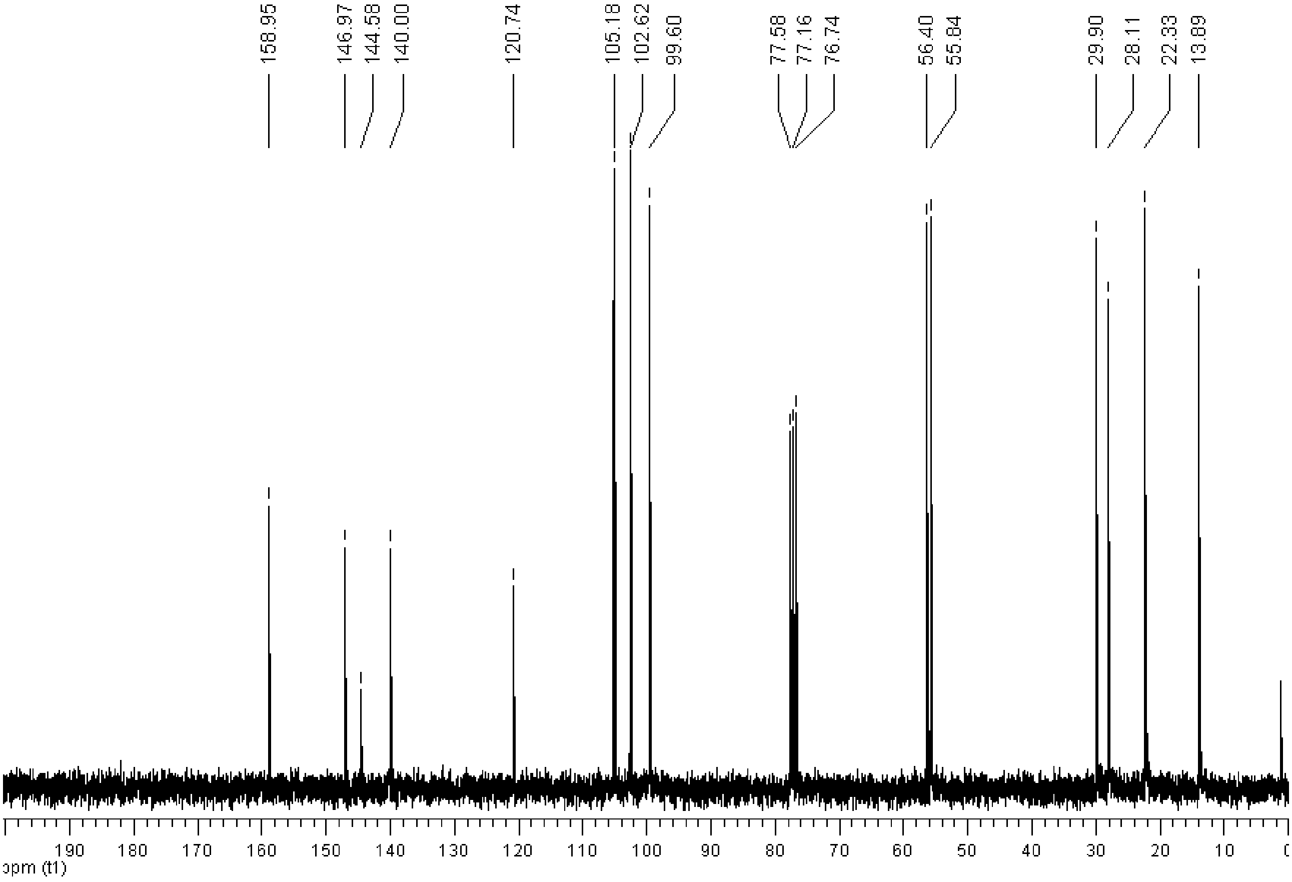

^{1}H NMR (300 MHz, $CDCl_3$) (10b):

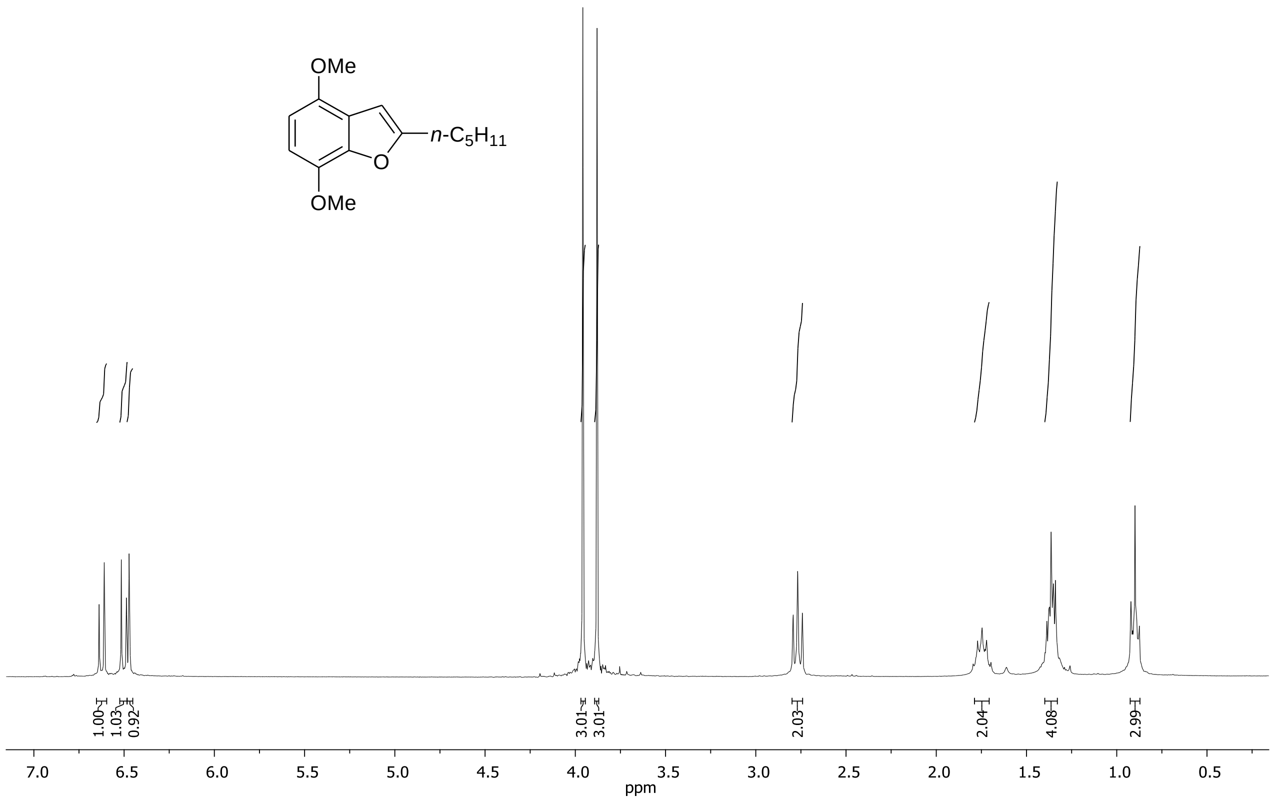

^{13}C NMR (75.4 MHz, $CDCl_3$):

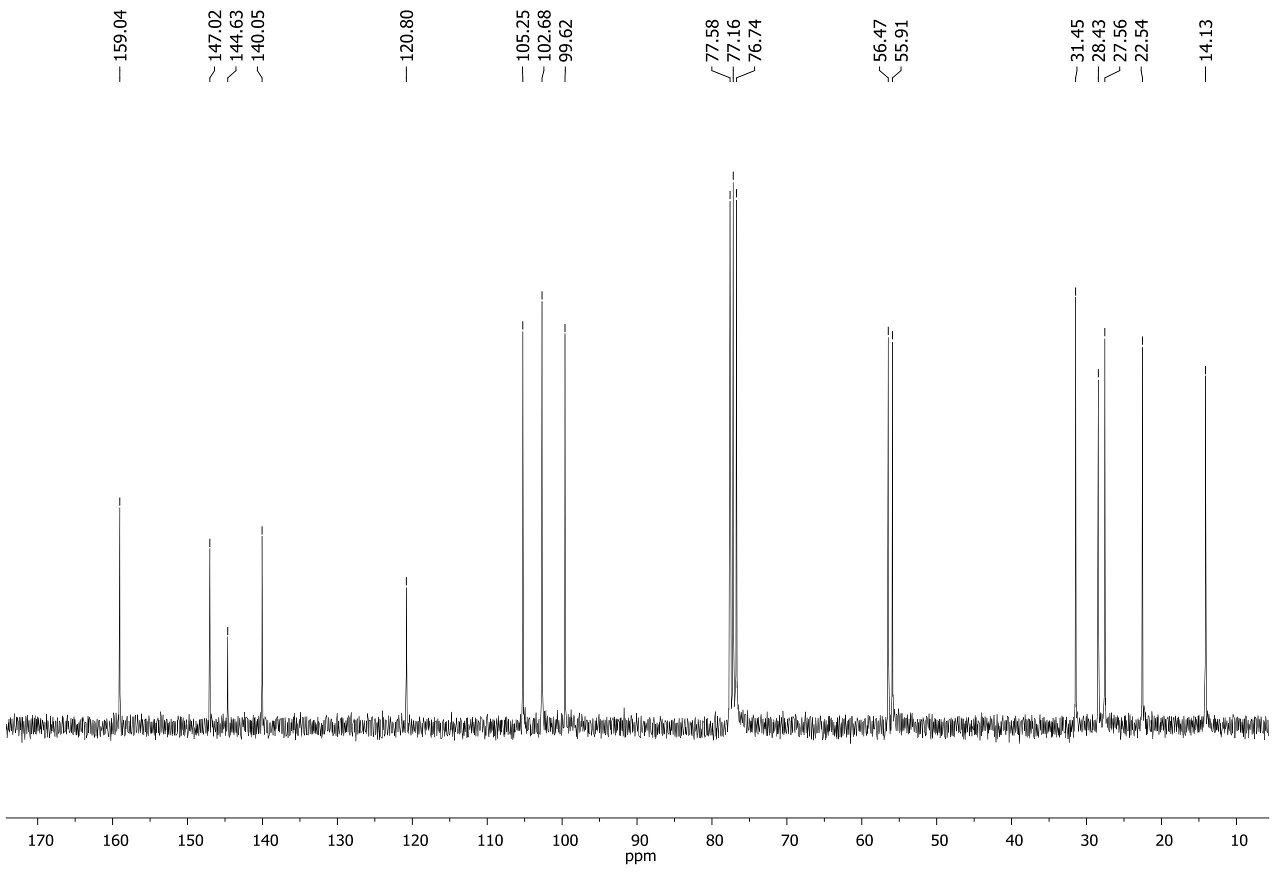

^{1}H NMR (300 MHz, $CDCl_3$) (10c):

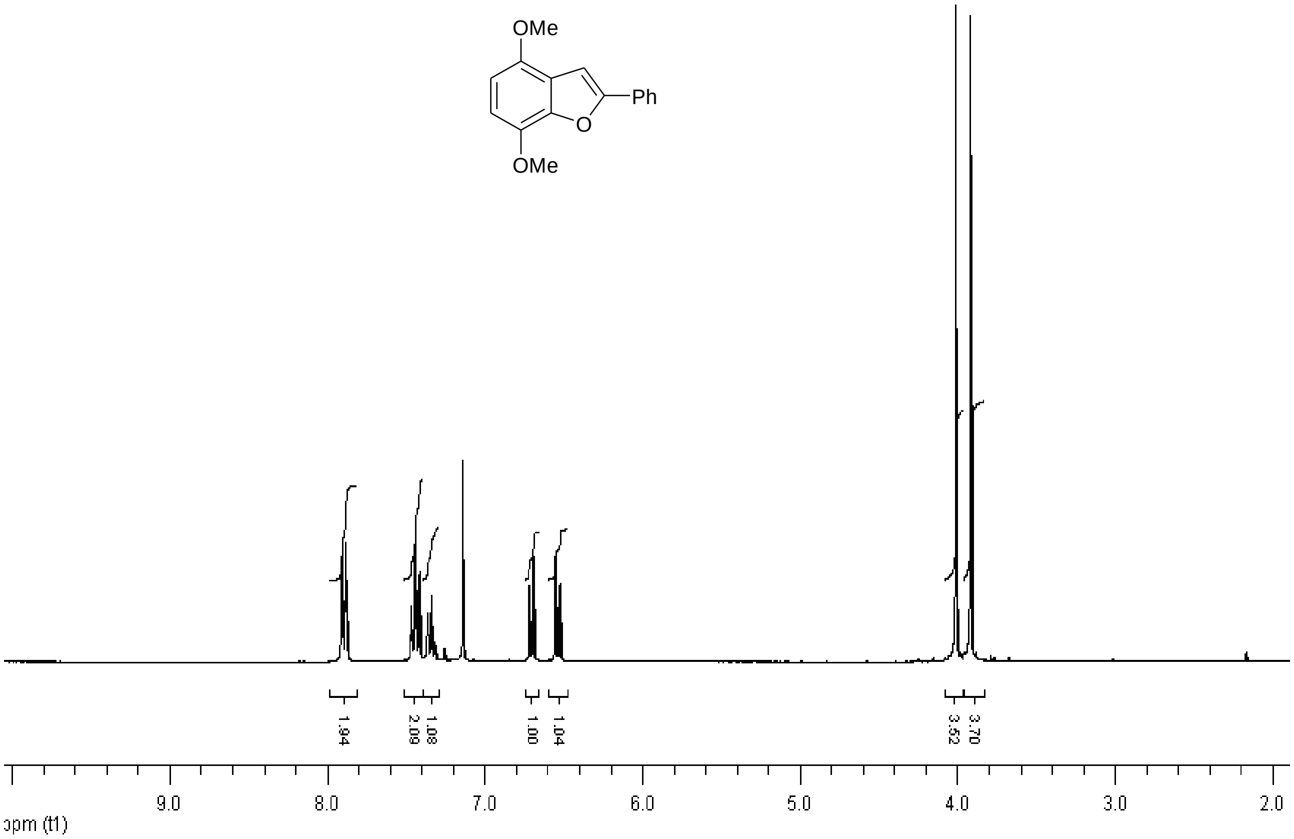

^{13}C NMR (75.4 MHz, $CDCl_3$):

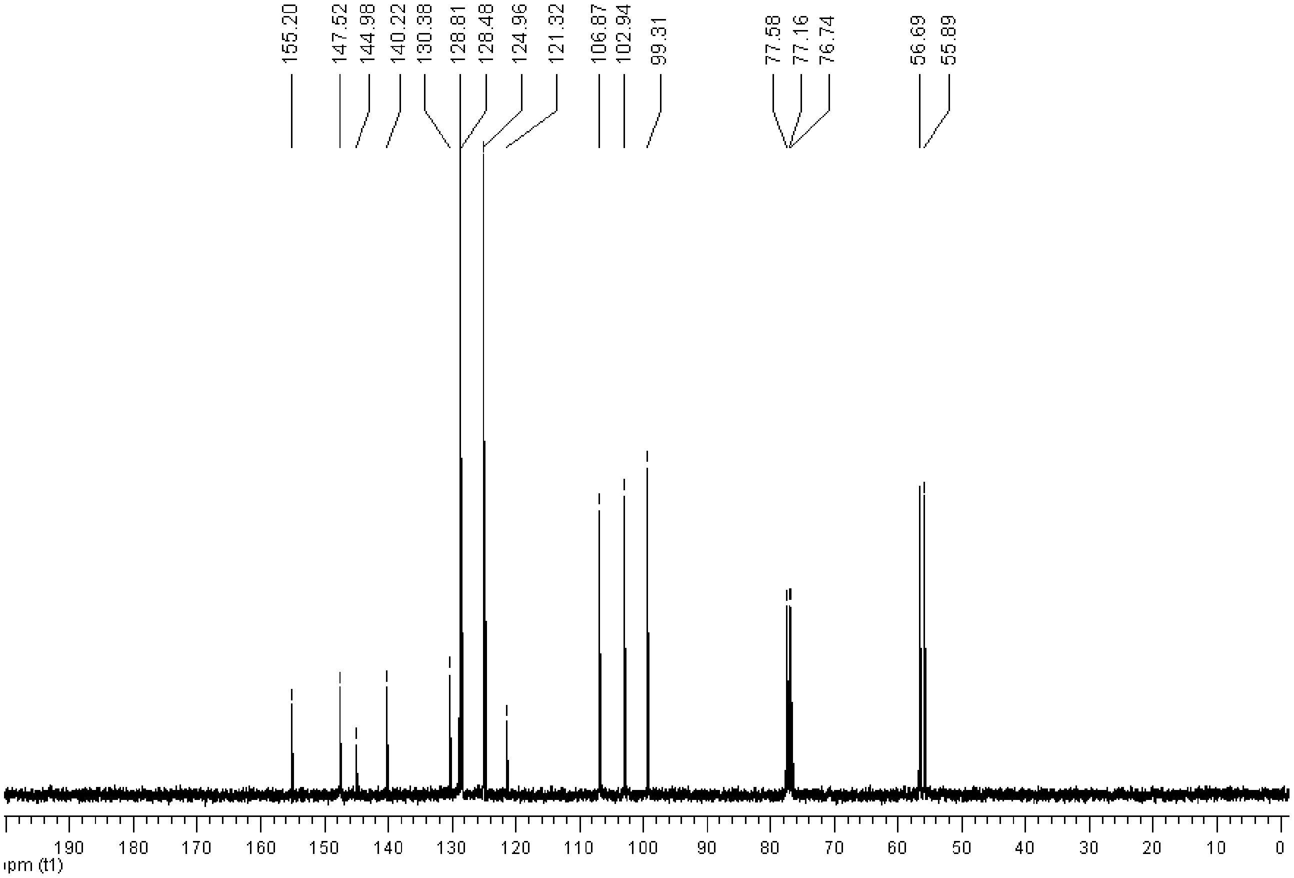

^{1}H NMR (300 MHz, $CDCl_3$) (10d):

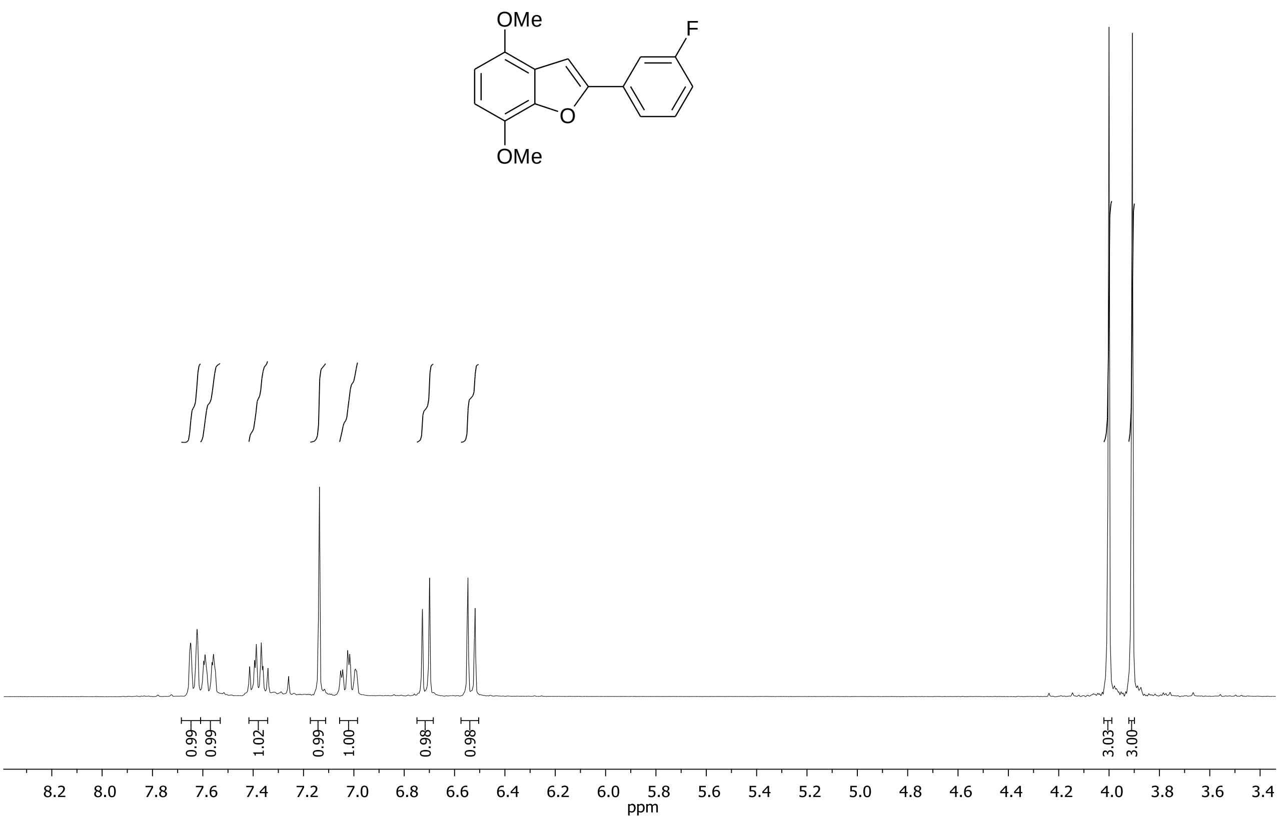

^{13}C NMR (75.4 MHz, $CDCl_3$):

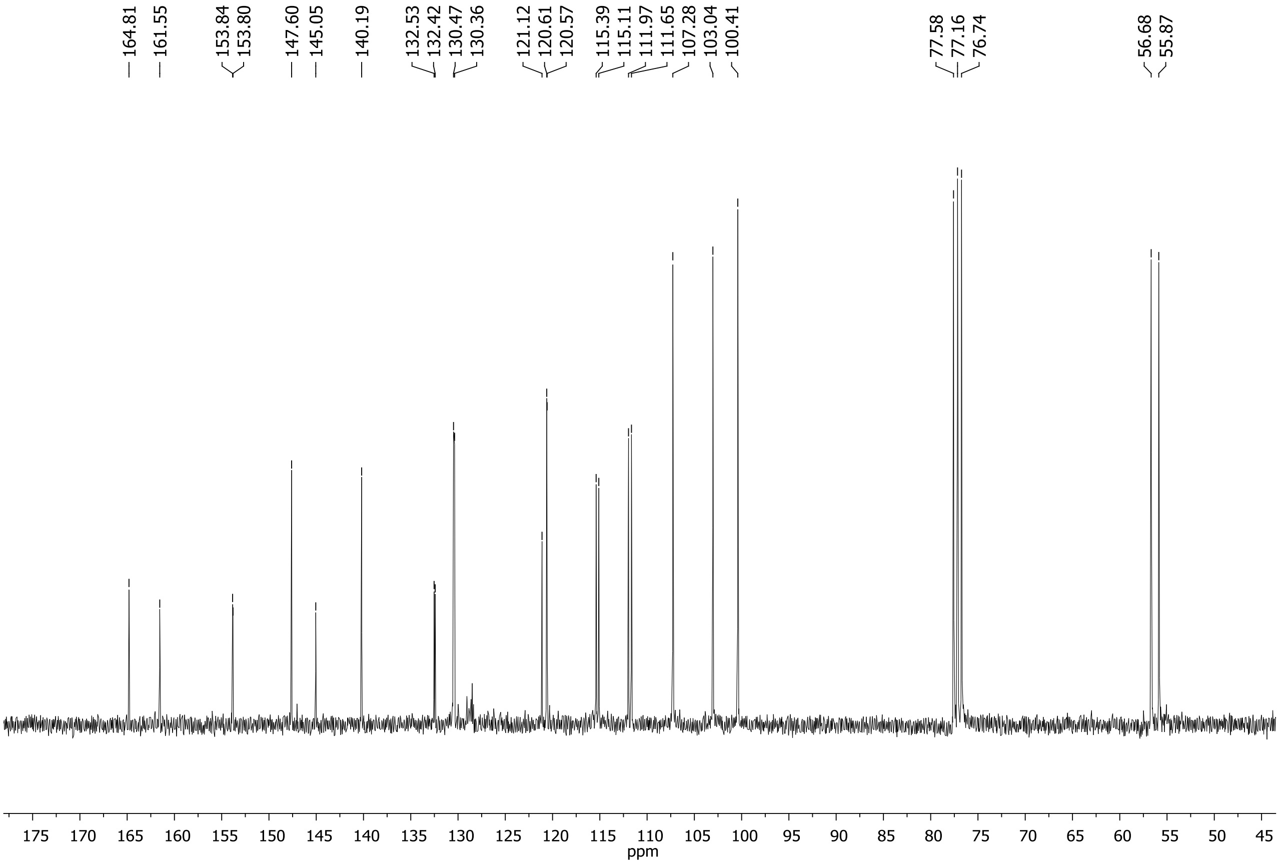

Supplement: File 2 — NMR spectra. [file Beilstein_J_Org_Chem-07-1255-s002.pdf]
